# The evolution of the vertebrate metzincins; insights from *Ciona intestinalis *and *Danio rerio*

**DOI:** 10.1186/1471-2148-7-63

**Published:** 2007-04-17

**Authors:** Julie Huxley-Jones, Toni-Kim Clarke, Christine Beck, George Toubaris, David L Robertson, Raymond P Boot-Handford

**Affiliations:** 1 Wellcome Trust Centre for Cell-Matrix Research, Faculty of Life Sciences, University of Manchester, Manchester M13 9PT, UK; 2 Institute of Psychiatry, Kings College London, De Crespigny Park, London SE5 8AF, UK

## Abstract

**Background:**

The metzincins are a large gene superfamily of proteases characterized by the presence of a zinc protease domain, and include the ADAM, ADAMTS, BMP1/TLL, meprin and MMP genes. Metzincins are involved in the proteolysis of a wide variety of proteins, including those of the extracellular matrix. The metzincin gene superfamily comprises eighty proteins in the human genome and ninety-three in the mouse. When and how the level of complexity apparent in the vertebrate metzincin gene superfamily arose has not been determined in detail. Here we present a comprehensive analysis of vertebrate metzincins using genes from both *Ciona intestinalis *and *Danio rerio *to provide new insights into the complex evolution of this gene superfamily.

**Results:**

We have identified 19 metzincin genes in the ciona genome and 83 in the zebrafish genome. Phylogenetic analyses reveal that the expansion of the metzincin gene superfamily in vertebrates has occurred predominantly by the simple duplication of pre-existing genes rather than by the appearance and subsequent expansion of new metzincin subtypes (the only example of which is the meprin gene family). Despite the number of zebrafish metzincin genes being relatively similar to that of tetrapods (e.g. man and mouse), the pattern of gene retention and loss within these lineages is markedly different. In addition, we have studied the evolution of the related TIMP gene family and identify a single ciona and four zebrafish TIMP genes.

**Conclusion:**

The complexity seen in the vertebrate metzincin gene families was mainly acquired during vertebrate evolution. The metzincin gene repertoire in protostomes and invertebrate deuterostomes has remained relatively stable. The expanded metzincin gene repertoire of extant tetrapods, such as man, has resulted largely from duplication events associated with early vertebrate evolution, prior to the sarcopterygian-actinopterygian split. The teleost repertoire of metzincin genes in part parallels that of tetrapods but has been significantly modified, perhaps as a consequence of a teleost-specific duplication event.

## Background

**Figure 1:**
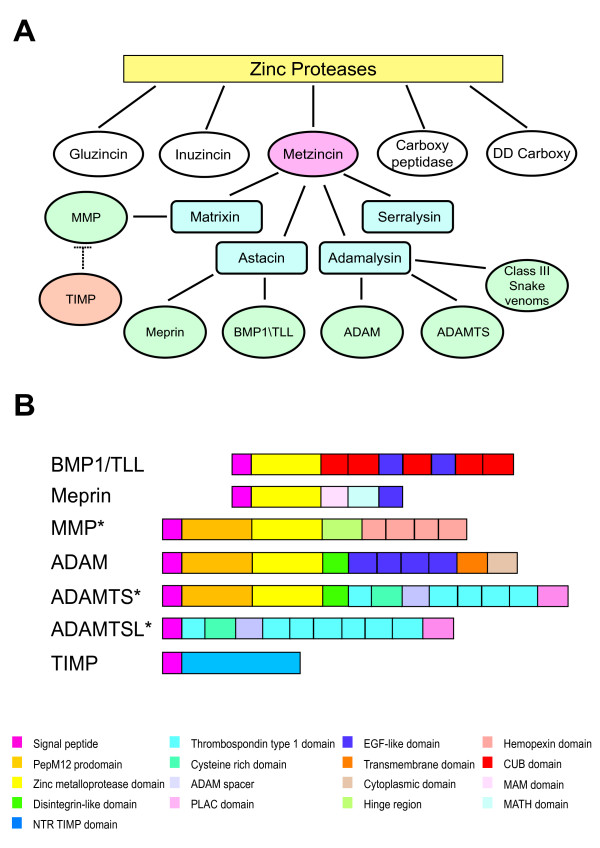
The metzincin gene family. A. Schematic representation of subdivisions within the Metzincin superfamily. B. Domain structure of generic metzincin genes. All ADAM, BMP/TLL, Meprin and TIMP genes have the same domain structure as that shown on the figure. * The ADAMTSL domain structure shown is ADAMTSL2. ADAMTS, ADAMTSL and MMMP genes have a variable C-terminal domain structure. The domain structures shown are ADAMTS10, ADAMTSL2 and MMP1.

Extracellular matrix (ECM) components modulate cellular behaviour by creating influential cellular environments. The processing and turnover of ECM is integral to providing the correct environment to support and direct development, morphogenesis and tissue remodelling [1]. The metzincin proteases, mainly involved in proteolysis of extracellular matrix proteins, are a gene superfamily characterized by a protease domain with a HEXXHXXGXXH zinc-binding motif at the active site [2]. The superfamily can be subdivided into four subfamilies according to subtle differences in the catalytic site and the presence of additional domains: matrixins (MMPs), adamalysins (ADAM, ADAMTS and class III snake venom proteins), astacins (BMP1/TLL proteins and meprins) and bacterial serralysins (Fig. 1A). The focus of this study is to investigate the evolutionary history of the vertebrate metzincin superfamily.

ADAM proteins consist of a prodomain, followed by metalloprotease, disintegrin, cysteine rich, EGF, transmembrane and cytoplasmic domains (Fig. 1B). Approximately half of the ADAMs identified in mammals are believed to be proteolytically active and many are thought to play roles in adhesion [4]. Proteolytically active ADAMs are membrane-bound enzymes that act as molecular switches by cleaving and releasing proteins from the cell surface through a process known as ectodomain shedding [3]. Produced as zymogens, cleavage by proprotein convertase (e.g. furin) is required for activation of ADAMs [4]. ADAM proteolysis acts upon a wide variety of growth factors, cytokines and receptors and is implicated in various cell behaviours such as angiogenesis, fertilization and neurogenesis [3]. For example, ADAM17 functions as a TNFα converting enzyme [5], is required for the activation of EGF receptor ligands such as TGFα [6] and plays a prominent role in the activation of the Notch signaling pathway [7]. The recent finding that ADAM22 acts as a receptor for LGI1 in regulating synaptic transmission across the neuronal membrane [8], suggest that ADAM proteins can exhibit functions separate to that of proteolysis.

ADAMTS share the same domain structure of ADAM genes, except that they lack the transmembrane and cytoplasmic domains, and in addition contain a large variable ancillary region characterised by thrombospondin type 1 repeats (Fig. 1B) [9]. ADAMTS are secreted proteases whose ancillary domains have been considered to determine substrate specificity, since the protease domains alone appear to be unable to process native substrates [10]. Synthesised as proproteins, the removal of the prodomain occurs in the secretory pathway through the action of proprotein convertases [11]. The processing of extracellular matrix molecules such as procollagens, aggrecan and von Willebrand Factor by ADAMTS proteins impacts on developmental processes such as angiogenesis, coagulation and connective tissue organisation, and diseases such as arthritis and inflammation [11]. Included within the ADAMTS family is a small group of ADAMTS-like proteins that lack the metalloprotease and disintegrin-like domains [12-14]. The ADAMTS-like proteins have been proposed to play roles in regulating cell-matrix interactions [14].

The BMP1 and tolloid-like genes consist of the zinc metalloprotease domain followed by CUB and calcium-binding EGF-like domains (Fig. 1B). In a similar manner to that of the ADAM and ADAMTS genes, BMP1 is synthesised with an N-terminal prodomain, which is cleaved by proprotein convertases during protein maturation [15]. In addition to their roles in the maturation of numerous extracellular matrix proteins, such as fibrillar collagens, small leucine rich proteoglycans and lysyl oxidase [16], BMP/TLL genes play key roles in the activation of TGF-β-like ligands and the cleavage of chordin [17]. BMP1/TLL genes are widely expressed and have major developmental roles in early embryogenesis [16].

Meprins are multidomain metalloproteases encoded by two vertebrate genes, MEP1A and MEP1B. In addition to the metalloprotease domain, the meprins also contain a MAM (meprin A5 protein tyrosine phosphatase *μ*) domain, a MATH (meprin and TRAF [tumour necrosis factor receptor associated factor] homology) domain and a calcium-binding EGF-like domain at the C-terminus (Fig. 1B). Secreted as zymogens, meprins are activated by plasmin [18] and trypsin in the intestine [19]. Highly expressed in mammalian kidney and intestine, meprins are capable of cleaving a wide variety of hormones [20] as well as extracellular matrix proteins such as fibronectin, laminin and collagen [21,22]. Meprins are also expressed by leukocytes and are thought to be involved in cell migration during immune responses [23].

The class III snake venoms, characterised as members of the metzincin gene superfamily [2], have evolved in a lineage specific manner distinct from the other vertebrate metzincins and since their detailed phylogeny has been previously determined [24] they will not be considered further here.

Matrix metalloproteases (MMPs) consist of a propeptide, the zinc metalloprotease domain, a linker or hinge region and C-terminal hemopexin domains (Fig. 1B). MMPs are classified in the basis of their substrate specificity and include collagenases, gelatinases and stromelysins. Secreted in a latent form, most MMPs are activated following cleavage by extracellular proteases [25], some of which being MMPs located at the cell surface known as membrane-type MT-MMPs (MMPs14, 15, 16, 17, 24 and 25). Indeed all MT-MMPs, except MMP17, can activate proMMP2 [26]. The MMP proteins are involved in the breakdown of extracellular matrix in normal physiological processes such as embryonic development, tissue remodelling and reproduction, as well as in disease processes such as arthritis and cancer [26].

TIMPs are the endogenous regulators of ADAM, ADAMTS and MMP genes [26-28], and have been included in this analysis due to this relationship with the metzincin family. TIMPs have a single NTR (netrin) domain structure consisting of a 125 amino acid N-terminus and a smaller C-terminal region (Fig. 1B) forming a "wedge-like" structure [26]. The inhibition of MMPs by TIMP proteins occurs when the reactive ridge of the TIMP slots into the active site of the MMP [26]. The expression of TIMP genes is regulated to maintain a balance of tissue remodelling and degradation in the extracellular matrix, disruption of which can lead to a variety of diseases such as cancer and arthritis [29]. TIMP function has also been implicated in the promotion of cell proliferation in a variety of cell types [30,31]. Mutations in TIMP3 are associated with Sorsby's fundus dystrophy, which results in early onset macular degeneration [32].

The sequencing of the genome of the ascidian *Ciona intestinalis *[33], a urochordate and one of the closest invertebrate relatives of vertebrates, provides a unique opportunity to gain insight into the complete set of metzincins available in chordates prior to the large-scale or whole genome duplication events that many believe were associated with the early stages of vertebrate evolution [34-36]. The zebrafish (*Danio rerio*) genome has also been investigated since comparative studies provide insight into the likely timing of duplications occurring during vertebrate evolution. Gene duplications shared by fish and man are likely to have occurred prior to the tetrapod/teleost divergence, whereas duplications unique to one of the lineages are most likely to have occurred after their divergence approximately 350 million years ago [37]. In addition, the identification of zebrafish metzincin orthologues may provide insights into the putative third whole genome duplication event, proposed to have occurred within the actinopterygian lineage [37].

The metzincin gene superfamily comprises eighty proteins in the human genome and ninety-three in the mouse. When and how the level of complexity apparent in the vertebrate metzincin superfamily arose has not been determined in detail. We have previously identified *Ciona intestinalis *orthologues for the ADAMTS and BMP1/Tolloid-like gene families [38,39]. Here we present a comprehensive analysis of the vertebrate metzincin gene superfamily using genes from both *Ciona intestinalis *and *Danio rerio *to provide new insights into the complex evolution of this gene superfamily.

## Results

### Identification of metzincin genes in the ciona and zebrafish genomes

A total of nineteen genes encoding metzincins were identified in the ciona genome (Table 1 & Additional File 1 &2) comprising four ADAM, seven MMP together with the previously reported seven ADAMTS and single BMP1/tolloid gene [38,39]. Meprin orthologues were not found in the ciona genome. A single ciona TIMP orthologue was identified (Table 1).

**Table 1:** Metzincin genes in the *Ciona intestinalis *genome and their direct human orthologues.

**Ciona gene**	**Human orthologue**	**Human Locus**	**Figure**
***ADAM***			
**ADAMa**	ADAM17	2p25	2
**ADAMb**	ADAM10	15q22	2
**ADAMc1 & cc2**	ADAM2	8p11.2	2
	ADAM7	8p21.2*	2
	ADAM8	10q26.3*	2
	ADAM9	8p11.23	2
	ADAM11	17q21.3*	2
	ADAM12	10q26.3*	2
	ADAM15	1q21.3*	2
	ADAM18	8p11.22	2
	ADAM19	5q32-q33*	2
	ADAM22	7q21*	2
	ADAM23	2q33*	2
	ADAM28	8p21.2*	2
	ADAM32	8p11.23	2
	ADAM33	20p13*	2
	ADAMDEC1	8p21.2*	2
***ADAMTS***			
**ADAMTSa**	ADAMTS2	5qter	3A
	ADAMTS3	4q21.1*	3A
	ADAMTS14	10q2*	3A
**ADAMTSb**	ADAMTS16	5p35	3A
	ADAMTS18	16q34	3A
**ADAMTSc**	ADAMTS7	15q24.2	3A
	ADAMTS12	5q35	3A
**ADAMTSd**	ADAMTS9	3p14.3-p14.2	3A
	ADAMTS20	12q12	3A
**ADAMTSe**	ADAMTS6	5pter-qter*	3A
	ADAMTS10	19p13.1*	3A
**ADAMTSf**	ADAMTS1	21q21.2*	3A
	ADAMTS4	1q21-q23*	3A
	ADAMTS5	21q21.3*	3A
	ADAMTS8	11q25*	3A
	ADAMTS15	11q25*	3A
**ADAMTSg**	Papilin	14q24.2	3B
	ADAMTSL1	9p22.1*	3B
	ADAMTSL2	9q34.2	3B
	ADAMTSL3	15q25*	3B
	ADAMTSL4	1q21.2	3B
	ADAMTSL5	19p13.3	3B
***BMP1/TLL***			
**BMP1/TLL**	BMP-1	8p21	3C
	Tolloid-like 1	4q32-q33	3C
	Tolloid-like 2	10q23-q24	3C
***MMP***			
**MMPa1, a2 & MMPa3**	MMP19	12q14	4
	MMP26	11p15	4
	MMP28	17q11-q21.1	4
**MMPb**	MMP21	10q26.2	4
**MMPc**	MMP17	12q24.3	4
	MMP25	16p13.3	4
	MMPL1	16p13.3	4
**MMPd**	MMP14	14q11-q12*	4
	MMP15	16q13-q21*	4
	MMP16	8q21*	4
	MMP24	20q11.2*	4
**MMPe**	MMP2	16q13-q21*	4
	MMP7	11q21-q22	4
	MMP9	20q11.2-q13.1*	4
	MMP20	11q22.3	4
***TIMP***			
**TIMP**	TIMP1	Xp11.3-p11.23*	3E
	TIMP2	17q25*	3E
	TIMP3	22q12.3*	3E
	TIMP4	3p25*	3E

Ciona genes listed with their direct human orthologues, produced from the associated phylogenetic analysis listed. * indicates that related genes within the sub-clade are located in paralogous regions of the human genome.

In contrast, eighty-three metzincin genes were identified in the zebrafish genome (Table 2 & Additional File 3). These consisted of twenty-two ADAM, twenty-seven ADAMTS, four BMP1/tolloid, four meprin and twenty-six MMP orthologues (Table 2). In addition, four zebrafish TIMP orthologues were identified (Table 2).

**Table 2:** Metzincin genes in the *Danio rerio *genome and their direct ciona and human orthologues.

**Zebrafish gene**	**Human orthologue**	**Figure**
***ADAM***		
**A clade**		
ADAM10a	ADAM10	2 & S1
ADAM10b	ADAM10	2 & S1
ADAM17a	ADAM17	2 & S1
ADAM17b	ADAM17	2 & S1
**B clade**		
ADAM8a	ADAM8	2& S2
ADAM8b	ADAM8	2 & S2
ADAM9^a^	ADAM9	S2
ADAM11	ADAM11	2 & S2
ADAM12a	ADAM12	2 & S2
ADAM12b ^a^	ADAM12	2 S2
ADAM12c ^a^	ADAM12	2 & S2
ADAM15	ADAM15	2 & S2
ADAM19a	ADAM19	2 & S2
ADAM19b ^a^	ADAM19	S2
ADAM22	ADAM22	2 & S2
ADAM23a	ADAM23	2 & S2
ADAM23b	ADAM23	2 & S2
ADAM28	ADAM28	2 & S2
ADAMLa		2 & S2
ADAMLb ^b^		2
ADAMLc ^b^		2
ADAMLd ^b^		2
***ADAMTS***		
ADAMTS1	ADAMTS1	3A
ADAMTS2/3	ADAMTS2 and ADAMTS3	3A
ADAMTS5	ADAMTS5	3A
ADAMTS8a	ADAMTS8	3A
ADAMTS8b	ADAMTS8	3A
ADAMTS8c	ADAMTS8	3A
ADAMTS8d	ADAMTS8	3A
ADAMTS9	ADAMTS9	3A
ADAMTS12	ADAMTS12	3A
ADAMTS13	ADAMTS13	3A
ADAMTS15a	ADAMTS15	3A
ADAMTS15b	ADAMTS15	3A
ADAMTS15c	ADAMTS15	3A
ADAMTS18	ADAMTS18	3A
ADAMTSL2a	ADAMTSL2	3B
ADAMTSL2b	ADAMTSL2	3B
ADAMTSL2c	ADAMTSL2	3B
ADAMTSL4	ADAMTSL4	3B
ADAMTSL5	ADAMTSL5	3B
PAPLNa	Papilin	3B
PAPLNb	Papilin	3B
ADAMTSLa		3B
ADAMTSLb		3B
ADAMTSLc		3B
ADAMTSLd		3B
ADAMTSLe		3B
ADAMTSLf		3B
***BMP1/TLL***		
BMP1a	BMP1	3C
BMP1b	BMP1	3C
BMP1c	BMP1	3C
TLL1	TLL1	3C
***Meprin***		
MEP1Aa	MEP1A	3D
MEP1Ab	MEP1A	3D
MEP1Ac	MEP1A	3D
MEP1B	MEP1B	3D
***MMP***		
**B clade**		
MMP11a	MMP11	4 & S5
MMP11b	MMP11	4 & S5
MMP23a	MMP23A & B	4 & S5
MMP23b	MMP23A & B	S5
**C clade**		
MMP17	MMP17	4 & S6
MMPLa		4 & S6
MMPLb		4 & S6
MMPLc		4 & S6
MMPLd		4 & S6
**D clade**		
MMPLe	MMP1/3/8/10/12/13/27	4 & S7
**E clade**		
MMP14a	MMP14	4 & S8
MMP14b	MMP14	4 & S8
MMP14c	MMP14	4 & S8
MMP15a	MMP15	4 & S8
MMP15b	MMP15	4 & S8
MMP15c	MMP15	4 & S8
MMP16a	MMP16	4 & S8
MMP16b	MMP16	4 & S8
MMP24a	MMP24	4 & S8
MMP24b	MMP24	4 & S8
**F clade**		
MMP2	MMP2	4 & S9
MMP7	MMP7	S9
MMP9	MMP9	4 & S9
MMPLf		4 & S9
MMPLg		4 & S9
MMPLh		4 & S9
***TIMP***		
TIMP2a	TIMP2	3E
TIMP2b	TIMP2	3E
TIMP2c	TIMP2	3E
TIMP2d	TIMP2	3E

Zebrafish genes listed with their direct ciona and human orthologues, produced from the associated phylogenetic analysis listed. ^a ^very short sequences which on limited phylogenetic analysis clearly grouped with the orthologues indicated, however insufficient data to include in Figure 3. ^b ^could not be accurately grouped into sub-clades for subsequent analysis following production of guide tree (Fig. S1). Supplementary Figures are located in additional file 4.

The majority of the identified gene sequences were annotated but many were fragmented. Where possible, these sequences were further refined by cross-reference to EST databases and direct searching, and analysis of flanking genomic sequence; amended sequences used in the following analyses are reported in full in Additional Files 2 (ciona) and 3 (zebrafish) respectively.

**Figure 2:**
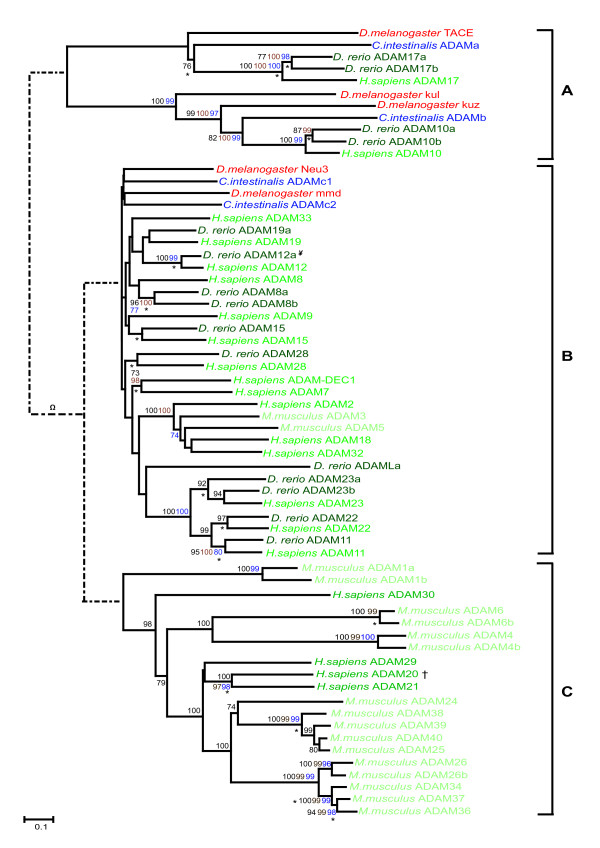
Phylogenetic relationships of the ADAM gene family. The ADAM gene family was separated into three sub-analyses, indicated A, B and C, based upon the clades produced and independent phylogenetic analyses performed. The trees shown were inferred by Neighbor Joining from a gapped alignment. The values on the tree nodes are neighbor joining percentage bootstrap values (black), maximum parsimony bootstrap values (blue) and Bayesian clade credibility values (brown). Nodes also present in the tree generated by Maximum Likelihood are indicated (*). The trees are mid-point rooted. The scale bar corresponds to 0.1 amino acid replacements per site (horizontal axis). Where both mouse and human orthologues are present only the human gene is shown. † There is no mouse ADAM20. Ψ D. rerio ADAM12b and ADAM12c group with ADAM12a (Fig. S3). Ω D.rerio ADAML genes based on location in Fig. S1. The full phylogenetic guide tree is available in Fig. S1. The full phylogenetic trees for the A, B and C subgroups, containing all mouse ADAM orthologues, are presented in Figs. S2-S4. Accession numbers for used in the analyses can be obtained from additional file 1. Further analysis on individual sub-fragments of the B-clade, indicated in Fig. S3, found zebrafish ADAM19b (LOC571252) to group with *H. sapiens *ADAM19 at α; zebrafish ADAM12b (LOC558872) and ADAM12c (LOC561244) to group with *D. rerio *ADAM12A at β and zebrafish ADAM9 (zgc101824) at χ.

**Figure 3:**
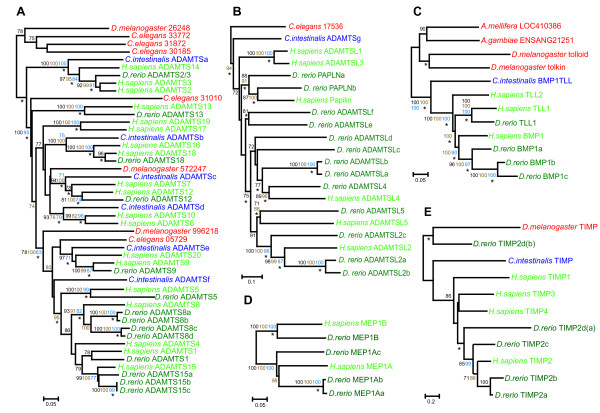
Phylogenetic relationships of metzincin gene families. A. ADAMTS, B. ADAMTS like, C. BMP1/Tolloid-like, D. Meprin and E. TIMP gene families. The trees summarise the phylogenetic analysis. The trees shown were inferred by Neighbor Joining. The values on the tree nodes are Neighbor Joining percentage bootstrap values (black), maximum parsimony bootstrap values (blue) and Bayesian clade credibility values (brown). Nodes also present in the tree generated by Maximum Likelihood are indicated (*). The trees are rooted on the protostome lineage. The scale bars correspond to the number of amino acid replacements per site (horizontal axis). Accession numbers for used in the analyses can be obtained from additional file 1.

**Figure 4:**
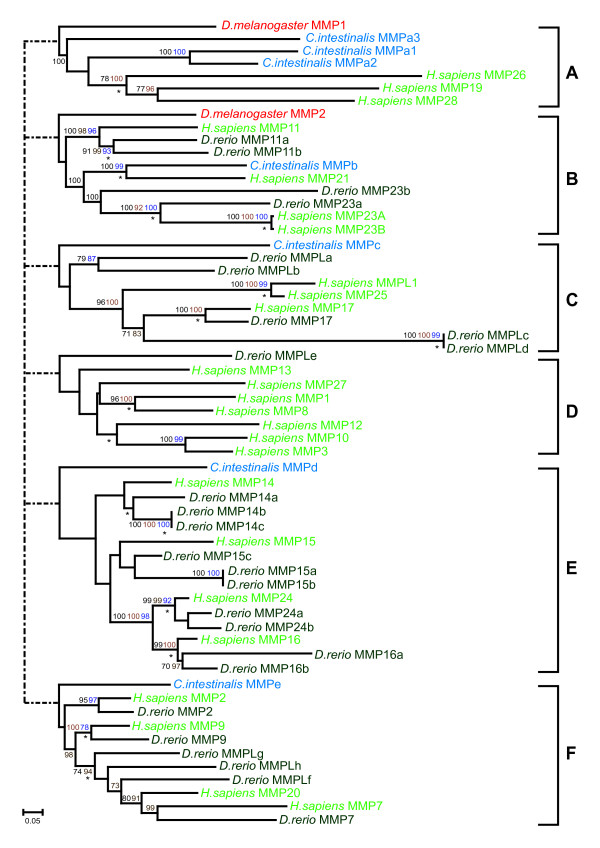
Phylogenetic relationships of the MMP gene family. The MMP gene family was separated into six sub-analyses, indicated A – F, based upon the clades produced and independent phylogenetic analyses performed. The trees shown were inferred by Neighbor Joining from a gapped alignment. The values on the tree nodes are neighbor joining percentage bootstrap values (black), maximum parsimony bootstrap values (blue) and Bayesian clade credibility values (brown). Nodes also present in the tree generated by Maximum Likelihood are indicated (*). The trees are mid-point rooted. The scale bar corresponds to 0.05 amino acid replacements per site (horizontal axis). The MMP gene family was separated into six sub-analyses, indicated A to F, based upon the clades produced and independent phylogenetic analyses performed. The full phylogenetic guide tree is available in Fig. S5. Accession numbers for used in the analyses can be obtained from additional file 1.

The sequences of the specified ciona and zebrafish genes were aligned with the complete set of family members in the human genome. Where appropriate, sequences from other phyla were included (all accession numbers are available in Additional File 1). The relationships of the ciona genes and the zebrafish genes with their human orthologues are shown in Tables 1 and 2 respectively. The detailed phylogenetic relationships of all gene family members studied are depicted in Figures 2, 3, 4.

### ADAM gene family evolution

Due to the fragmented nature of some zebrafish ADAM genes, a gap-stripped phylogenetic analysis could not be performed on the full ADAM family dataset. Therefore, based on an initial Neighbor Joining analysis (Fig. S1, Additional file 4), the ADAM family was divided into three sub-groups (A, B & C) and phylogenetic analyses performed independently on each. The lower levels of sequence divergence within the subgroups allowed for more reliable alignment reconstruction. The dotted lines in Figure 2 show the predicted relationships between the sub-groups based upon the mid-point rooting in the guide tree (Fig. S1, Additional file 4). Furthermore, for the ADAM analysis, it was essential to include mouse orthologues since there are several significant differences between the mouse and human ADAM complement of genes (Fig. 2). Although included in the phylogenetic analyses (Fig. S2-S4 in Additional file 4), for simplicity only mouse genes that are not direct orthologues of human genes are shown in Figure 2. It should therefore be noted that all human genes depicted in Figure 2 (with the sole exception of ADAM20) have a direct mouse orthologue.

The four ADAM genes identified in the ciona genome (Table 1) and the twenty-two ADAM genes in zebrafish (Table 2) clustered into the A and B sub-groups (Fig. 2). The A sub-group consists of two well-defined clades, where drosophila sequences (TACE and kul/kuz respectively) are basal (an outgroup to the clades), followed by single ciona (ADAM a & b respectively) and human sequences (ADAM 17 and 10 respectively), and duplicated zebrafish genes (ADAM 17a & b and ADAM 10a & b respectively). All sequences occurred in the expected relationships based on their animal phyla, class and species (Fig. 2A).

The remaining two ciona (ADAMc1 & c2) and drosophila (Neu3 & mmd) genes form the basal part of the ADAM B-subgroup (Table 1, Figs. 2 and S3). The remainder of the B sub-grouping is composed of an expanded set of vertebrate genes. There are single zebrafish orthologues for five human ADAM genes (ADAM9, ADAM11, ADAM15, ADAM22 and ADAM28), two zebrafish orthologues for ADAM8, ADAM19 and ADAM23, and three zebrafish ADAM12 orthologues (Table 2, Ψ in Fig. 2; Fig. S3). Four zebrafish genes (ADAMLa, b, c & d) clustered weakly in the B clade on the initial phylogenetic analysis (Ω in Fig. 2; Fig. S1), with no statistical support, but appear to be lineage specific in that they have no direct human orthologue (Table 2, Fig. S1). The zebrafish ADAMLb-d gene sequences were relatively short (ADAMLb 449-, ADAMLc 282- and ADAMLd 215-amino acids respectively) and highly divergent and so were excluded from the more detailed B sub-grouping analyses (hence their omission from Fig. 2). In addition, there is no significant statistical support for the positioning of the zebrafish ADAMLa gene shown in Figure 2.

The ADAM C sub-grouping consists entirely of human and mouse sequences. The absence of basal invertebrate or tetrapod orthologues within the ADAM C sub-group infers that this gene expansion occurred on the tetrapod lineage (Fig. 2). However, it is possible that invertebrate orthologues to the ADAM C sub-group have been lost. Further genome sequencing may provide insight into this gene family.

### ADAMTS gene family evolution

We have previously described the characteristics of the six ADAMTS and a single ADAMTS-like (ADAMTSL) gene found in the ciona genome [38]. Their exact relationships with human ADAMTS genes are defined in Table 1 and Figures 3A &3B.

A total of fourteen ADAMTS and thirteen ADAMTS-like (ADAMTSL)/papilin-related genes were identified in the zebrafish genome (Table 2). Single zebrafish orthologues were identified for six human ADAMTS genes (ADAMTS1, ADAMTS5, ADAMTS9, ADAMTS12, ADAMTS13 & ADAMTS18; Table 2, Fig. 3A). In addition, a single zebrafish gene (ADAMTS2/3) was orthologous to human ADAMTS2 and ADAMTS3 (Table 2 and Fig. 3A). Human ADAMTS15 and ADAMTS8 have three and four zebrafish orthologues respectively (Table 2 and Fig. 3A).

A single *C. elegans *and ciona gene roots the ADAMTSL family, which lacks the proteolytic domain that typifies the metzincin superfamily (Table 1 and Fig. 3B). Human ADAMTSL4 and 5 each have a single zebrafish orthologue whereas human papilin and ADAMTSL2 have two and three orthologues respectively (Table 2, Figure 3B). Six zebrafish ADAMTSL genes, ADAMTSLa – ADAMTSLf have no direct human orthologues, however the resolution in this part of the phylogeny is weak (Fig. 3B).

### BMP1/tolloid gene family evolution

We have previously reported that a single ciona gene is orthologous to the three human BMP1/tolloid (TLL) genes (Table 1; [39]). Four zebrafish BMP1/TLL genes were identified in this study (Table 2). Phylogenetic analyses reveal that three of the zebrafish genes (BMP1a, b & c) are orthologous to human BMP1 and the remaining zebrafish gene (TLL1) groups with human TLL1 (Table 2, Fig. 3C).

### Meprin gene family evolution

Ciona does not appear to contain a meprin orthologue whereas four were found in the zebrafish genome (Table 2). Phylogenetic analyses reveal that three of the zebrafish genes are orthologous to human MEP1A and the remaining zebrafish gene is the orthologue of MEP1B (Table 2 and Fig. 3D).

### MMP gene family evolution

Due to the fragmented nature of some zebrafish MMP genes, a gap-stripped phylogenetic analysis could not be performed on the whole dataset. In a similar manner to that of the ADAM genes, the MMP gene family was therefore partitioned into six sub-groups based on an initial Neighbor Joining analysis (Fig. S5, Additional file 4). Each sub-grouping was analysed separately. The dotted lines in Figure 4 represents the individual sub-group positions based on the guide tree (Fig. S5, Additional file 4). However, the relationships among the deep lineages (sub-groups) were not resolved and thus Figure 4 is depicted as an unresolved polytomy.

Seven MMP genes were identified in the ciona genome (Table 1) whereas twenty-six MMPs were found in the zebrafish (Table 2).

The A sub-grouping (Fig. 4) contains three of the ciona genes (MMPa1-3) that are basal to a cluster of related human genes (MMP19, 26 & 28). There are no zebrafish orthologues associated with these genes.

The B sub-grouping consists of three sub-clades where the drosophila MMP2 gene forms an outgroup (Fig. 4). The first sub-clade consists of human MMP11 with duplicated zebrafish genes (MMP11a & b) but no ciona orthologue. The second sub-clade includes human MMP21 and its ciona orthologue (MMPb) and the third contains two zebrafish MMP23(a & b) genes and the very recently duplicated human MMP23(A & B) genes (Fig. 4).

The ciona MMPc gene is basal to the vertebrate genes of the MMP C sub-group. The position of two related zebrafish genes (MMPLa & b) that have no direct human orthologues is not well defined (Fig. 4). The remaining genes appear closely related and include human MMP17, MMP25 and MMPL1, zebrafish MMP17 and a recently duplicated pair of zebrafish paralogues (MMPLc & d).

A single zebrafish gene, MMPLe, clusters with the seven human genes (MMP1, 3, 8, 10, 12, 13 & 27) within the MMP D sub-group although there is no strong support for the positioning of any of the genes apart from the pairing of the human MMPs 1 with 8 and 3 with 10 (Fig. 4).

Ciona MMPd is the basal orthologue to the E sub-grouping of vertebrate MMP genes (Fig. 4). The rest of the grouping contains four human MMP genes, each with multiple zebrafish orthologues. Human MMP16 and 24 have two zebrafish orthologues each whereas both human MMP14 and 15 have three each (Table 2 and Fig. 4).

Ciona MMPe is orthologous to the vertebrate MMP F sub-group. Single zebrafish orthologues were identified for human MMP2, MMP9 and MMP7 in the F sub-grouping (Table 2 and Fig. 4). In addition, the sub-grouping contains three zebrafish genes (MMPLf-h) that do not have direct human orthologues (Table 2 and Fig. 4).

### TIMP gene family evolution

The single TIMP identified in the ciona genome is orthologous to the four human TIMP genes and forms the outgroup to the vertebrate TIMP family (Table 1 and Fig. 3E). Four TIMP genes were identified in the zebrafish genome (Table 2 and Fig. 3E), all of which are orthologues of human TIMP2. One of the zebrafish genes, (TIMP2d), has a duplicated domain structure. The two domains were split up for phylogenetic analysis – TIMP2d(a) and TIMP2d(b) with the C-terminal TIMP2d(b) domain having a sequence that is very divergent in comparison with the other zebrafish sequences (Fig. 3E).

### Mechanisms for metzincin gene family evolution

The 19 metzincin genes identified in the ciona genome (Table 1) cluster into 16 well-supported clades where the ciona gene(s) are orthologous to one or more vertebrate genes. Gene duplications specific to the ciona lineage (ADAMc1/2 and MMPa1/2/3) were exhibited by two of these clades which otherwise contained a single ciona gene. The sixteen clades include a total of fifty-nine human genes. Three of these clades, containing human ADAM10, ADAM17 (Fig. 2) and MMP21 (Fig. 4), had not amplified on the vertebrate lineage and maintained a ciona to human gene ratio of 1:1. The remaining 13 clades contained two or more paralogous human genes, that is, genes that have amplified up from a common progenitor during vertebrate evolution (Fig. 2, 3). In seven (54%) of these 13 clades, two or more duplicated human genes (indicated by * in Table 1) were found to be in paralogous regions of the human genome indicating that the events causing these specific gene amplifications were presumably large scale and less likely to be the result of simple tandem gene duplication alone. Clades where all human members are present in paralogous loci include ADAMTSa, ADAMTSe, ADAMTSf and MMPd (Table 1). An equivalent analysis in zebrafish genes must await the completion of chromosomal locus assignment for genes across the genome.

It is noteworthy that within the ADAM B sub-group there are eight areas of the human genome showing some degree of paralogy to each other. The ADAM B sub-group arose in a complex fashion and appears to have involved both large-scale duplication events and gene loss.

In addition to large-scale duplications suggested by the location of related genes in paralogous regions of the vertebrate (human) genome, tandem duplication has also characterised the metzincin gene expansion in both the tetrapod and teleost lineages. For instance, all the human orthologues in the MMP D sub-group are located on 11q22-24 (Additional File 1) and are rooted by a single zebrafish gene, (MMPLe; Fig. 4) suggesting that the expansion apparent in the human gene repertoire occurred by tandem duplication after divergence of the tetrapod and teleost lineages. The zebrafish MMP14b and MMP14c genes share a very high sequence identity and appear to have evolved from a recent intra-chromosomal duplication of seventeen genes on chromosome two (Fig. S6 in Additional file 4). Recent duplications of other zebrafish metzincins (e.g. ADAMTS15b and c, ADAMTSLa and b, MEP1Aa and b – Fig. 3; and MMP15a and b – Fig. 4) are apparent based on the high degree of sequence identity.

### TIMP gene evolution

The single ciona gene is the orthologue of all four human TIMP genes (Fig. 3E) which, as reported previously, are all located in paralogous regions of the human genome within the intron 5 of paralogous synapsin genes (Table 1 and Additional File 1, [40,41]). This genomic context, which has previously been reported for drosophila and man [42] is conserved in the ciona TIMP and confirmed for one of the zebrafish orthologues, TIMP2d (data not shown).

## Discussion

### Metzincin family evolution

#### 1. ADAM

Members of the ADAM gene family exhibit complex evolutionary relationships (Fig. 2). The two clades within ADAM A sub-group, ADAM10 and ADAM17, both show the expected relationship of genes based on phyla and class, and exhibit duplications within the teleost lineage. ADAM10 and ADAM17 are probably the most characterized of all the ADAM proteases. They are known to play key roles both in activation of EGFR ligands, cross-talk between these ligands and G protein coupled receptors [3] and in early development due to their roles in shedding TGFα [43] and the Notch ligand Delta [44].

It is apparent that much of the ADAM B sub-group evolved from large-scale duplications early in vertebrate evolution, as the majority of human genes have direct zebrafish orthologues, and that subsequent differential gene loss occurred to produce the eight paralogous regions present in the human genome (Fig. 2). Indeed, many of these regions contain tandemly duplicated ADAM genes further complicating the evolutionary history of this family. The extent and nature of the amplification within the ADAM B sub-group suggests that these genes played important roles in the early evolution of vertebrates as opposed to the subsequent divergence of teleost and tetrapods. The fact that a relatively large number of vertebrate members of the ADAM B sub-group have been retained following duplication suggests that these genes have an increased propensity to neo- and/or subfunctionalize. This propensity may be reflection of these proteases function in shedding ectodomains or alternatively, may be indicative of highly adaptable transcriptional control within this sub-group of ADAM genes. The ADAM B sub-group also reveals a tetrapod-specific expansion of five genes (ADAM2, 3, 5, 18 and 32) that play roles in fertility; ADAM2 and 3 are involved in mammalian fertilization [45], ADAM5 is expressed in mouse testis [46], and ADAM32 has been implicated in sperm-egg fusion [47]. The highly specific function of these genes in relation to mammalian biology infers that they evolved more recently during tetrapod evolution. Indeed all of these genes (including the human ADAM3 and ADAM5 pseudogenes) are located at chromosome 8p11.22-23, inferring that this group evolved by tandem duplication and that the pseudogenization of human ADAM3 and ADAM5 [48,49] occurred after the divergence of the rodent and primate lineages.

The ADAM C sub-group contains 4 human and 19 murine genes (Fig. 2). It is apparent that this family has evolved in the tetrapod lineage by both gene loss in the human genome (ADAM1, 3, 4a, 4b, 5, 6 and 25 being pseudogenes – data not shown) and duplications along the rodent lineage. It has previously been hypothesised that the mutations leading to these genes becoming pseudogenes might have contributed to changes in human physiology by disruption of specific processes related to fecundity [50]. Thus evidence that the majority of ADAM C sub-group genes are involved in spermatogenesis and fertilization, are predominantly expressed in the testis [51], and that reproductive genes evolve at a faster rate than other genes [52] could explain the high levels of gene amplification and sequence divergence apparent within this clade (Fig. 2).

#### 2. ADAMTS

We have previously concluded, based on the phylogeny of the human ADAMTS genes, that the majority of the vertebrate ADAMTS family probably evolved from the large-scale duplication events associated with early vertebrate evolution [38]. This conclusion is based on the observation that many of the human ADAMTS genes clustering into clades (Fig. 3A) are found in paralogous regions of the human genome that are the result of genome or large scale duplication events [40]. These same genome duplication events that occurred during early vertebrate evolution are also thought to have caused the expansion in the numbers of genes encoding extracellular matrix proteins [1] – the substrates for the ADAMTS proteases. Thus, genome or large-scale duplications can result in step increases in complexity because of the co-ordinated amplification and subsequent retention of functionally related genes such as growth factors and their receptors or proteases and their substrates. It is therefore surprising that most of the identified ADAMTS clades (Fig. 3A) contain only one zebrafish orthologue since this infers that, after divergence, the teleost lineage has lost most of the duplicated ADAMTS genes that have been retained in the tetrapod lineage. From our data we infer that the teleost (zebrafish) genome contains at least as many extracellular matrix genes as the tetrapod (human) genome. For instance, the teleost lineage actually contains duplicates of many of the fibrillar collagen genes found in land vertebrates [56] and yet the zebrafish has retained only one (ADAMTS2/3) of the three N-proteinases conserved on the tetrapod lineage (Fig. 3A). The only exception is the 'hyalectin-cleaving' ADAMTS clade (rooted by ciona ADAMTSf – Fig. 3A[12]) where zebrafish paralogues of most of human ADAMTS genes are not only retained but, in several cases, duplicated on the fish lineage. Why after divergence, the teleost lineage considerably simplified its repertoire of ADAMTS genes (with the exception of the hyalectin-cleaving proteases) whereas on the tetrapod lineage the genes were retained, is not apparent but, these differences presumably contributed to the evolutionary divergence of the two superclasses.

In contrast to the ADAMTS gene family, the number and phylogenetic relationships of the zebrafish ADAMTS-like genes (that lack the protease domain characteristic of the metzincin superfamily) infers a larger expansion within the teleost (13 genes) than tetrapod lineage (6 genes – Fig. 3B). The fish-specific component of this amplification probably arose by a combination of both genome duplication [39] and subsequent tandem duplications (Fig. 3B).

#### 3. BMP1/TLL

We have previously demonstrated that the vertebrate BMP1/TLL family amplified from a single progenitor gene present in the early chordates [39]. Whilst the zebrafish and human contain relatively similar numbers of BMP1/TLL orthologues (four and three respectively), the distribution of these orthologues is markedly different (Fig. 3C). The phylogeny suggests that the teleost lineage has retained and subsequently amplified a subset of the genes found in the tetrapod lineage (Fig. 3C). It is of interest to note that despite the high level of sequence conservation in substrates for these enzymes including chordin and the fibrillar collagens, the teleost and tetrapod lineages have evolved subtly different expanded repertoires of tolloid isomers.

#### 4. Meprin

No meprin orthologue was identified in the ciona genome. This may have been because this class of metzincin was deleted on the ciona lineage. Indeed, meprin orthologues could not be identified in the *Ciona savignyi *or *Strongylocentrotus purpuratus *(sea urchin, echinoderm) genomes, by BLAST analysis, implying that the meprins are most likely a vertebrate invention. The human (tetrapod) meprin genes appear to have arisen by genome duplication from the early vertebrate progenitor since these genes are located in paralogous regions of the human genome (6p12-p11 and 18q12.2-q12.3 – see additional file 1).

#### 5. MMP

The majority of the complexity that characterises the vertebrate MMP gene family arose during early vertebrate evolution. The vertebrate genes appear to have amplified from five of the seven genes present in the ancestral vertebrate (Fig. 4). Most of the MMP sub-groups show a further expansion along the teleost lineage – wherever a zebrafish orthologue is present, in most cases there are two per human orthologue (Fig. 4). Indeed, this pattern is most apparent within the MMP E sub-group, which exhibits in large part the classical expansions predicted by genome duplication events associated with vertebrate evolution, expanding from a single gene in ciona to four in man and ten (two more than the eight predicted) in zebrafish (Tables 1 and 2). The amplifications along both the vertebrate and subsequent teleost lineages of the type I transmembrane family (MT-MMPs), suggest that the genes are highly retained following duplication, presumably by subfunctionalization into tissue-specific forms, and may have a less tightly constrained functions than other members within the MMP gene sub-family. Within the MMP F sub-group, the presence of an expanded set of teleost orthologues for human MMP 7 & 20 may relate to how teleost teeth are continuously replaced. MMP7 is a matrilysin and MMP20 is an enamelysin, which digests amelogenin, and is present in newly formed tooth enamel [53].

There are relatively few tetrapod specific innovations within the MMP gene family. Although no zebrafish genes are present in the MMP A sub-group, the presence of ancestral invertebrate genes in both *D. melanogaster *and *C. intestinalis *suggests that orthologues were lost on the teleost lineage. In addition, it is likely that MMP26 evolved during the primate lineage as it has only been identified in human, chimpanzee and rhesus monkey genomes. There are two MMP23 genes in both the tetrapod and teleost lineages (MMP B sub-group). The human MMP23A and B have both ShK toxin and immunoglobin-like domains whereas the zebrafish MMP23a and MMP23b do not. A single copy of MMP23 present in rodent genomes also contains the ShK domain. Thus, the phylogenetic analyses coupled with domain structure information infer that the MMP23 gene acquired the ShK domain early during the tetrapod lineage; and that the two copies in man evolved from a recent duplication event that occurred after the rodent-primate divergence. It is of note that ShK domains, potent potassium channel inhibitors, have only been identified in one other family of vertebrate genes, microfibrillar-associated glycoproteins, and are mainly present in sea anemone metridin toxin and several hypothetical proteins in nematodes [54,55]. The most extensive amplification in the tetrapod lineage is apparent in the MMP D sub-group, where the tetrapod members of this sub-group appear to have evolved by tandem duplication (chromosome 11q22) from a gene similar to the zebrafish MMPLe. Many of the tetrapod MMP D sub-group genes are collagenases and as vertebrates have accrued a more extensive and diverse group of collagens (allowing them to develop a wider range of connective tissues such as teeth, skin, cartilage, ligament and bone) [56], one would also expect a more extensive repertoire of collagenases and stromelysins to process and turnover these ECM components.

#### 6. TIMP

The vertebrate TIMP family evolved from a common ancestor present at the start of vertebrate evolution. The distribution of the four human TIMP genes within paralogous regions of the human genome suggests that the family arose from the large-scale duplication events associated with early vertebrate evolution [57]. In a similar manner to the BMP1/TLL gene family, the TIMP genes exhibit different patterns of gene retention between tetrapods (single copies of TIMP1, 2, 3 and 4) and teleosts (four TIMP2) (Fig. 3E). It is possible that following the large-scale duplication events in early vertebrate evolution, some of the four subtypes of TIMP (TIMP1, 3 and 4) were lost along the teleost lineage and that the remaining TIMP2 was co-incidentally or subsequently amplified.

### Metzincin genes in ciona compared to nematodes and insects

The metzincin gene superfamily has ancient metazoan origins, evident in the few protostome orthologues present in these families. It is apparent that a small subset of the metzincin genes, specifically some members of the ADAMTS and MMP clades, have amplified on the deuterostome lineage as the ciona genome contains increased numbers of family members compared to the protostome genomes (Table 3; Fig. 3A and 4). It is also clear that some of the expansions seen in the genomes of extant organisms have arisen from lineage specific duplication events (e.g. *D. melanogaster *ADAM orthologues kul and kuz, ADAM A sub-group Fig. 2; *D. melanogaster *BMP1/TLL orthologues tolkin and tolloid, Fig. 3C; and *C. intestinalis *MMPa1, a2 and a3, MMP A sub-group, Fig. 4). Nevertheless, taking an overview of the phylogenetic data presented, it is most striking how similar the ciona complement of metzincin genes is to that of the protostomes (Figs 2, 3, 4; Table 3), suggesting that the metzincin gene complement has remained relatively stable throughout the evolution of both the protostome lineage, and the deuterostome lineage up to and including early chordates. This is in accord with the relative extracellular matrix gene content of these two lineages, which is also highly comparable between protostomes and early chordates [39].

**Table 3:** Core metazoan metzincins and deuterostome innovations.

**Gene family**	Ancestral protostome*	**Ciona**	**Human**	**Zebrafish**
ADAM	3	3	21	22	
ADAMTS	5	7	25	27
BMP1/TLL	1	1	3	4
Meprin	0	0	2	4
MMP	2	5	25	26
TIMP	1	1	4	4

The number of genes present in the ancestral protostomes and chordate lineages are displayed for their respective gene family. * Ancestral Protostome content is the representative gene content based on *Drosophila melanogaster*, *Apis mellifera*, *Anopheles gambiae *and *Caenorhabditis elegans *genomes.

### Metzincin genes in human compared to ciona

Apart from a rodent-specific amplification in the ADAM clade, human and mouse complements of metzincin genes are very similar. Therefore, for the purpose of this study, the human complement of metzincins was used as a representative of the generic complement in the tetrapod lineage. Most of the families of metzincin genes found in vertebrates are represented in ciona (Table 1) and therefore evolved before the divergence of the urochordate and vertebrate lineages (Table 1). There is a single instance of an apparent *de novo *creation of a vertebrate metzincin family, the meprins, although it is also possible that the meprins evolved earlier in chordate evolution but the orthologue was subsequently ablated in ciona. However, the major evolutionary change in the metzincin gene repertoire of vertebrates in comparison with ciona is the widespread duplication of the majority of pre-existing genes (Table 1). Indeed, the nineteen ciona metzincins fell into sixteen clades where the majority of vertebrate genes had duplicated to produce two to six paralogues per clade (Tables 1, 3 and Figs. 2, 3, 4). Metzincin genes are distributed throughout the human genome. Some of the duplications in the vertebrate metzincin gene families have arisen from tandem duplication events. However, many of the human metzincin genes are distributed within multiple paralogons suggesting that large-scale genomic or genome duplication events must have played a significant role in the generation of these vertebrate metzincin genes. Therefore, it appears most likely that the majority of the complexity apparent in the vertebrate (tetrapod) metzincin compliment arose during early vertebrate evolution by duplication of pre-existing genes.

The most comprehensive mechanism of producing widespread and co-ordinated gene duplication is genome duplication and increasing evidence supports the notion that the at least one, if not two, rounds of genome duplication occurred during early vertebrate evolution [57,58]. The phylogenies within the metzincin superfamily support this contention, where the majority of human duplicates are in paralogous regions of the genome (ADAMTS, ADAM, Meprin, MMP and also TIMP). Thus, the relationship between ciona and tetrapod (human) metzincin genes shows, in general, an amplification in gene number indicative of the one to two rounds of genome duplication associated with early vertebrate evolution [57].

### Metzincin genes in teleosts compared to tetrapods

The identification of metzincin orthologues in the teleost *Danio rerio *has not only inferred multiple duplications specific to the teleost lineage, but has also highlighted tetrapod-specific innovations in the metzincin gene families. Taking an overview of the phylogenetic data, a majority of the zebrafish metzincin genes occur in pairs (see Figs. 2, 3, 4). It should be noted, however, that some of these pairs of fish genes are almost identical in sequence indicating that they have arisen very recently, probably by tandem gene duplication. An increased number of metzincin paralogues in the zebrafish compared to human genome is what would be predicted if a teleost-specific genome duplication had occurred after their divergence from the tetrapod lineage [59].

Our data also highlights different ways in which teleosts and tetrapods have evolved to perform similar functions. For instance, fish and tetrapod lineages share highly conserved fibrillar collagens requiring N- and C-terminal processing during assembly. The C-proteinase BMP1/TLL family members in fish and man are related but the fish gene complement has arisen by fish-specific amplifications and the deletion of paralogues conserved in tetrapods (Fig. 3C). Similarly, the zebrafish appears to have ablated two of the three highly specific N-proteinase genes found in man (ADAMTS2, ADAMTS3 and ADAMTS14; Fig. 3A), which are believed to have evolved prior to the divergence of the teleost and tetrapod lineages due to their paralogous locations within the human genome.

It is surprising that the fish genome seems to have refined its metzincin repertoire to a much greater extent than tetrapods. We contend that the occurrence of so many tetrapod metzincin homologues in paralogous genomic loci is a reasonable indicator that most of these duplications resulted from the genome events characterising early vertebrate evolution. Subsequently, the teleost and tetrapod lineages must have shared this metzincin repertoire at their divergence. However, in a number of cases, the teleost lineage has subsequently ablated a significant number of paralogous genes that have been retained on the tetrapod lineage (e.g see ADAMTS clade; Fig. 3A). It is possible that these differences in gene retention patterns were facilitated by a teleost-specific genome duplication event [37]. It is of interest to note that a recent large-scale analysis of the zebrafish genome postulated that the genome duplication events associated with early vertebrate evolution characterized the fish to a much greater extent than the later teleost specific large-scale duplication [60]. However, it may be that this extra genome duplication provided the teleost genome with a degree of plasticity, seen by differential gene loss within the phyla, that could account for the increase in speciation from a few dozen ray-finned fishes to over 25,000 teleosts [61,62]. The relative duplication and recombination driven plasticity exhibited within the teleost genomes [62,63], leads us to speculate that it may be necessary to combine the results from multiple teleost genomes (such as *Danio rerio*, *Oryzias latipes*, *Takifugu rubripes*, *Tetradon nigroviridis *and most recently the stickleback *Gasterosteus aculeatus*) to achieve a view of a truly representative teleost genome for comparative purposes. For instance, comparison of the zebrafish and Tetradon/Takifugu genomes [64,65] revealed that despite the high degree of synteny and the retention of similar numbers of gene duplicates, in a significant number of cases, different paralogues have been retained. Indeed, a study on the ADAMTS complement of *Takifugu rubripes *reveals a similar number of genes compared to the zebrafish reported here (16 versus 14 per genome respectively) [66]. However, the pattern of gene duplication, retention and loss is markedly different in the two species.

## Conclusion

The complexity seen in the vertebrate metzincin gene families was mainly acquired during vertebrate evolution through the duplication of pre-existing genes rather than through *de novo *gene innovation. Prior to the emergence of vertebrates, the metzincin gene repertoire in protostomes and invertebrate deuterostomes remained relatively stable. The metzincin gene repertoire of extant tetrapods, such as man, has resulted largely from duplication events associated with early vertebrate evolution. The teleost repertoire of metzincin genes in part parallels that of tetrapods but has been significantly modified, perhaps as a consequence of a teleost-specific duplication event.

The analyses described above provide the most likely explanation for how the complexity of the metzincin gene superfamily has arisen. This represents the first step in determining the functional significance of the subtly different patterns of gene retention in different vertebrate lineages that will provide new insights into events that enabled and underpinned the evolution of vertebrates and the different classes and species therein.

## Methods

### Sequence identification

To identify homologous genes, the complete sequences of the human metalloprotease genes were used to probe the genome and TIGR gene index of *C. intestinalis *and the genome of *Danio rerio *using TBLASTN and PSI-BLAST with cut-off expectancy values of *E *= 1 [33,67-69]. Resources used are available at JGI, TIGR and NCBI [70-72]. Ciona gene models were also detected using the orthologue detection program InParanoid by keyword searches using ECM gene family names as queries (e.g. 'MMP) [73,74]. To identify as many metalloprotease genes as possible, reciprocal BLAST searches of the ciona, human, zebrafish and non-redundant databases were performed. In addition, the mouse genome was searched in the case of the ADAM gene family.

Frequently, EST data contradicted the ciona gene model coding sequence proposed by JGI. In instances where an EST clearly demonstrated the misplacement of exons in the recovered JGI model, the protein sequence was corrected to reflect this. Through comparison with recovered ESTs and by searching flanking genomic DNA using GENEWISE and SignalP [75,76], erroneous and missing regions of the gene models were corrected. Modified sequences were checked by aligning with respective human ECM genes using CLUSTALX [77] and corrected coding sequences (presented in the annex to Supplementary Table 1) used for subsequent analyses. Zebrafish gene models were checked in a similar manner using the NCBI and Ensembl [72,78] websites using comparisons against both the human and ciona predicted gene models.

### Phylogenetic analyses

Phylogenetic analyses were performed on the metalloprotease genes for each gene family. The accession numbers for protein sequences used in these studies are presented in Supplementary Table S1. The ciona and zebrafish genes identified were aligned with each gene family using CLUSTALX [77]. A preliminary bootstrapped Neighbor-joining tree was drawn using CLUSTALX and the sequences were then divided into sub-groups based on their position in the tree. For each sub-group, new multiple alignments were created, gap-containing sites were removed and four independent phylogenetic methods were performed. Neighbor joining trees and bootstrap replicates were generated using SEQBOOT, PROTDIST, NEIGHBOR and CONSENSE from the PHYLIP package using the default settings [79]. Maximum Parsimony trees and bootstrap replicates were obtained using SEQBOOT, PROTPARS and CONSENSE and Maximum Likelihood trees were inferred using PROML from the PHYLIP package using the default settings [79]. The JTT model of amino acid substitutions was used with global rearrangements and correction for rate heterogeneity (α value obtained from TREEPUZZLE [80]). Bayesian tree inference values were produced from the MrBayes programme [81] where Markov Chain Monte Carlo analysis was performed for 100,000 generations using 6 chains.

## List of Abbreviations

ADAM A disintegrin and metalloprotease domain

ADAMTS A disintegrin and metalloprotease domain with thrombospondin type 1 motif

BMP Bone morphogenetic protein

MMP Matrix metalloprotease

TIMP Tissue inhibitors of metalloprotease

TLL Tolloid like

## Authors' contributions

DLR and RBH conceived the study. JHJ, T-KC, CB and GT carried out the BLAST and phylogenetic analysis. JHJ, DLR and RBH wrote the manuscript and all authors read and approved the final manuscript.

## Supplementary Material

Additional file 1 Accession information for all metzincin genes used in the analysis. Accession numbers, gene names and locus information for all genes used in the study Click here for file

Additional file 2 Ciona sequences corrected from the original JGI gene models Click here for file

Additional file 3 Zebrafish sequences used in the analysis Click here for file

Additional file 4 Supplementary figures S1-S6. Initial guide-tree phylogenetic analyses of the ADAM and MMP gene families, full sub-group analyses of the ADAM gene family and diagrammatic representation of a tandem duplication located on the *Danio rerio *chromosome 2 Click here for file
