# Supplementary material for: The evolution of the vertebrate metzincins; insights from Ciona intestinalis and Danio rerio
Source: BMC Evol Biol. 2007 Apr 17;7:63. doi: 10.1186/1471-2148-7-63 (PMC1867822; doi:10.1186/1471-2148-7-63)
Supplement: Additional file 1 — Accession information for all metzincin genes used in the analysis. Accession numbers, gene names and locus information for all genes used in the study [file 1471-2148-7-63-S1.doc]

**Additional file 1**

**Accession information for all metzincin genes used in the analysis.**

Ciona sequences that have been modified from the original JGI accession are indicated and can be sourced from ^a^ sequence store below and ^b^ Huxley-Jones et al., 2005. All zebrafish sequences used in the analysis can be sourced from Supplementary Sequence File.

| **Species** | **Data** | **Accession** | **Gene ID** | **Name Used** | **Locus** |
| --- | --- | --- | --- | --- | --- |
| **ADAM** | | | | | |
| D. melanogaster | NCBI | NP_733334 | TACE | TACE | 3R 99D1-99D1 |
| D. melanogaster | NCBI | NP_651716 | kul | kul | 3R 99B9-99B9 |
| D. melanogaster | NCBI | NP_477187 | kuz | kuz | 2L 34C4-34C6 |
| D. melanogaster | NCBI | NP_523358 | mmd | mmd | X 14A1-14A3 |
| D. melanogaster | NCBI | NP_731935 | Neu3 | Neu3 | 3R 88C11-88C11 |
| C. intestinalis | JGI | ci0100152455 ^a^ | ci152455 | ADAMa | chr_02q:4937279-4945949 |
| C. intestinalis | JGI | ci0100139599 ^a^ | ci139599 | ADAMb | chr_03q:771941-780444 |
| C. intestinalis | JGI | ci0100131886^a^ | ci131886 | ADAMc1 | scaffold_171:97617-107221 |
| C. intestinalis | JGI | ci0100140827 ^a^ | ci140827 | ADAMc2 | chr_12p:381811-388419 |
| M. musculus | NCBI | NP_742124 | ADAM1A | ADAM1Am | 5 |
| M. musculus | NCBI | NP_742123 | ADAM1B | ADAM1Bm | 5 |
| H. sapiens | NCBI | NP_001455 | ADAM2 | ADAM2 | 8p11.2 |
| M. musculus | NCBI | NP_033748 | ADAM2 | ADAM2m | 14 28.0 |
| M. musculus | NCBI | NP_033749 | ADAM3 | ADAM3m | 16q12.1 |
| M. musculus | NCBI | NP_033750 | ADAM4 | ADAM4m | 12 |
| M. musculus | NCBI | XP_146967 | ADAM4b | ADAM4bm | 12D1 |
| M. musculus | NCBI | NP_031427 | ADAM5 | ADAM5m | 8 |
| M. musculus | NCBI | NP_777479 | ADAM6 | ADAM6m | 12 |
| M. musculus | NCBI | NP_001009545 | ADAM6b | ADAM6bm | 12F1 |
| H. sapiens | NCBI | NP_003808 | ADAM7 | ADAM7 | 8p21.2 |
| M. musculus | NCBI | NP_031428 | ADAM7 | ADAM7m | 14 28.7 |
| H. sapiens | NCBI | NP_001100 | ADAM8 | ADAM8 | 10q26.3 |
| M. musculus | NCBI | NP_031429 | ADAM8 | ADAM8m | 7 F3-F5 |
| H. sapiens | NCBI | NP_003807 | ADAM9 | ADAM9 | 8p11.23 |
| M. musculus | NCBI | NP_031430 | ADAM9 | ADAM9m | 8 8.0 |
| H. sapiens | NCBI | NP_001101 | ADAM10 | ADAM10 | 15q22 |
| M. musculus | NCBI | NP_031425 | ADAM10 | ADAM10m | 9 41.0 |
| H. sapiens | NCBI | NP_002381 | ADAM11 | ADAM11 | 17q21.3 |
| M. musculus | NCBI | NP_033743 | ADAM11 | ADAM11m | 11 60.0 |
| H. sapiens | NCBI | NP_003465 | ADAM12 | ADAM12 | 10q26.3 |
| M. musculus | NCBI | NP_031426 | ADAM12 | ADAM12m | 7 |
| H. sapiens | NCBI | NP_997080 | ADAM15 | ADAM15 | 1q21.3 |
| M. musculus | NCBI | NP_033744 | ADAM15 | ADAM15m | 3 44.4 |
| H. sapiens | NCBI | NP_003174 | ADAM17 | ADAM17 | 2p25 |
| M. musculus | NCBI | NP_033745 | ADAM17 | ADAM17m | 12 3.0 |
| H. sapiens | NCBI | NP_055052 | ADAM18 | ADAM18 | 8p11.22 |
| M. musculus | NCBI | NP_034214 | ADAM18 | ADAM18m | 8 A3 |
| H. sapiens | NCBI | NP_075525 | ADAM19 | ADAM19 | 5q32-q33 |
| M. musculus | NCBI | NP_033746 | ADAM19 | ADAM19m | 11 20.0 |
| H. sapiens | NCBI | NP_003805 | ADAM20 | ADAM20 | 14q24.1 |
| H. sapiens | NCBI | NP_003804 | ADAM21 | ADAM21 | 14q24.1 |
| M. musculus | NCBI | NP_065063 | ADAM21 | ADAM21m | 12 28.0 |
| H. sapiens | NCBI | NP_068369 | ADAM22 | ADAM22 | 7q21 |
| M. musculus | NCBI | NP_001007221 | ADAM22 | ADAM22m | 5 |
| H. sapiens | NCBI | NP_003803 | ADAM23 | ADAM23 | 2q33 |
| M. musculus | NCBI | NP_035910 | ADAM23 | ADAM23m | 1 31.5 |
| M. musculus | NCBI | NP_034216 | ADAM24 | ADAM24m | 8 |
| M. musculus | NCBI | NP_035911 | ADAM25 | ADAM25m | 8 |
| M. musculus | NCBI | NP_034215 | ADAM26 | ADAM26m | 8 |
| M. musculus | NCBI | NP_001009547 | ADAM26B | ADAM26bm | 8 |
| H. sapiens | NCBI | NP_055080 | ADAM28 | ADAM28 | 8p21.2 |
| M. musculus | NCBI | NP_034212 | ADAM28 | ADAM28m | 14 D1 |
| H. sapiens | NCBI | NP_055084 | ADAM29 | ADAM29 | 4q34 |
| M. musculus | NCBI | NP_787953 | ADAM29 | ADAM29m | 8 B1.3 |
| H. sapiens | NCBI | NP_068566 | ADAM30 | ADAM30 | 1p13-p11 |
| M. musculus | NCBI | NP_081941 | ADAM30 | ADAM30m | 3 F3 |
| H. sapiens | NCBI | NP_659441 | ADAM32 | ADAM32 | 8p11.23 |
| M. musculus | NCBI | NP_700446 | ADAM32 | ADAM32m | 8 A2 |
| H. sapiens | NCBI | NP_079496 | ADAM33 | ADAM33 | 20p13 |
| M. musculus | NCBI | NP_291093 | ADAM33 | ADAM33m | 2 73.9 |
| M. musculus | NCBI | NP_665688 | ADAM34 | ADAM34m | 8 |
| M. musculus | NCBI | NP_001020411 | ADAM36 | ADAM36m | 8 |
| M. musculus | NCBI | XP_357876 | ADAM37 | ADAM37m | 8 |
| M. musculus | NCBI | NP_001009548 | ADAM38 | ADAM38m | 8 |
| M. musculus | NCBI | NP_001020551 | ADAM39 | ADAM39m | 8 |
| M. musculus | NCBI | XP_356072 | ADAM40 | ADAM40m | 8 |
| H. sapiens | NCBI | NP_055294 | ADAMDEC1 | ADAMDEC1 | 8p21.2 |
| M. musculus | NCBI | NP_067450 | ADAMDEC1 | ADAMDEC1m | 14d |
| D. rerio | NCBI | AAH57428 | adam8 | ADAM8a | 13 |
| D. rerio | NCBI | XP_684931 | LOC556905 | ADAM8b | 12 |
| D. rerio | NCBI | NP_001004678 | zgc101824 | ADAM9 | 8 |
| D. rerio | NCBI | XP_694405 | LOC566044 | ADAM10a | un |
| D. rerio | NCBI | BC054638 | zgc64203 | ADAM10b | un |
| D. rerio | NCBI | XP_695529 | LOC567145 | ADAM11 | 12 |
| D. rerio | NCBI | XP_691731 | LOC563275 | ADAM12a | 17 |
| D. rerio | NCBI | XP_687238 | LOC558872 | ADAM12b | 17 |
| D. rerio | NCBI | XP_689742 | LOC561244 | ADAM12c | 7 |
| D. rerio | NCBI | XP_683689 | LOC555937 | ADAM15 | 3 |
| D. rerio | NCBI | NP_955967 | zgc63886 | ADAM17a | 20 |
| D. rerio | NCBI | XP_689147 | sidkey81b152 | ADAM17b | 20 |
| D. rerio | NCBI | XP_693712 | LOC565342 | ADAM19a | 7 |
| D. rerio | NCBI | XP_699915 | LOC571252 | ADAM19b | un |
| D. rerio | NCBI | XP_695103 | LOC566725 | ADAM22 | 9 |
| D. rerio | NCBI | XP_691619 | LOC563160 | ADAM23a | 6 |
| D. rerio | NCBI | XP_693521 | LOC565131 | ADAM23b | un |
| D. rerio | NCBI | XP_686264 | LOC558008 | ADAM28 | 5 |
| D. rerio | NCBI | XP_699214 | LOC570620 | ADAMLa | un |
| D. rerio | NCBI | XP_696794 | LOC568376 | ADAMLb | 22 |
| D. rerio | NCBI | XP_689465 | LOC560975 | ADAMLc | un |
| D. rerio | NCBI | XP_699253 | LOC570656 | ADAMLd | un |
| **ADAMTS** | | | | | |
| D. melanogaster | NCBI | NP_572247 | 572247 | CG4096 | X 5B1 |
| D. melanogaster | NCBI | NP_726248 | 26248 | CG3622 | 2R 59B2 |
| D. melanogaster | NCBI | NP_996218 | 996218 | CG14869 | 3R 88F5-F6 |
| C. elegans | NCBI | NP_501792 | 5729 | gon-1 | IV |
| C. elegans | NCBI | NP_510116 | 31872 | adt-1 | X |
| C. elegans | NCBI | CE07514 | 30185 | CE07514 | un |
| C. elegans | NCBI | NP_001024532 | 33772 | adt-2 | X |
| C. elegans | NCBI | NP_505901 | 31010 | mig-17 | V |
| C. elegans | NCBI | CE17536 | 17536 | CE30735 | un |
| C. intestinalis | JGI | ci0100146117 ^b^ | Ciona1044 | ADAMTSa | chr_09p:103624-116678 |
| C. intestinalis | JGI | ci0100138085 ^b^ | Ciona25 | ADAMTSb | chr_03q:832740-848328 |
| C. intestinalis | JGI | ci0100132574 ^b^ | Ciona34 | ADAMTSc | chr_04q:619670-635292 |
| C. intestinalis | JGI | ci0100146470 ^b^ | Ciona91 | ADAMTSd | chr_08q:4312191-4324398 |
| C. intestinalis | JGI | ci0100132719 | Ciona677 | ADAMTSe | chr_01q:1161784-1198671 |
| C. intestinalis | JGI | ci0100137065 | Ciona250 | ADAMTSf | scffold_250:64161-71880 |
| C. intestinalis | JGI | ci0100130847 ^b^ | Ciona479 | ADAMTSg | scaffold_62:45209-64284 |
| H. sapiens | NCBI | NP_008919 | ADAMTS1 | ADAMTS1 | 21q21.2 |
| H. sapiens | NCBI | NP_055059 | ADAMTS2 | ADAMTS2 | 5qter |
| H. sapiens | NCBI | NP_055058 | ADAMTS3 | ADAMTS3 | 4q21.1 |
| H. sapiens | NCBI | NP_005090 | ADAMTS4 | ADAMTS4 | 1q21-q23 |
| H. sapiens | NCBI | NP_008969 | ADAMTS5 | ADAMTS5 | 21q21.3 |
| H. sapiens | NCBI | NP_055088 | ADAMTS6 | ADAMTS6 | 5pter-qter |
| H. sapiens | NCBI | NP_055087 | ADAMTS7 | ADAMTS7 | 15q24.2 |
| H. sapiens | NCBI | NP_008968 | ADAMTS8 | ADAMTS8 | 11q25 |
| H. sapiens | NCBI | NP_891550.1 | ADAMTS9 | ADAMTS9 | 3p14.3-p14.2 |
| H. sapiens | NCBI | NP_112219 | ADAMTS10 | ADAMTS10 | 19p13.1 |
| H. sapiens | NCBI | NP_002381 | ADAMTS12 | ADAMTS12 | 5q35 |
| H. sapiens | NCBI | NP_620594 | ADAMTS13 | ADAMTS13 | 9q34 |
| H. sapiens | NCBI | NP_542453 | ADAMTS14 | ADAMTS14 | 10q2 |
| H. sapiens | NCBI | NP_620686 | ADAMTS15 | ADAMTS15 | 11q25 |
| H. sapiens | NCBI | NP_620687 | ADAMTS16 | ADAMTS16 | 5p35 |
| H. sapiens | NCBI | NP_620688 | ADAMTS17 | ADAMTS17 | 15q24 |
| H. sapiens | NCBI | NP_955387 | ADAMTS18 | ADAMTS18 | 16q34 |
| H. sapiens | NCBI | NP_598377 | ADAMTS19 | ADAMTS19 | 5q31 |
| H. sapiens | NCBI | NP_787047 | ADAMTS20 | ADAMTS20 | 12q12 |
| H. sapiens | NCBI | NP_640329 | ADAMTSL1 | ADAMTSL1 | 9p22.1 |
| H. sapiens | NCBI | NP_055509 | ADAMTSL2 | ADAMTSL2 | 9q34.2 |
| H. sapiens | NCBI | NP_997400 | ADAMTSL3 | ADAMTSL3 | 15q25 |
| H. sapiens | NCBI | NP_061905 | ADAMTSL4 | ADAMTSL4 | 1q21.2 |
| H. sapiens | NCBI | NP_998769 | ADAMTSL5 | ADAMTSL5 | 19p13.3 |
| H. sapiens | NCBI | NP_775733 | Papilin | Papilin | 14q24.2 |
| D. rerio | NCBI | XP_693535 | LOC565145 | ADAMTS1 | un |
| D. rerio | NCBI | XP_700384 | LOC571682 | ADAMTS2/3 | un |
| D. rerio | NCBI | XP_693454 | LOC565053 | ADAMTS5 | un |
| D. rerio | NCBI | XP_697476 | LOC569024 | ADAMTS8a | 5 |
| D. rerio | NCBI | XP_696612 | LOC568205 | ADAMTS8b | 15 |
| D. rerio | NCBI | XP_693854 | LOC565478 | ADAMTS8c | 15 |
| D. rerio | NCBI | XP_698107 | LOC569618 | ADAMTS8d | un |
| D. rerio | NCBI | XP_694489 | LOC566130 | ADAMTS9 | 11 |
| D. rerio | NCBI | XP_687070 | LOC558722 | ADAMTS12 | 10 |
| D. rerio | NCBI | XP_699415 | LOC570801 | ADAMTS13 | un |
| D. rerio | NCBI | XP_693920 | LOC565549 | ADAMTS15a | 5 |
| D. rerio | NCBI | XP_696556 | LOC568151 | ADAMTS15b | 15 |
| D. rerio | NCBI | XP_698060 | LOC569571 | ADAMTS15c | un |
| D. rerio | NCBI | XP_683701 | LOC559948 | ADAMTS18 | 7 |
| D. rerio | NCBI | XP_689085 | LOC560595 | ADAMTSL2a | 5 |
| D. rerio | NCBI | XP_689866 | LOC561364 | ADAMTSL2b | 5 |
| D. rerio | NCBI | XP_696251 | LOC567853 | ADAMTSL2c | un |
| D. rerio | NCBI | XP_685637 | LOC557465 | ADAMTSL4 | 14 |
| D. rerio | NCBI | XP_687256 | LOC558887 | ADAMTSL5 | 22 |
| D. rerio | NCBI | XP_687414 | LOC559025 | PAPLNa | 22 |
| D. rerio | NCBI | NP_001018400 | ZGC_110061 | PAPLNb | 20 |
| D. rerio | NCBI | XP_684135 | LOC556279 | ADAMTSLa | 1 |
| D. rerio | NCBI | XP_684575 | LOC556634 | ADAMTSLb | 1 |
| D. rerio | NCBI | XP_692137 | LOC563685 | ADAMTSLc | 8 |
| D. rerio | NCBI | XP_697239 | LOC568792 | ADAMTSLd | 12 |
| D. rerio | NCBI | XP_699153 | LOC570564 | ADAMTSLe | un |
| D. rerio | NCBI | XP_701280 | LOC572470N | ADAMTSLf | un |
| **BMP1/Tolloid-like** | | | | | |
| A. gambiae | NCBI | XP_313494 | ENSANG21251 | ENSANGG00000018762 | 2R |
| A. mellifera | NCBI | XP_393866 | LOC410386 | LOC410386 | LG12 |
| D. melanogaster | NCBI | NP_524487 | tolloid | tolloid | 3R 96A19 |
| D. melanogaster | NCBI | NP_733035 | tolkin | tolkin | 3R 96A18-A19 |
| C. intestinalis | JGI | ci0100139893 | ci139893 | BMP1/TLL | chr_12q:2334622-2337772 |
| H. sapiens | NCBI | NP_006120 | BMP-1 | BMP-1 | 8p21 |
| H. sapiens | NCBI | NP_036596 | TLL-1 | Tolloid-like 1 | 4q32-q33 |
| H. sapiens | NCBI | NP_036597 | TLL-2 | Tolloid-like 2 | 10q23-q24 |
| D. rerio | NCBI | NP_001035126 | BMP1A | BMP1a | un |
| D. rerio | NCBI | NP_001034901 | BMP1B | BMP1b | 9 |
| D. rerio | NCBI | XP_686827 | LOC558505 | BMP1c | 1 |
| D. rerio | NCBI | NP_571085 | TLL1 | TLL1 | 1 |
| **Meprin** | | | | | |
| H. sapiens | NCBI | NP_005579 | MEP1A | Meprin A alpha | 6p12-p11 |
| H. sapiens | NCBI | NP_005916 | MEP1B | Meprin A beta | 18q12.2-q12.3 |
| D. rerio | NCBI | NP_001025452 | MEP1A | MEP1Aa | 20 |
| D. rerio | NCBI | XP_695419 | LOC567040 | MEP1Ab | 20 |
| D. rerio | NCBI | XP_693907 | DKEY-30J22.6 | MEP1Ac | 20 |
| D. rerio | NCBI | XP_698972 | CH211-191A24.6 | MEP1B | 20 |
| **MMP** | | | | | |
| D. melanogaster | NCBI | NP_726473 | MMP1 | MMP1 | 2R 60D13-D14 |
| D. melanogaster | NCBI | NP_610511 | MMP2 | MMP2 | 2R 45F6-46A1 |
| C. intesintalis | JGI | ci0100143853 ^a^ | ci143853 | MMPa1 | chr_01q:1219308-1223787 |
| C. intesintalis | JGI | ci0100149359 ^a^ | ci149359 | MMPa2 | chr_01q:5048503-5055511 |
| C. intesintalis | JGI | ci0100148688 ^a^ | ci148688 | MMPa3 | chr_09q:1817771-1828878 |
| C. intesintalis | JGI | ci0100138953 ^a^ | ci138953 | MMPb | chr_05q:4620391-4626957 |
| C. intesintalis | JGI | ci0100146960 ^a^ | ci146960 | MMPc | chr_04q:4926440-4932395 |
| C. intesintalis | JGI | ci0100152154 ^a^ | ci152154 | MMPd | chr_01q:569060-585717 |
| C. intesintalis | JGI | ci0100151527 ^a^ | ci151527 | MMPe | chr_03q:362616-371946 |
| H. sapiens | NCBI | NP_002412 | MMP1 | MMP1 | 11q22.3 |
| H. sapiens | NCBI | NP_004521 | MMP2 | MMP2 | 16q13-q21 |
| H. sapiens | NCBI | NP_002413 | MMP3 | MMP3 | 11q22.3 |
| H. sapiens | NCBI | NP_002414 | MMP7 | MMP7 | 11q21-q22 |
| H. sapiens | NCBI | NP_002415 | MMP8 | MMP8 | 11q22.3 |
| H. sapiens | NCBI | NP_004985 | MMP9 | MMP9 | 20q11.2-q13.1 |
| H. sapiens | NCBI | NP_002416 | MMP10 | MMP10 | 11q22.3 |
| H. sapiens | NCBI | NP_005931 | MMP11 | MMP11 | 22q11.23 |
| H. sapiens | NCBI | NP_002417 | MMP12 | MMP12 | 11q22.3 |
| H. sapiens | NCBI | NP_002418 | MMP13 | MMP13 | 11q22.3 |
| H. sapiens | NCBI | NP_004986 | MMP14 | MMP14 | 14q11-q12 |
| H. sapiens | NCBI | NP_002419 | MMP15 | MMP15 | 16q13-q21 |
| H. sapiens | NCBI | NP_005932 | MMP16 | MMP16 | 8q21 |
| H. sapiens | NCBI | NP_057239 | MMP17 | MMP17 | 12q24.3 |
| H. sapiens | NCBI | NP_002420 | MMP19 | MMP19 | 12q14 |
| H. sapiens | NCBI | NP_004762 | MMP20 | MMP20 | 11q22.3 |
| H. sapiens | NCBI | NP_671724 | MMP21 | MMP21 | 10q26.2 |
| H. sapiens | NCBI | NP_004650 | MMP23A | MMP23A | 1p36.3 |
| H. sapiens | NCBI | NP_008914 | MMP23B | MMP23B | 1p36.3 |
| H. sapiens | NCBI | NP_006681 | MMP24 | MMP24 | 20q11.2 |
| H. sapiens | NCBI | NP_071913 | MMP25 | MMP25 | 16p13.3 |
| H. sapiens | NCBI | NP_068573 | MMP26 | MMP26 | 11p15 |
| H. sapiens | NCBI | NP_071405 | MMP27 | MMP27 | 11q24 |
| H. sapiens | NCBI | NP_077278 | MMP28 | MMP28 | 17q11-q21.1 |
| H. sapiens | NCBI | NP_004133 | MMPL1 | MMPL1 | 16p13.3 |
| D. rerio | NCBI | NP_932333 | MMP2 | MMP2 | 7 |
| D. rerio | NCBI | XP_698683 | LOC570150 | MMP7 | un |
| D. rerio | NCBI | NP_998288 | MMP9 | MMP9 | 8 |
| D. rerio | NCBI | XP_692205 | LOC563753 | MMP11a | 9 |
| D. rerio | NCBI | XP_694153 | LOC565793 | MMP11b | 21 |
| D. rerio | NCBI | NP_919397 | MMP14a | MMP14a | 24 |
| D. rerio | NCBI | NP_919395 | MMP14b | MMP14b | 2 |
| D. rerio | NCBI | XP_690227 | LOC566945 | MMP14c | 2 |
| D. rerio | NCBI | XP_689536 | LOC561041 | MMP15a | un |
| D. rerio | NCBI | XP_690576 | LOC562086 | MMP15b | un |
| D. rerio | NCBI | XP_690769 | LOC562281 | MMP15c | 7 |
| D. rerio | NCBI | XP_700663 | LOC571926 | MMP16a | un |
| D. rerio | NCBI | XP_700781 | LOC572034 | MMP16b | un |
| D. rerio | NCBI | XP_698601 | LOC570076 | MMP17 | 14 |
| D. rerio | NCBI | NP_001017890 | ZGC110623 | MMP23a | un |
| D. rerio | NCBI | XP_696707 | LOC568293 | MMP23b | un |
| D. rerio | NCBI | XP_699803 | LOC571143 | MMP24a | un |
| D. rerio | NCBI | XP_699765 | ZGC239J9.5 | MMP24b | un |
| D. rerio | NCBI | XP_684876 | LOC556862 | MMPLa | 3 |
| D. rerio | NCBI | XP_691470 | LOC563014 | MMPLb | 19 |
| D. rerio | NCBI | XP_685473 | LOC557327 | MMPLc | 10 |
| D. rerio | NCBI | XP_692946 | LOC564525 | MMPLd | 14 |
| D. rerio | NCBI | XP_694127 | LOC565762 | MMPLe | un |
| D. rerio | NCBI | XP_684533 | LOC553390 | MMPLf | 25 |
| D. rerio | NCBI | XP_685843 | LOC557654 | MMPLg | un |
| D. rerio | NCBI | NP_958911 | MMP13 | MMPLh | 21 |
| **TIMP** | | | | | |
| D. melanogaster | NCBI | NP_731461 | TIMP | TIMP | 3R 86A1 |
| C. intestinalis | JGI | ci0100139932 | ci139932 | TIMP | chr_05q:4329999-4335450 |
| H. sapiens | NCBI | NP_003245 | TIMP1 | TIMP1 | Xp11.3-p11.23 |
| H. sapiens | NCBI | NP_003246 | TIMP2 | TIMP2 | 17q25 |
| H. sapiens | NCBI | NP_000353 | TIMP3 | TIMP3 | 22q12.3 |
| H. sapiens | NCBI | NP_003247 | TIMP4 | TIMP4 | 3p25 |
| D. rerio | NCBI | NP_878294 | TIMP2 | TIMP2a | 12 |
| D. rerio | NCBI | NP_998461 | TIMP2L | TIMP2b | un |
| D. rerio | NCBI | XP_688461 | LOC559979 | TIMP2c | 12 |
| D. rerio | NCBI | XP_697272 | LOC568824 | TIMP2d | un |
