# Supplementary material for: The evolution of the vertebrate metzincins; insights from Ciona intestinalis and Danio rerio
Source: BMC Evol Biol. 2007 Apr 17;7:63. doi: 10.1186/1471-2148-7-63 (PMC1867822; doi:10.1186/1471-2148-7-63)
Supplement: Additional file 2 — Ciona sequences corrected from the original JGI gene models [file 1471-2148-7-63-S2.doc]

**Additional file 2**

***Ciona intestinalis* sequences corrected from the predicted JGI gene models**

>ci152455_ADAMa_ADAM

MRLGFLQLLLFLIETSIAEHPLHKKLHTWHTLKLRDMRHSRIKRSIMDDEIPTLDQLISFNAFNKTFRLSLLPSPEVFAMGFKAFVSGKNAEEEIRVDKAKVLKGTVLGDSKSHVIAHLQDGRLTASIDTDNETYIVEPMWRHMHAHDINEDTKSDMLVYRRSDVKLQSLNDPHGEDGFCDTHRLMREFRNKSKHDHNRNGIEDELETVRHQARHHRAKRSTAIPKICKITLVADHRFYREMGNSSIHETMYYLINLIERVNHIYKNTDWVTDPSQPKGVYKGYGFQIDNITIHNESTADPRYNQAHYWSEAKQLLEAFSEKKRSNCLSHLFTYVDFARGLLGLAYVGSGKKDDVGGICTKPYNRGNGDPTLYLNTGLTTTVNWGQRILTTEADLVTAHELGHNFGAEHDEEGAVNSADNCRPGQANGGNYIMCPAAVTGEYPNNKVFSVCSKRNILRSLKNKAPLCFQEEKNSFCGNFQVETHDNETCDVGYITGFSNEDRCCLFNCTLKPGAKCSNKNYKCCTEDCQIAGPTKMCRAHIPGLCLKDVYCNGVDKVCPRPKPMPDNTMCGDLGRCRNGVCEPFCRTKGLQPCLCSLLEDQFCSRCCAPLGVTGDELTNLCTPYLNISESHWTLPDNSRCTLGYCRKGKCHKQTQDVIERVWDIFKHISPDKIGQLLADNIVGGVLVFSLLFWVPCSCLINFVDKKRIKNRREEDKWRTQRGNEYTRNSMVFKGVSGSSAPVTPRIRIQRNNVAWNSGQVVKAGFGAKEQWKMSQAKGFNVRK*

>ci139599_ADAMb_ADAM

MKFNIAVLSIILFLCLQGELVTSMSKHVSYFEHLKYDVKNLAYQHERTRRSVSSSTKPVKLKFQAHGRNFDIHLHQDRTIFRPNLIIVDENNDKVDVNVSHVYHGVLYGQRESYVHGSIISGIFRGVIHEPNVGKFYVEETHQFFGGSQGQSPVAGYNGHSVIYHENDVIHPKKHSHHHCGASQPKVADWMKSYQNSSISEPVVTRHRRRRAVDPTKTTCLLYIQTDHILYEEYNSRDVIVSKIAEHVKAVNQIYTRTLFTTSTNERIRDINFMVQRIRINRTSAADDPFKSRNIGVEKFLDIASLANHNDYCLAYVFTNRDFDNGVLGLAWVGSPTGTSGGICEKYRTYTDRSAKSLNTGIVTFKNYGTVMPAVVTHITFAHELGHNFGSPHDGGVECTPGESPSIQTKKQGNFIMYARATSGKEPNNDVFSGCSIRNMSRVLEAKMSCFVSSDTPRCGNNLIDEGEDCDCGYSDTCADIGDVCCTPADDENNVERRCKLKAGKSCSITQGPCCNGTTCEAKNSNYRCSEETQCQLAQVCNLSKAVCPNATAKREAEICNHGTQICVQGTCSGSICAKHGLEKCECEIPPGSKNRTLLCHTCCKLPGNSSTCKSTGEYAQFNYSVIHVLPGSACENYNGYCDVFSKCRKVDADGPLSRLKKAIFNPALYNTIRNWIVEYWWAVMLMGLALVLLMAGFIKLCSVHTPSNNPRLPKHKQLPGAGTLRRRQNQRQVQQRRRRQQQQSSRDSYPMTHH

>ci131886_ADAMc1_ADAM

MELLKLVFICFVLVENSNASFFVGSSSSFASTLLTRLKQYTIVIPHQAEDKHARLKKRDLSTELHVVPGIHSKQTYFSFKFENQTYILELKLNEELLPKDFSTSIHTDDGQVIMDKPNLQERNHCYYQGHVVNMEGSHVIMSTCDGLKGCIALSDDFLLIEPLSPHSTQHVVYRPKDQTLNEAGTCGNQDMGHAVVNVDSFAGISALSVRRHREATTEKKFLELLIVADKAQYSRVDLDNRIKELVNYINGFYKKLKIHVVLSHIEVWKTQDKIPLKTNAQEVLNEFLSYRQRKISSEPPNSYWKYTDNAQLLYGGSFEGSTIGMASVKTMCTSRSGGVNQDHQTNVFYTATTLAHEMGHNFGMHHDSASCVCPPGAQCLMASGSSFKKDWSSCSKEYLAQSITEGLGNCLLNVPDPSRLYGGPKCGNDIIEMGEDCDCGGVDECLSQCCNATTCKLISGASCDTGPCCLGCQYSPAGQICRDTNNNICDLAEYCTGTSANCPGNVYRQNGSPCNGNQAACAQGVCLTHDLQCQGIWGEGAVSGNDICYERVNKLGNWNGNCGRGTSGFIICTKENSKCGKLQCSGGADRPIVARDRYAFKNTIDRKYECKTISSDDNATDVSDPGLVRDGTRCGDGKICNDGKCDDLPSMTCDATCNGHGVCNNLGNCHCNRGWAPPFCSSEGNGGSVNSGPITSNTMDTQTILMLVMFLVVFPIVVDPVYLSGIDIAREEHRSNHGKKEEKDRLEAVARAKKSGGKNLARTKDRVTSNSEEHLFDDGANEVEFRTSAVKKKPVLGWDSVNQWNDEPASTQKPVPIVMSMTKPTPPLEAPKIQNTPTRPPPPHPIAPSQVSVTVTPVRSAPPPPLPPPVQPQATTTLASNHPQPPRHPPPPAITKPSNQSAVRPNFPPPPPTTKPPLPPVGQPPPPVSKKPTPEKKPIFKPLVANKPHFHGSNPILASTTRPPPSKVLTQRPHSGGELPNDAIDPSQLSVQERMQRFGA

>ci140827_ADAMc2_ADAM

GGRVKRNVLTETKFIELLLIADNSEFQSIGSIAAIHSRFSTFTNIVDLLFRPLNFRVVLSHVEIWNQQNLINVVASNEMTLNSLRTYRDTQLAVGLANNVWKLTDTAMLITHSRGLTGIGIAFVSGMCRSTSVTLTHDFRNSTALVAMVMAHELGHNLGMEHDTDSCACSQPSCIMDTFANGGTDWTNCSRQQLDAFLQFHDPGCLLNVPNPSNIFTTPVCGNGLVEAGEQCDCGFPGSCNTLCCNAATCMLWPSAQCNKGLCCYNNCTFVHSGTICRNSSHADCDLPEYCSGISNQCPGDFFRKDSLPCNNGTGVCVSGLCLDRSTQCQEYWGPSSRNGASICYLVNTQGERYAHCGYTPPSTYAQCNLMDRECGLLHCMSNATTATLSGVSGLSFRFVVGGEACRSITITSPDTSDVGFVRNGMSCGTNKICMDRRCVSASNLGCDATCSNNGVCNNLLHCHCNIGWSPPFCNITGFGGSVDSGPASPIAETIIDSKLDFVYMTLLPTIKSEKLMPTVRVIENSLMNSLTCLTVGPQPYRIGILNCWLSKNSK

>ci143853_MMPa1_MMP

SNIFIQSYLEKFGYAAPPSYRRSAGNRMSSSYLEEALRAFQEFSDLPITGKLDEATLTKMDEKRCGNPDMTGTVNAKRKKRYALKGGKWDHKQLTYKFINYPTKLSVSQSESEIRTAFQWWEDNSSLRFSKVTKGEHADIEIMFAVGDHGDWDSFDGPGHTLAHAYFPVFGGNVHFDEAEPWSISSASGVKLGTVAAHEFGHSLGLSHSDVTTALMAPFYSPNTKGLHSDDIQAVQELYGKSLEKTDTVTQSPVPTTGKTEQTNTKVPPISSCNGRFDAITRLNNGSTYAFQGEMFWRLNDKNADKGFPASIRTVWGISGKVDAALTFNGNTDIFQGHILHRFRNGIRIQTSNISSIYRNIPSFVDAAFEWSGNGKIYFIKGQKYWRYNPSRGAVAFGYPKSLSVWGGLPKHIDSAMKWKGKTYFFENGQYYRFADYKMHVASSTESPYPRRADERWLGCRGNALMTGLAQNQKRTVKTADRRSPVLNFH

>ci149359_MMPa2_MMP

MRVLFFVSFISMLGSAIPAAVSQSQAVTYLMKFGHAPESNHMSGDLMTLSSLEDSIRSFQRFADLPITGELDESTLRKMGEKRCGNPDIAGTDNAKRKKRYALQGSKWDHKHLTYKFINHSPDLPASQVETEIRAAFQWWEDNSSLRFSKVAKSQRADIEILFSAGAHGDGDPFDGPGQTLAHAYFPGSGGNAHFDEAERWSISSGSGKVNLRIVAAHEFGHSLGLSHSDVSEALMAPFYNPRSSGLHSDDIQAIQRLYGKSMVKPDIVTPKPTVPTTTRRTTTPRTTRRTTTKVPPVGACNGRFDAITRLSNGSTYAFQGDMFWRLNDQNVDEGFPASIRTVWGISGKIDAALTYNGNTDIFQGHKVHRFRNGIRIQTSHISSRYGNVPSFIDAAFEWSGNGKIYFIKGEQYWRYNPSRRTVDSGYPKPLSVWRGLPKHIDSAMQWNGKTYFFENGQYYRFDDNRIQVASSTKYPYPRRADEWWLGCKDNTLMTGLSQNSENGGGSGGASKSGVQVSLVLLSSIVMIFTALV

>ci148688_MMPa3_MMP

MSLLIATAYAFPAPDPEEVLVRNVRSPDLGNLAKWNPATASKTDLTNIFTNLGNNARSQFPDVFDDVSNRFENAAERILERLRGNVEVQMTQLMTSFSDHDFDLVIRDLPRQIGHSSSRVVSKIVNLSEEYLGKFGYMSHKRAASAKLGGLVDKKSFAKSIREFQRMAGLPASGELNRETMRMMSMDRCGVEDRIGSVMMGSNGNEADSKKKQESKVKEKAKIRSKKQIKAINVEGDRWSNTKLKYYIENRTPDMTAAQVDDSIQRAFDVWANYTTLRFRQTRNPSEANFKISFGAGSHGDPYAFDGKGGTLAHAFFPSDGRAHFDEAEHYTYKGGPGVNLFIVAAHEFGHALGLGHSSVPNSLMAPFYQGYTANFQLHDDDIAGIQQLYGKKVDVDAKKKRPTKPPARPTAPTTTARPRPADAMNVCGKNISFDCMFFDQQKQSVFALKGKYFWKINDYGVVDGYPKKIYQYWKGLPGNIQAAAYSRITRRTYFFKGRRMWRFNGRVLDPGFPRDSSDLALPANPSAALQWGGDGNIYVFRKSYFYRFSEWQPFDGRKRYQHKKIAKFWTGIPNAPDAAIQWKNGKSYFFKRNDYWRFNGIDRDVDPNYPKSIKKLWIGCQGKTSAKLTKKNRKSKKGRKGRKNSRRTNTPPLARLELAPGSSNPQH

>ci138953_MMPb_MMP

MGDEEFLVALSNFQHANGLNITGVLDEETMMIMTQLRCGVPDLTVNPNITTSTGDPSDSDTESVTITPQINDIIADSGSAEGSGQRAENAEYMENETEWDTNSTEEAFMSDVESIVSEAELSTESRKEDMEEILSALRREKMARLNDSTINDTVPPPRFPELLERIVLYTRHQEHKRTKRSGAYRMFNRANGWGLAFPRRTITWRLWPSSRNQFMTSNEVWFTVKLAFRMWSEILPRNFLEDNVSPVSQVDVLIGFGINQHNRCPVSFGQGPLIQEYAHAWALPKAEIHFNDEQPFVPVSWIPATVSGGAPQTRSSASYPISLLKVAIHEIGHTLGLSHNDNRLSIMNPYYKPFQGSKNMDELSTSDRNSIKAIYGECEIPFDVAFDWLRQERSPDGVFRWKYNTYFFRRQWYWLYENSNKRPRYGDPKPTAYHWSGVMQLNDPHRKIDGIVHVREWNSPLGPIYFFKGDVFIEYDSRLARAMTSDSNGNRYPRPIRDLFPGVPYPIDTVFYKSDERILYFFKNHLVYRFHWTTKRLASIATIAATFPGWGGASPLPSNIDAAYYSYTEQAYFFYKGHYFWKMAGNAERLANPRMWIPLNAVGPRRRISSNWKDICDVGETELLDGFTSELDSSAAVFFYATKKEAATESEENTPGGDNSR

>ci146960_MMPc_MMP

QNFLVKYGYLAPHDRERSEDDLMAATSKFQKMYGLTDTGLLDEDTLYLMQQPRCGLWDIPSKRVDSELESKCRKKRYTLMGHWPSKRITYGFRMYTDDMSTLQQQDAVARALDVWSKNTPLVFTQVTIDPDIKPDKTILKDVDIMISFPRGYHNDGYPFDGPGHTLAHAFYPGXGIXDGDVHFDGDEKWTYMSQEDGTSLFMVAVHEFGHALGLGHSTSKGSVMLPFYQGYKEGISLSSDDIQAIYTLYGKGSEKQNGPLLSQDDPFVFASPTLGSTRGIFQENNKLPITQSPELCEKEFDAVANIRSDIYFFRGNYFWRILKDGRDDRIKMSRFWTNFPNVNVDAAYSRDEKTFFFSGTRYWAYSDTNMVAGSPRPISDFGLPMDGGIDAAMIWDSNGGTYFFRGERFWKYDEDRRQIELGYPRNISVLRDVPANIDAALSNPSDRSVYFLKGTRCWKLDDDLLSVSKGYPEYIGVTWFGCDR

>ci152154_MMPd_MMP

MAYNRSMFICTIQKIRCIYTFIFILFIIKCSESVSNFRLQVMQGSKLEEKKSLKLRDKRDADVQFAENYLVNYGYVPPDSLKSTGGAAELHSRSKALVEMQNFLGLTPTGTFDDATLEMMKKPRCANPDKMSVESNLRKKRYVTVGSPWQKNLITYSINNFTPKLGQKLTHEAIDDAFRVWGSFVPLQFKKVDASQNPDIVTFFAEGFHNDNTNFDGVGGYLAHAFYPGSGIGGDTHFDGAEPWTLHQGPNRGNDLFLVAVHELGHALGLQHSPDVKAIMAPVYKYHDTENFQLPQDDILGIQSLYGARYSGTERPIVPTTSKPTPAITTTTTRRPPSRPRKPTRRPTSRATVKPTTEPPEPVLCDLTSYDAISFLRKELYIFKGKYMWRQKRGRNTFNGPYKITSFWPQLKNGVDAVYENPTSNKIVFFKDHLFWEFRGTQLQPKSPRHVRYIGMQNGKVDAAVWWQRNGKTYFFKGDYYWRYGVGKVEGDYPKPIDVWRGVPPNVDASFSGVDGSFISFIKXGLNKLKTIHSNLKETKSXRQLKRALELNCSTNLKNTCKLSNVANKKCGRNVGMSIRSGALKAARNNNKREGIQRRNSAKEFTTVEEFKQCXIHNFTYFVKGTRFWTFNNWDIQVVKGALSFKKHWLHCGMIETEEKTFETAPPSAPVPAIIVVCMVAVALLAAAIAYVVWRRRRYSAKQATKTRIVETPGVPYQAGYGVPVPGREGEKTMCVTAAQQKMRWFRFSLQGRV

>ci151527_MMPe_MMP

MKLVFLVLCLSLFVGFIASAPAGRRQRNRNRYRNPRVLRVRGRWTQRDIANAESADSPQYRFDANGQGWYVNPNNRPAALNAPPLGAPGAPARFVQSFRSQLNVKFGNNGPPGVVAPSQVISPPVGPAPNPSIGARRPDSIVELTDDDKEQIALEFFVKANYTSNSLTDKTTLVRTMQRNYGLPITGRLDDTTVTYMMAPRCGHPDAPAEFNTFPNRDRWTSNTVTYKITGYTPDMSPCRVRNTFKRAFRVWEEATALNFVESQTDDADMIIYFGYQEHGDGYPFDGKDGLLAHAFAAGTHSLAGDAHFDEGEFWTLGDGRVADTYYGNADGAPCHFPFMFQNVEYTTCTTAGRSDGMEWCATTRNFDEDQQFGFCPHEALFTYGGNADGDPCTFPFNFLGETYNTCTYDGRSDGYRWCATTSSFDTDKKWGFCPDRARGTEGGNANGASCVFPFIFNGNSYSDCTTIGRSDYKKWCSTTDNYDTDGKYGFCQESGYSLFLVAAHEFGHTIGLDHSNSQGALMYPMYQKFTNFRLPQDDVNGARDLYPAARTTPLQPLVLEKCGGDLSVTEGGTDSGGTGTGTGTDDTNICSLSKVDGMLKFGDEIFIFLESQFWRWNIVTGSKSGPYPINSQWPDLPDSIDAVYRKPNDGPLVFFKGTRYWMFSGERLMAGYPKQVSTLGLPADANFKMDAALNWLRSKNRKRTYFFVGEQFYRYNEEKSKMDRGYPKPMSLWRRVPRNVDAAMEDPTRARYSMFFKENKIHYLNNYKVEVLQIDPLDIWLGCRTRVASAVPLRAGPSRDRGSP
