# Supplementary material for: The evolution of the vertebrate metzincins; insights from Ciona intestinalis and Danio rerio
Source: BMC Evol Biol. 2007 Apr 17;7:63. doi: 10.1186/1471-2148-7-63 (PMC1867822; doi:10.1186/1471-2148-7-63)
Supplement: Additional file 3 — Zebrafish sequences used in the analysis [file 1471-2148-7-63-S3.doc]

**Additional file 3**

***Danio rerio* sequences used in this analysis**

>Dradam8_ADAM8a_ADAM

MRYTGLFITLLSFVYTWESLEALPHVMRYDVVRLQALRGRTRRSASSLQKYPEQLEYDVAIDGRNLTISLHRNRELLGKQYTLTHYGEDGISETKSSNKFNHCYYHGHIHNFEDSSVSVGLCSGMEGFLRVNDQVYLIEPLEESLDGDHAIYKQEHLRTKRGAYGYINDTVYDLGPKSSGLYKGKNMRNKAPRGGQQIVEMVLVVDNTEYKKFGSFKKIEERMMLVANHVDKLYRPLNIRVMLVGLEVWSQRDLIDVSSRPNLTLERFLKWRRDSLLQRKKHDNAHFITGIDFEGSTVGLATLYAMCSSSSSGAVNEDHNSNPIAVASTVAHEMGHNLGMSHDDSSCGCSSNKGCIMGDTIGSIYPDSFSTCSQSSLKAFLENYDTNCLIDVPNEGQIYGGPVCGNAFVEKGEECDCGTVEECNNPCCNATTCRLTEGARCAHGECCHNCQLKHTGSLCRKSAHDCDLDEYCTGESAFCPEDDYKMNGLPCNYNQGYCYNGQCPTHKEHCKMLWGSGADVDDDACFQYNVIDRTSKSAEHRKCGRIYCYGGNPFPKTNKKANVNVQSRTCYMALDSSPTEDLGMVPTGTKCGTNKVCYKSLCLDISMYGTENCSNKCNNRGVCNHELKCHCDPGWAPPYCDIQLSELHKMRKSVVIGVTTSLAILVLIIIIGALVYNRNKITEFRKKRPQKGIHSSSGQCNPAFQPGSAKNSPRIAQPRISQPTFLESSATQACKPLRSAAMPCRPAPMPPKNAPQTRNEQIMKPPVPPSAISKNIYPPQAKPLLPAAKPLPPSRPLPPLASKAVTKSKSPPVPPVKPSGPQPVFTPPQVIQKVALKPPAWPR

>DrLOC556905_ADAM8b_ADAM

MSKRLRNSEVCADCSTSGCVAESIRTLPHVERFDVVRPKRLNLSKVQSDQSPQSHEKYPDRLAYKLFLGGENHVIHLEKNKQLVGHNYTEIYYQDDGSIVSRNPSFKDNCYYHGHIQDMEYSSVSVGICSGIRGFVRVKQQVYLIEPLANHSDGDHALYKHQHLRRRRSSAGEPKTMFYDHEPQVSAALKSSRWNMKSFQTPRFVELFLVVDNTEYRNFGSSMDSIRARMLEVVNHVDKLYRSLNIRVMLVGLEVWMKQDQIVVSVSSDDTLSRFIEWRKSNLLKRVKHDNAQFVTGIDFLGDTVGLANKFAMCAESSAGVNQDHNQNPLGLASTIAHEMGHNMGMSHDEDHCTCGSSVISSFCIMTESVGTLFPEQFSDCSLEQLTVFLDNANPSCLLDTPSSYKLYSGPVCGNAFLDPGEECDCGSVEECKNPCCDPMTCKLTEGSRCAQGDCCENCQERVTYLCKPIAFSSVCVTVRVWCAWYPLQGVPKRWKGFGCEESDSCVDLCCSHRGLITNTKRFFDHLPLLNFSAALASDKNP

>Drzgc101824_ADAM9_ADAM

MAHAVCSRLVKNAFMWIMLHTLVDCCGGSVTQQTSQLSSYEVTVPRRISRQRRNQDSADKVSYIIQAQGKEHVVILERNELLLPEDFTVYSYAKDGSLVTEKLNTTDHCHYSGYIEDVEGSSAALSLCSGLRGVIHTEGTSFGIEPLEGSPSDEHLVYRLEDVQTQPLNCGTPHSYNHDDQSSPRQPIISHTHNITFGQAGSHLRRKRAVLHQTHYVELLMVVDNERYNYKNRNHTAVREEVVQIANYVDSMYIPLNIRIVLVGLEICVLC

>DrLOC566044_ADAM10a_ADAM

MSPYSVVKIGSFKEERRTGGEKRQADETKSSSAKHFNLRMKRDTSLFTHNLKVEVSGVEVPYDTSHIYTGEIYGEKDSLTHGSVVDGKFEGFIKSHHGTYYVEPSERYLKDQNVPYHSVIYHEDDIDYPHKYGSEGGCADHSVFERMKKYQASAVEEPTQTNHIIDEEVADGPVILRTRRAAQAEKNTCQLFIQTDHLFFKYYGSREAVIAQISSHVKAINSIYQGTDFQGIRNISFMVKRIKINTTIDEKDSSNPFRFGNIGVEKFLELNSEQNHDEYCLAYVFTDRDFDDGVLGLAWVGAPSGSSGGICEKNKQYSDGKKKSLNTGIITVQNYASHVPPKVSHITFAHEVGHNFGSPHDSGSDCTPGESKTTDQKEKGNYIMYARATSGDKFNNNKFSICSVRNISQVLDKKRSACFVESGQPICGNGLVETNEECDCGYNDQCKDQCCYSADEPEGKKCKLKPETESNPSQGPCCTPQCTYRTGNECRPESDCAFKGLCNGLSAQCPASTPKENYTACHANTQVCINGGCSGSICEKHKLDVCTCATEDGKDDMELCHVCCMEKGKPSTCSSTSSERWASFFDNKVITLQPGSPCNDFKGYCDVFMKCRLVDADGPLARLKKAIFNPELYTSIAEWIVKHWWAVLLMGIALIMLMAGFIKICSVHTPSSNPKLPPPKPLPGTLKRRRAQNPSAQTQPQRPRQQRENYQMGQMRR

>Drzgc64203_ADAM10b_ADAM

MRLSVMSVFPVLPVLLGLLCASGPARGYYGNPLNKYIRHYEGLSYDTEMLHSDHQRAKRAATHTERTLQLEFHAHGRHFNLRMRRDTGLFADDFKLEVSGQEMDYDTSHIYSGEIYGEQGTLTHGSVQDGRFEGFILTHRGTFYVEPVERYLQDKNVPFHSVIYHEDDIDYPHKYGPAGGCADASVFQKMKRYQSTAHQEPTDSPPAQDEDVLLRKKRMTQVEKNTCQLFIQTDHLFYKYYKTREAVIAQISSHVKAIDAIYQGTDFMGIRNISFMVKRIRINTTSDERDRSNPFRFANIGVEKFLELNSEQNHDDYCLAYVFTDRDFDDGVLGLAWVGAPAGSSGGICEKNKLYSDGKKKSLNTGIITVQNYASHVPPKVSHITFAHEVGHNFGSPHDSGSECTPGESKSPDKKERGNYIMYARATSGDKLNNNKFSVCSIKNISQVLEKKRGSCFVESGQPICGNGLVEPGEQCDCGYSDQCKDDCCYDANQPDNKKCKLKPGKVCSPSQGPCCTFECSYKGHNEKCREESECAHQGLCSGSSAQCPTSEPKANFTACHGNTQVCLNGGCSGSICEKYGLEVCTCASVEGKDETELCHVCCMEKMNPSTCSSTGSERLSRFFGKRVITLQAGSPCNDFKGYCDVFMKCRLVDADGPLARLKKAIFNPELYENIAEWMMAHWWAVLLMGIALIMLMAGFIKICSVHTPSSNPKLPPPKPLPGTLKRRRAQHSSQHPPPSHQQAVPHAHALPNPRPAPRPQPARHHRSARENYQMGQMRR

>DrLOC567145_ADAM11_ADAM

MGKYKILTVRRGPRTVQSETKYVELLVVNDYDLFVQMRRSSPQTRNFAKAVVNMADAIYKEQLNTRIVLVAMETWSTQNMVSVGDDPLVTLRDFMKYRKENIKEKSDTAHLLSGRTFQSSRSGTAYIEGICSPTRGGGVNEYGNVGPMAITLCQSLGQNLGMMWNKDRATAGDCRCPDPWLGCIMEDTGYYLPRKFSRCSIEEYIQFLQQGGGSCLFNKPNKLLDPPECGNGFVEQGEECDCGSQVDCSRAGGACCKKCTLTHDAMCSNGLCCNRCKYEQRGVICRDAVNDCDVPETCSGDSSKCPHNVHKLDGYMCDAGLGRCYGGRCKTRDAQCQALWGHNAADRMCYEKLNIEGTERGNCGQDSSSHNWIQCNKQDVLCGFLLCTNITVKPRYGDLHGESTSLTIYHQNKYLDCRGGHAVLEDGTDLGYVEDGTPCGPNMMCLDHRCLPVTTFNLSSCPGSSFSLVCSDHGTCSNEVKCICDSDYTGKDCSVYDPIPDPQPPDGPEKYKGPSGTNIIIGSIAGAILLAAIVLGGTGWGFK

>DrLOC563275_ADAM12a_ADAM

MGLFLRPACSQQEGPIDQPPARGLSHRQNHRPFGIYAEDLLEAVRGLGFSETTLIHIFLAGLDEPFDWEELGDIRTWTFWEVVDYSCLRMEREILEGEWTPLTTTTSTISWSMSSPQGLTRTQKRRRKRKASSARHKRDVQKTTKYVELIIVADNREFQKQGKDVEKVKQRLAEIANYVDKFYRVLNIRVALVGLEVWSDVDKCSVTQDPFTTLHEFLDWRKLKLLPQRPHDNAQLISGVYFQGTTIGMAPIMSMCTAEQSGGIVMDHSDNALGAAVTLAHELGHNFGMNHDTPERGCGCRMTVDRGGCIMTPSTGLGAMRTRSLKYEDRPRNPLAGVRPSIFWLPPQNPKQAFTDPQRVQMFLASVFCSPHLPPPFPLSSAVPVDRGQAGGTEVVKEPEFNIELFRGSQLKVVLKRETGFEMYPFPTVFSTCSKKDLVASLEKGVGMCLYNMPEVKMLYGGQKCGNGYIEEGEECDCGEPEECLNPCCNATTCTLKGDAVCAHGQCCENCQLKPAGTPCRESSNSCDLPEFCTGTNPHCPANVYLHDGHACHNMDGYCYNGICQTHEQQCITLWGPGAKPAPGICFERVNSAGDPYGNCGKDAKGSFAKCEARDARCGKIQCQGGANRPVIGTNAVSIETNIPLQEGGRLLCRGTHVYLGDDMPDPGLVLAGTKCAEGKMCLNRQCQNVSVFGVHKCSAKCNGRGECNNKKNCHCEAHWAPPFCDRAGFGGSVDSGPMRQAADSSAVAVGSLVTLLCLLSAGLMLCLKRKALVQFLCANKKNAIQKLRYRNPSCRNVFDPGFLLISIWGHKEKEREENIYQLDESKHPALLRHSQSSGSHLSTSPLHYPYTPAQTSVFLPMKRGADEMSHPSSEPLLPAHSFQSLRRMPECPSRCQPVHISHPMPITLQPHRTLRATVVHTLPRHIAYTHTQDHDMPSPPQKPLPAHPLIRSHGMGRNVRIPAPLAIPIPTVPRPPPSIPLPNRPLPRLPQPFYDH

>DrLOC558872_ADAM12b_ADAM

MIEKLAVPVDRGQAGGTEVVKEPEFNIELFRGSQLKVVLKRETGFEMYPFPTVFSTCSKKDLVASLEKGVGMCLYNMPEVKMLYGGQKCGNGYIEEGEECDCGEPEECLNPCCNATTCTLKGDAVCAHGQCCENCQLKPAGTPCRESSNSCDLPEFCTGTNPHCPANVYLHDGHACHNMDGYCYNGICQTHEQQCITLWGPGAKPAPGICFERVNSAGDPYGNCGKDAKGSFAKCEARDARCGKIQCQGGANRPVIGTNAVSIETNIPLQEGGRLLCRGTHVYLGDDMPDPGLVLAGTKCAEGKMCLNRQCQNVSVFGVHKCSAKCNGRGVYFLTGTALQDKLIVGNKDV

>DrLOC561244_ADAM12c_ADAM

MRVTYPQKILLQQLCLLIQSLQSYRITQTRQSVSEKTLYGKTGSMGSVKSFTHPFPQTYYQICLLEHECLNPCCNATTCTLKGDAVCAHGQCCENCQLKPAGTPCRESSNSCDLHEFCTGTNPHCPANVYLHDGHACHNMDGYCYNGICQTHEQQCITLWGPGAKPAPGICFERVNSAGDPYGNCGKDAKGSFAKCEARDARCGKIQCQGGANRPVIGTNAVSIETNIPLQEGGRLLCRGTHVYLGDDMPDPGLVLAGTKCAEGKMCLNRQCQNVSVFGVHKCSAKCNGRGECNNKKNCHCEAHWAPPFCDRAGFGGSVDSGPMRQAGTVIESCTFAMP

>DrLOC555937_ADAM15_ADAM

MKQHHHQLFRCFWRTLVTILLFEGTPLMMFRGSLTLTLHRNDSSFTPQPDARITHSSHNVTNHRREQKPLAMTRPFAFVDGVRRSLSDVLQKGHPDRLQCGLQVGSSLYILDLEKNQDLLPKPPNVFYYLPNGTGVSMKENTVSKRDILSEIKYIELVLVADHKEFYRPLNVRVALLGLEIWSDQDKIKVDKSPTETLNRFLDWRTRELLPRLRHDNAQLIMGESFDGTTVGMASQSSMCSKDRSGGVNVDHLVSVLGVASTVAHELGHNLGMSHDTADRRCHCQNEPRLGGCIMEPSTGFMPGQLFSSCSERDLSLSLLHGGGMCLFNVPQPESLLGGPRCGNLYVEKGEECDCGLLDECNDPCCNASTCKLVPGAQCSSDGICCENCKLRVAGSVCREPLGECDLPEHCTGSSPYCPPNVFLQNGEPCKGGSSYCYSGVCASLDDQCQMLWGQNSTRAPPICFSSVNKQGNKYGNCGQMPNGSYIPCLKCDCSFFIHACVDQKCRDVSMFGVDECRSKCHGHGVCNSNKNCHCDEGWAPPDCRYSGNGGSVDSGPAQEPRDSDPARVALLVIFLFVLPVSLLFVALRFPRCRRGLVYLGHTPFNKTRQSRSRTGLLLQLNLFRPIHLSLNLLSLTQRLLFSVTCLHPQHYVQPTGARPAAPSKPLPPDPVLSSRQNPVPPKPPVPKKPLPVDPSCHPPVPPLMGHPVSAAASSSSYSSRNSASAAAPARPAPHPPLRRQQYHGKPHSSHPV

>Drzgc63886_ADAM17a_ADAM

MKPCIFYLVLLAPLFAHCAVKPVSVEEVSRNEEELQEFDALNSLLSDFEVLPASGLQLHSVRKRDADAHSHVERLVSFTALQRNFKLYLTTNTQLFTENFSAVFVDENGKEDQFDVQIQNYFKGHVVGEEHSRVQAHIDGDEFSAHIITEETEYNVEPLWRFTETPPDGRLLVYRSEDIKNISRLSSPKVCGYVNADAQELLPEEARAAEEMEEMKEEPHNRERRSTHDQKKNTCTLLLVADHRFYKHMGRAEESTTLNYLIELIDRVDDIYRNTSWDDDFKGYGVQIQQIIINKDPTKVAPGEFHYNMDGSPTGRKDGVWDVKKLLEQFSYDIAANASRACLAHLFTYQDFDDGTLGLAYVAPSKPGLGGLCPKPYYPSQSVKKPSYLNTGLTSTMNYGKTILTKEADLVTTHELGHNFGAEHDPDNIASCAPSDDQGGKYVMYPIAVSGDHVNNKHFSTCSKISVSKTLRIKVNQCFVERSSKLCGNSRVEEDEDCDPGLLHINDDPCCTSNCKFRKQAQCSDRNSPCCKNCMFESADKVCQEVITATCKGTSQCTGNSSECPTPGNLEDNTECVDKGRCQNGQCRPFCEAVHNLESCACNETENSCKVCCRKDELCTPFLNGGSFLYLRKGKPCTVGFCDGAGKCMKQVQDVIERLWDFIDKLNINTFGKFLADNIVGSVVVFSLLFWIPISILVHCVDKRLDEQYEENTKMMYPSSGEILSSLESVPVRIFKAHHVHSPAARPQLSMAPTGPGSTPAQETLGGPGHPSLSAAPKADQLRMATIEEDPSGDSFHPDEEEGFRLSGTAARSFEDLTEQATPSHKRRLKRQARIESKETEC

>Drsidkey81b152_ADAM17b_ADAM

MKRLLFFLASLFLTEGSRRPIRDVRDDEYDHLSSLLSDFDVLQLSSLQQHSVRKRDVQSQTHAERLLGFTALQRHFKLYLTTNTELFTHDFKAVVVGEDGAQEVYEVQRENFFTGHVIGEENSRVQAHIGDNDFTAHILTDEAEYNIEPLWRFTENTHDSRLLVYRSEDIRNVSRLAATKVCGYVSADASEVLPESVRVSRALDEDHQEEEGQVRERRQTVDHSKNTCPLLLVADYRFFKHMGRKEESTTLNYLIELIDRVDDIYRNTSWDDEYKGYGVQIQQIIINMTPTQVEPGGAHFNMKGTPVKDKYVWDVKKLLEQFSIDIADNASQVCLAHLFTYQDFDEGTLGLAYVAPSKPGFPGECPPSGNDNRAIYLNTGLTSTKNYGKTILTKEADLVTTHELGHNFGAVHDPDDMSYCAPREDQGGKYVMYPIAVSGDHTNNKLFSNCSKMSIAKRLRAKASTCFKERNSNVCGNSRVEEGEECDPGLLHLNSDLCCTANCKLKPGVQCSDRNSPCCKDCRFEKQNKVCQEPMEATCKGRSNCSGYGSECPPPENLPNKTICVDNGRCLNGECIPFCEAVKNLQSCACNETHNSCKVCCKDKNGVCAPYIGDKGSPLFLRKGKPCTVGFCDGAGKCMKQVQDVIERLWDFIEKLDINTFGKFLADNIVGSVVVFSLLFWIPLSILVHCVDKKLDQQYELSTKPLFYPSNAELLSSLESASVRIFKPPSSAGSSVVPRFHACGPLQTSTPPVSISQVAPAPIQTPTPCPLPNIEPQRMATIKEDPSYDSHLDQVLGEDFPTSGSVSRSFDDLTENRENAMSFRVNRHPQSNSKETEC

>DrLOC565342_ADAM19a

MLLKINRFLSLVECLLSDCGGSSYKKLEFDPEWLAILKATDNLQKPSASFWNPFVYEAESGTAVLQTDIYILYRKQNKDLDKTKLRIMEIANYVDKFYRELNIRVPLIGLEVWTEHDQCIINEEPNSTLWSFLQWRQKLKSRKKHDNAQLLTGVIFKGTTIGMAPLEGMCSHENSGGINVDHSELPIGAAATMAHEIGHNFGMSHDHEGCCVEATAEQGGCVMAAATGRRMLKQAGTMCRGPAGACDLPEYCTGGSPYCPSNVYLLDGSSCQYGRAYCYNGMCLTHEQQCLQLWGYGAQPAHDACFQDVNAAGNAFGNCGKDSKGNYMKCEKSDAKCGKIQCHSAAKKPKGTNAVSIDTTIQTDGIEVKCRGTFVYSTQDGQGDLPDPGLVMTGTKCGEGKVCKDRRCQNTSFTELESCIVRCHGHGVCNSNGNCHCSRGWAPPFCEKPGLGGSVDSGPVQYDSQVGLVVGLLFAFLVLLPAVLIAFYCVKIKSSYYHKWRKQREKSKASSSKVLFCMQVCNSNGNCHCSRGWAPPFCEKPGLGGSVDSGPVQYDSEFTFNFHCTYASPLALL

>DrLOC571252_ADAM19b_ADAM

ARPAPDLCFTDVNKAGDAFGNCGQDLMGTYRKCTERNAKCGKIQCQSSASKPLESNAVAIDTTIRSGQQKILCRGTHVWPHGHAKENQNDTLDPGLVLTGTKCGDNAICFEGECRDASFLQADECSTKCQGHG

>DrLOC566725_ADAM22_ADAM

MRRDCSAAISRNPRSVEDETKYVELMVINDHLMYKKHRLSVGQTNNYAKSVVNMADLYFKEQLNTRIVLVAMETWAADNRFNINDDPMVTLREFMKYRRDFIKEKCDSVHLFSGNRFHSNWGGASYMGGVCSLTKGGGVNEYGKEMAITLAQSLGQNIGIFSDKKRILNGECKCEDKWSGCIMDDVGFYLPERFSDCNVEEFHDFLNSGGGACLFNKPSKLLDPPECGNGFVEAGEECDCGSPAECAKEGENCCKKCMLTQGAKCSDGLCCKNCQLEFMGVLCREAVNDCDIPEMCTGNSSQCPPNLHKMDGYTCEKDQGRCFNGRCKTKDRQCKYLWGEKATSADKFCYEKLNIEGTEKGNCGRDKDTWIQCKKQRPRGLVLIQDEER

>DrLOC563160_ADAM23a

MPLGEGPKPSAKNAERRPTDISGRTRREEPVAGPSSYQRQGYCSCCQVLYNSVEQHILSAQHREVVRASRANVSSGSLLERFLQDVLQHHPHHYSDTRPSHADLPLLTTPLVPKEVLSEVYCGSDDDGVSVGTREEMPTSDEESCQMLQVATAGPAVTTDFTANTHLQTASQKGKMVPEQPPPEKYSPTQGFLHRTSGSSANDKLHTSQFTLPVQRESSHTHPSRHPPSEGAPVSKTGAVEHRKAHKKTNRENEGSDTIKASCAPPAAKCITSKDQPETFRSVTEESTKWISALPPWKGLHREQTFSNLSDQIRDVIEEVIEKYCYGLNVDQQEEDGSFLFGPQSVSDSKGSEEWDNAIQVALGKTKGEEKNLAQLLEVHIDLEDQTYQTQLDTALNTVGTPEEPKQNAEDNVIPDLPHIPQSFVGKTWTQVMFEDDIKIDSIVREFQQDILPLIEHNEEDPKHSAVLVRKTRQKYYRQASRCQVVKVSHGTQTIPLSCPMVRSKPTQETFPSTYNDEPQQCPSPERTPEMKTRLCALKLPTSYCKIMSPLQPKTVVYVLSSPDGGQGISKPTPIKKAGRKRKSCDSDGGLKYKYKKTPLKFYDPLTNRILKSPPKGMSSPPNSKSLSHVRQLFRSLSPDINKERQGQSPGSSRKGRGRRNMVDLCTTTSDSILESGGPSEPSSSLTSSRRAIFTRSSISSRSCLLLGHLTPSAHVDDSSKAHSHSCAGSSYKVGGMEQEEHIQQTSVDQTPIRRSLRRAGLLTPAKRPSAPPYRTKRKSAKQQRKVKSQGSLQRSVSSHISAECRTSPRNKSPVSSSVQNEALRDQHVSETPREATAPDDSTEGKTGEHPVTITYPSRLIYYLNEESESTFHDLDTRAKNQAGDGQDVHLAQASFQLDAFGTTFTLDLTLNNDLLSSDYIEIHYENDKPVLSRGGEHCYYHGHVRGKEDSRAALSTCNGLHGMFDDGNFVYLIEPQKQTHTAETEARPHTLRRTSSLGLTSIPDDWTADDPDEEKRIFASMPWLKRRRKRAMPRNIFEEMKYLEIMIVADHNTIFKEQLNTRVVLVAVEIWTDKDRIPLNFSKYRQQNININADAVHLFTNMTFHYGRSSVGYIGGVCSKARGIGVNERSVAAWILGWDVLWKTLGVFLPALLHRVQHPRKFSKCSITDYRNFLLRGGASCLFNKPNKLFEATECGNGYVEVGEECDCGARMECYKDCCKKCSLSNGAHCSDGPCCNGTCLFYPRGYSCRFAVNDCDISETCSGDSGQGRCYSGECKTRESQCKYIWGPKAGGSEKHCYEKLNTEGTEKGNCGRDGDKWVPCSKHDVFCGYLLCSSTGRIPRIGTLKGDVTPTTFNHQGRLVDCSGGHVLLDDDTDLGYVEDGTPCGPSMMCLERKCLPISSLNLTACPSGPGGRVCSSHGVCNNEATCTCDATWAGTDCSMHDPRKHPPVIEDPDNKVSVATNRLIGAVAGTILALGVIFGGTGWGIE

>DrLOC565131_ADAM23b

IFKEHLNTRVVLVAVEIWTDKDRIPVSVTPFEMLRNFSKYRQQHIRQHADTVQLFSNVTFHYPRSSAAYFGGMCSASHGVGVIEYGTQWTMALQLSQSLAQNLGIQWDSASKRKDCGCVNSWPGCIMEDTGVQHPRAFSKCSIVSFKEFLLKGGGSCLFNKPSKLFEDTECGNGYVEVGEECDCGPRDECYKDCCKKCSLSNGAHCSDGPCCNNTCLFYSRGHTCRYAVNDCDISETCTGDSGLCPPNLHKQDGYFCSLNQGRCYAGECKTRDSQCKYVWGP

>DrLOC558008_ADAM28_ADAM

MAQRHLMLWIFTLCVSLDPSVGHIHELHGKVYEIVRPIRLHDLQKRDLQSRPDRVKYAMTLGGRDIEMHLQKNIGMLTKDYSETYYTDDGMLVTTTPADLDLCYYHGKILNDSASLVSMSTCDGLRGYFQTAEQRFLIEPLSEDSDGDHAVFKYEDVNEATPRVCGVTNTTWDESGDGVPPRILKTRSRSSGPTLFQRQKYNEFFLVADNREYKKLNSDLEKLRKRIFEIINFINNVYKEINTFVALTGFEVWTDNDKITVSAASGATLDSFTKWRNSDLIKRQRHDNAHLLTAIDLDGATVGLAYIGTLCGGLSTGIVQDHNSMATAVGATIAHEMGHNLGMNHDSSSCVCSDSSCIMTAALSYFIPQHFSSCSTGAFVDYLNNKIPECLLNKPQPRDLLQPAVCGNGFVEIGEECDCGTVQECKNPCCNATTCKLTVGSQCAAGECCDNCKIMSASHVCRPKADDCDLPESCTGKSAECPEDVFTVNGVPCKNGKGYCYNGQCPMKEEQCIKMWGSTSVVARDYCYDQNTRAEYYAYCKRNGDKYIGCQKQDIMCGKLFCESGNDNPNYGRLVTFSNCKATFYGSPDEDYGQVDTGTKCGEGLVCNQNECVGLETAYKATNCSNKCKGHGVCDHRLQCKCEPGWLPPDCDKPAESEGLSKGVTIAISVIVTIVIVILLIGLTVFLCKRRTSTPHAYRSQPRQQKVHVVDIPDLSQQRSATPNQKPTPVIKPSAPPPPPPYSVHAPHNDFMAARQALRPPPPRV

>DrLOC570620_ADAMLa_ADAM

MVGTANWRGKQRLRARHVGYLCLLRPESTSLLALLLLPCTMGSTRIGWAVLHQESSAVTEQSSAMLKIICLWAQKTAHLKIAVLLERNRTGTSSRDGEWVLQPGGEHCYYHGHVRGVPRSWAALSTCHGLQGMFSDGNFSYGIEPLHNSSDQDANIHVVYRMADIRLMPHFSVCCILIFSMDQFSVIDIFIDPLKADEWELFVRDGFHPYMASSLPGSTRNSSDSDINNDYPVSMETSQLELTDGLRRAKRQSTLVFCVKHYSHSEIFTQNTVGFGVALQVRRGPRTVQSETKYVELLVVNDYDLFVQMRRSSPQTRNFAKAVVNMADAIYKEQLNTRIVLVAMETWSTQNMVSVGDDPLVTLRDFMKYRKENIKEKSDTAHLLSGRTFQSSRSGTAYIEGICSPTRGGGVNE

>DrLOC568376_ADAMLb_ADAM

MESSKQASQAISKYIKHYEGLSYDRELVKQHQHRIRRDANPNKQDLHLDFSAFQREFHLRLTPDVNNGFTEDFKVQSENESQMVDLSHIYSGVLEDDSKSSCQGSVLEGQFEGSITTSNGTFYVEPIERYKTTHSDHHSIIYHEDDVGVSRSLAGVQAGPRDHGWDSSVTVVFQEVSCSLLVHDRHSVSSGYGLVQDYGYLGIISSQVLDRINAHNFHLFGGRIEKADVNHLLIFSLDAFFDLQQIPVSRSKRKVDQSKTSCLLHLHTDHRYYKRFGSIEAIVAQVASYMKAVNDVYDKANFDGIELINFKVKFLTVITEEDPSSPISVTHVGPEKLLSLFSETNWNDFCLSYLLTDRDFSGVLGLAWEGKADNWGGICSKMILKSGRNCSHNTGLVTLQTYGHYLSTKHVHLTFAHELGHSLGAPVSLMPLFTVIASWIKSPTFGLQI

>DrLOC560975_ADAMLc_ADAM

HDENSNCGDLEVTSGKGRFLMFPKAASRIEENSDKFSPCSLRHMSHLLNVKKDTCFVDSDQPICGNRIVEEGEECDVGHDDSDPCCHSSKEPSGIECRLKLGKQCRHLQRSPPRSFLSLLLEMLCVFKEAGLMCEGNSECRNKSVCAGSSAVCPEPPSKPDMTICSNGTRVCSSGECGLSLCALHNMVQCDCPGQSKTEKCHMCCQQRDKPNTCASTTSAVLLQYFSGKRVALVPGAPCSGNQGYCDQFKVCRILDADGPIARLKNSFLKLDEFDDLAGWMK

>DrLOC570656_ADAMLd_ADAM

MTRHVNIFVLILINFSICPVQAIDQTSYLKKYDVVKPQLVQMRWRRYADPSHKQPEERHADIITYSVRIEGSDRILHVTKNTDFLSKNFVVISHKTPKKGKVQPERMVQCYYQGHVEGYEDSLVALSTCEGIRGVIIIGNKSYGLEPVLHSKANEHLLFLLEDSHSEPFVCGLENETSLSEDHSRYADMSMFLRKKRTLPQTKYVELALVVDQKR

>Dr_LOC565145_ADAMTS1

MSFGALDLPIHYMIIFPQTDWRHYAFKMFDSAGEARCSFSGTVNGDELSSAAINLCKGLHGGFSVGGEEYFIQHENSTGAATDRDTHTIRRRTPGILAEESGSKCGVNEEEERVTEKPHTTHSKPSESKAHHRSRRFVSTPRYLEIMIVADQSMAEFHGAQLKPYLLTIMAVASRLYRHPTIHNSITLAVVKLLVVHDEEHGPHVSTNAALTLRSFCQWQKQHNQPSDRHPEHYDTAVLFTRKDLCGAHSCDTLGMADVGTACDPDRSCSIIEDDGLQAAFTVAHELGHVFNMPHDDAKQCASVNGDQWGAHMMASTLSNLNQLDPWSPCSALMVTSFLDNGHGQCLLDKPQKPEELPQTLPGSVYDADRQCRLTFGEESQHCPDLSSTCAALWCTVTSANGLLVCQTKNFPWADGTPCGSNSYCMAGQCLSKTEAAKYEVRTIWFAICSVQFSFRDCVNPRPKNGGKYCEGKRIQYRSCNTETCPDSNGLTFREEQCLAHNDLSSQVSLGSGEGVEWVPKYAGVSPKDRCKLVCRAKGTGYFFILKPKVADGTPCTPDSTSVCVQGQCVKAGCDRVIGSDKRFDKCGICGGDGSTCKKVSGSMERARPGYQDVVTIPAGATHLDVKQRSFGGRSQDNSYLAVRRQDGSYLLNGDYKLTTLETDISVKGALLRYSGSSVLLERLRSFAPLPEPLTIQVLSVGESPRPRVKYSYFAPRPNGSNRPSINAISKAVDAEWFLAWFLGEWGTCSKTCGGGIQKRDVMCLDHNHRPSEDCVELRPASSRQCALQDCPFWLPGNWSECSRTCGRGFRKRTLSCITHDGHILPNSSCNATDRPRPLLNLCNLTPC

>Dr_LOC571682_ADAMTS2/3_ADAMTS

MIRLFDRSAQGQATAEQLARLRQAGRSVAEYAIQFKTLAASCDWNQWACRFMFHTGLEDEIQEELATIDLPHDFDDLINMALRVEGRLRRRSSHRLVFHPSWKTEDTLPTSDASTSASTGPEPMQFGDLFTSCLALIDSGAEGNSMDRSLAALWGIPALSFSDPIPACSLNGTLITTVSHSTPPVTLIVSGNHPWLAQHSPHVELADLAGVPAENHDLRQVFSKSRATSLPLHLPYDCAIDLPGTSPPKGSPLGGLAALELLPQHVKKKINSRARRETYEDEIFNIEVLLGVDCSVVMFHGREHIQKYLLTLMNIVNEIYQDSTLGAHINVVLVRIMMLCSSKSMGLIELGNASQSLENVCRWAFLQQKEDKNDAEYHDHAIFLTRQEFGPTGMQGYAPVTGMCHPVRSCTLNHEDGFSSAFVVAHETGHVLGMEHDGQANECGDEVPMGSIMAPLVQAAFHRFHWSRCSQQELRRYLNTYDCLRDDPFDHEWPTLPQLPGLQYSMDEQCRFDFGMGYMMCTAYSTYDPCKQLWCSHPENPFFCKTKKGPPIDGTKCASGKNCFKGHCIKLTPDILRLDGHWGQWTKFGSCSRTCGGGVRFRTRECNNPVPANGGRTCYGNNYEFQLCNTEECAEALVDFREEQCKMWDPHFEHEGNKHHWLPYEHSEPDERCQLYCQSKETGDVVSMKRMVHDGTRCSYKDPYSVCVRGECEVRC

>Dr_LOC565053_ADAMTS5_ADAMTS

MWLVVLLCCVLDASVARPPANASLLAPDGTVRAVDRIYHGGGKAGYLLYLDEQRFQLDMERDETILDHQFSADAPPRRECVYRGTVNSNAQSLAVFNLCGGGLEGFFALDHSRYTITPVIRAKGHENDVHIVEDADATRALHLYTRERFSFEAMPERHSCGTRDRKTRKHKKEKHGRRWWTKFIKPDASPTRRKRSVSRARHVELLLVADASMTKKYGKDLQHYLLTLASIASKLYGHASIENPIRLSAGSDQMSDYCGNVSLSPSCSTVNLHIPDAAGDLGFSLNWTAAQRPHGCNPTLTLHHLTCGFGVFRVSRRSLFVLFMFCLPQADIPLSVSPYQDLCGHHSCDTLGMADVGTVCSPERSCAIIEDDGLHAAFTVAHEIGTQTDHRQTCTHLTECLLDAPRVPLLGPEELPGQSYDAVQQCRLAFGSEYSVCPGMDICARLWCSVIRKGQMVCLTKKLPAVEGTPCGKGRICLQGKCVDKTKKRHYSSFRQEQCEARNGPQTDPKGVKTLVEWVPKFAGVLPKEAGKLARRAKGTGYYVVFSQRVVDGTECRPYSSSVCVKGKCVRTGCDGIIGSKLQYDKCGICGGDGNSCIKVAGNFTKKSKGYTDVVKIPEGSTHLKVRQYKAKGQSRYTAYLALRRPGGDYLLNGKLMISTSETIIPLNGSVLNYTGWSQRDEAFHSMGPAALQESLLVQILSTDAKKPLDIRYSFFMPRKTPLPARTSPEAPPRMSLEAPPRMSLEAPPRMSLEAPPRMAPEALPRTSPEAPPRIPPEAPPRTPPEAPPTVASLSSAELVTFTSDVPPTTESPKFPRWLTGPWMSCSRTCDTGWQSRTVQCKDANGKLSKNCPLSARPSAFKHCLIKKC

>Dr_LOC569024_ADAMTS8a_ADAMTS

MWSYLVIFVCLFAETSARLFETEEIVPVQLTGRSGGRVAKRSETQPSFRLSAFGRNFTLNLTPDSTFISPALKVYRVKVKPQEKSTSNLTDLYQSLNQTEETGTEFLKGCFYTGFVDSSEDSVVSVSLCRGILGSFISDGKEYLIEPKSFGLGTKGKLTEQLHVIKRRRFAKSSQVSKQLSDMRADLQNSRIQMPSRRRRFVSTPRFIETLVVGDASLTHFYGDEIKHYMLTLMSVAAQVYKHPSIKNSINIVLVKMLIVEDEEVGPSISSNGGVALRSFCAWQQLFNPSSHRHPEHYDTAILFTREDICGHQSCETLGVADVGTMCDTKRSCSVIEDNGLQAAYTTAHELGHVLSMPHDDTKSCEQLFGHLGEDHIMAPVFTQLSKTSPWSPCSALYVTEFFDNGHGDCLLDAPETTVALPTELPGLTYSLDRQCQQIFGEEFSHCPNTSSSEVCERLWCQQEGQSMCTTRNGSLPWADGTSCGTNRTCLNSVCMSSEDVLRPQPAVDGGWGEWGSWQPCSRSCGGGVMFSYRECNRPSPQNGGKYCVGQRVNYQSCNKQACENNRGKSFREEQCEKYNNPNHFDIHGNVKQWIPKYAGVSLRDRCKLFCRLISCLPERKDIS

>Dr_LOC568205_ADAMTS8b_ADAMTS

MWSYLVIFVCLFAETSAHSFETEEIVPVQLTGRSGGRVAKRSETQPSFRLSAFGRNFTLNLTPDSTFISPALKVYRVKVKPQEKSTSNLTDFYQSLNQTEETGTEFLKGCFYTGFVDSSEDSVVSVSLCRGILGSFISDGKEYLIEPKSFGLGTKGKLTEQLHVIKRRRFAKSSQVSKQLSDMRDDLQNSRIQMPSRRRRFVSTPRFIETLVVGDASLTHFYGDEIKHYMLTLMSVAAQVYKHPSIKNSINIVLVKMLIVEDEEVGPSISSNGGVALRSFCAWQQLFNPSSHRHPEHYDTAILFTREDICGHQSCETLGVADVGTMCDTKRSCSVIEDNGLQAAYTTAHELGHVLSMPHDDTKSCEQLFGHLGEDHIMAPVFTQLSKTSPWSPCSALYVTEFFDNGHGDCLLDAPETTVALPTELPGLTYSLDRQCQQIFGEEFSHCPNTSSSEVCERLWCQQEGQSMCTTRNGSLPWADGTSCGTNRTCLNSVCMSSEDVLRPQPAVDGGWGEWGSWQPCSRSCGGGVMFSYRECNRPSPQNGGKYCVGQRVNYQSCNKQACENNRGKSFREEQCEKYNNPNHFDIHGNVKQWIPKYAGVSLRDRCKLFCRARGSSEFRVFAPKVIDGTPCGPDTTSFCVQGQCIKAGCDLEIDSSKKLDKCAVCGGNGQSCRMISGSFNKVVHGYRDIVTIPSGATNINIKQQSHGSIPHDGHYLAVRRENGNYILNGNFSVSTVEQHIPVLGAVLKYSGSSTTLERLQSFRQLQEPITIQLLSTAAESIPPKVKYTFYIPKSMAFSKPKDKKIAGKLIHPFGVPQWISSEWSECSKTCGSGWSRRNVECKDNAGFYSNHCNKDLRPSDIRPCADLPCPIWQIGPWSSCSQTCGHGERQRKILCIDYTGKTVEPENCDPAKMPESVSEKCFYQEC

>Dr_LOC565478_ADAMTS8c_ADAMTS

MSQFALCELNEEEETVLVNRTFRKGLFWRSEERQHFKLSAFGHLFLLDLSPDSKFVSPVLNVQRIKAKNLRSVLDASRSGGALRDATLYPDSGADLRDCFYTGTVNSEKESVVAVSLCHGIHGTFITQGVEYFIHPKASVQTTGKHFPQVHVIKRRAVSNSRTASLVFDQMKVDSLVKESKLNSSEDEDGNMRRAKRFVSAARYIETLVVADASMTRFYGDEIKHYLLTVMAMAAQVFVHPSLKNAVSLVVVKMVVVEDEEVGPELSSNGGVALRNFCMWQQLFNPGSQRHPEHYDTAILFTREDICGYKDCDTLGVADVGTMCDPKRSCSVIEDNGLQAAFTVSHELGHVLSMPHDDSKNCEKWFGHLYGHTMAPFFVHLNKTLPWSPCSALYITEFFDNGHGRILLCLYFVSQVPVNGGWGEWGPWGPCSRTCGGGVEFSHRECTSPVPQNGGLYCVGQRVKYQSCNTQTCPEDHGKSFREEQCEKYNSDRYMDIKGNMKQWIPKYSGVSPRDRCKLVCRAKGSNEFKVFEAKVVDGTTCGPDTTSICVQGQCIKAGCDQVIGSNEKLDKCSVCGGDGTTCRKISGSLNKATIGYIDIVTIPAGATNIDIKQRSHRGIPHDGNYLAVKAQDGTYILNGNFSVSMAEQDVPVPGAMLRYSGSSTTLERLLSFHRLQEPITIQLLSTAGDISPPRIKYTFFLPRDVPFSKPGTESRISTHVISPFGGFDWVLGEWSECSKSCGAGWSRRSVECRDGEGSLSYLCDADLRPADIRPCGDLPCPMWQMGPWSACSRTCGPGERHRTVVCLDYAGKVLEPQKCNPDKRPEVAVGECFYQDC

>Dr_LOC569618_ADAMTS8d_ADAMTS

MSQFALCELNEEEETVLVNRTFRKGLFWRSEERQHFKLSAFGHLFLLDLSPDSKFVSPVLNVQRIKAKNLRSVLDASRSGGALRDATLYPDSGADLRDCFYTGTVNSEKESVVAVSLCHGIHGTFITQGVEYFIHPKASVQTTGKHFPQVHVIKRRAVSNSRTASLVFDQMKVDSLVKESKLNSSEDEDSNMRRAKRFVSAARYIETLVVADASMTRFYGDEIKHYLLTVMAMAAQVFVHPSLKNAVSLVVVKMVVVEDEEVGPELSSNGGVALRNFCMWQQLFNPGSQRHPEHYDTAILFTREDICGYKDCDTLGVADVGTMCDPKRSCSVIEDNGLQAAFTVSHELGHVLSMPHDDSKNCEKWFGHLYGHTMAPFFVHLNKTLPWSPCSALYITEFFDNGHGDCLLDPPEMTIPLAAELPGHTFGLDQQCQQAFGNKYTHCSNAPADQTCVQLWCREEGKIQCTTRNGSLHWADGTPCGEDRRCREGLCLSSAMEEAGEQKVPVNGGWGEWGPWGPCSRTCGGGVEFSHRECTSPVPQNGGLYCVGQRVKYQSCNTQTCPEDHGKSFREEQCEKYNSDRYMDIKGNIKQWIPKYSGVSPRDRCKLVCRAKGSNEFKVFEAKVVDGTTCGPDTTSICVQGQCIKAGCDQVIGSNEKLDKCSVCGGDGTTCRKISGSLNKATIGYIDIVTIPAGATNIDIKQRSHRGIPHDGNYLAVKAQDGTYILNGNFSVSMAEQDVPVPGAMLRYSGSSTTLERLLSFHRLQEPITIQLLSTAGDISPPRIKYTFFLPRDVPFSKPGTESRISTHVISPFGGFDWVLGEWSECSKSCGAGWSRRSVECRDGEGSLSYLCDADLRPADIRPCGDLPCPMWQMGPWSACSRTCGPGERHRTVVCLDYAGKVLEPQKCNPDKRPEVAVGDCFYQDC

>Dr_LOC566130_ADAMTS9_ADAMTS

MLSKLQEFGAYEIVTPARLNEVGEQLPTGVHFKRRKRSTDPTTANISHHWTSPHAYYQISAFGQDYYLNLTLESGFIAPVYTVTILGASSEGHNSVEGEEEEDTEYQHCFYKGHVNAGQEHTAVISLCSGLLGTFRSPEGEFFVEPLHSYNSEHYEEEHIKPHVVYRKDASKKTVDDSAACETSGHTEPNRRHRNRLKRKSPSSMLSDLETLNSRLFPFSENKHNSANESSDSKPHRRSKRFLSYPRFVEVMVVADSKMVEHHGSNLQHYILTLMSIVSSIYKDPSIGNLINIVIVKLVIIKNELDGPTISFNAQATLKNFCIWQQSQNHPDDNHPSHHDTAILITRQDICRARDKCDTLGLAELGTVCDPYRSCSINEDNGLSTAFTIAHELGHVFNMPHDDSNKCKEDGVKNQQHVMAPTLNYYTNPWMWSKCSRKYITEFLDTGYGECLLDEPVSRPYSLSQQLPGQIYSVNKQCELIFGPGTQVCPYMTQCRRLWCTSPDGVQRGCRTQHMPWADGTDCAPGKHCKHGLCIHKEHEYVPVEGAWGVWSPFGTCSRTCGGGIKIAVRECNRPVPRNGGKYCVGRRMKFRSCNSEPCSKQKKDFREEQCASFDGRHFNINGLPPNVRWVPKYSGILMKDRCKLFCRVAGSTAYYQLRDRVTDGTQCGPDTNDICVQGLCRQAGCDHVLNSKARRDKCGVCGGDNSSCKPVAGTFNIVHYGRSHRQISSSHISRLQCSGENSQWCYKYRCETTQLLRETLSNSRGEYLLNGDFVVSMFKREVRVGNAVIEYSGSDHVVERINCTDRIEEEIIIQVLSVGNLYNPDVRYSYNIPIEDKPQHFFWDAYGPWQDCSLLCQGERKKKILCNRESDRVVVSDQRCHGLPKPAAITESCNTDCELGWHIARKSECTAACGVGYRSLDIYCTKQSRLDGKTQKVDERYCSSQHKPNDKEVCHGDCNPGGWEYSSWSECSRSCGGGTRRRNAICGKSDERDDDSKCNPQEKLTAQPCNEFLCPQWKTGDWSECLVTCGKGYKHRQTWCQFGEERLDVRFCDSSKPESVQACQQQECASWQVGPWGQCTTTCGPGYQMRAVKCVVGSYGSVMDDTECNAATRPTDTQCSATCGKGTRMRYVSCRDQQGGVAEESACAHLPKPPASEVCSIVACGQWKVLEWTACSVSCGQGTTTRQVVCMNISDQVVELSECDLDDKPAAEQECAMPQCPSRSSDHGGFSPNPDFRKKTALPGRTDRNRAGRLQAQQWRTGPWGACSSTCAGGFQRRVVVCQDENGYPAISCDESIQPIEQRSCESGSCPQWFYGSWSECSKSCGGGIKTRLVACQRPNGERFNDLSCEILDKPPDREQCNTQSCSINPHWSTDQWSLVRS

>Dr_LOC558722_ADAMTS12_ADAMTS

MINTGWIFCNRKLFPLGPCRGGSKGPRAGRGPAVQVLELASTVVGRLYSTQQGDYTDHKYGNKLKMASTKEKLIAHVSKEQPAGPTNKVTVVGVGMVGMAAAVSILLKRGLLNLPEGPLLIEPVRGRSPNLTHPQHPHVLYQSSTWSRFRQRRSIDAHQQSPCGVKANAAGRCDAVPFRSCRPTAYLLVEGFPAATRLSTQLIVYMVLGYFQQVEQDRELWERESVDRNHRPDPERPRRISPRSISRERWVETMVVGDSKLVDYHGSGSVESYIFTIMNMEKHAAGVPREHPTADIGPQLIKLTSAQSEALLKASFIQVAGIFHEASIGNAIHIVLVRLILLHGEEKGLKIIHHADTTLTSFCTWQKNLNPQSDTHPAHHDVAILITRKDVCAGMNQPCETLGLSHLSGMCQPHRSCNINEDSGLHVAFTIAHELGHSFGIQHDGQGNDCEFVGKQPFIMSRQLQYDSSPLTWSSCSKEYITRFLDRGWGFCLDDRPSKRDLSTPGAAPGVRYTPQHQCQLQYGPNATFCSEVENVCQILWCSVNGSCRSKLDSPIDGTRCGPEKWCISGECVIVGKLPETVHGGWGPWSSWSHCSRTCGAGVQTADRECNQPKPEFGGKYCTGERKRYRVCNTKLCVRKHPSFREMQCSEFNTVPYHNELYQWIPVSSPSRPCELHCKPVDENFSEKMLDAVTDGTPCFMNNNSRNICVNGVCKEVGCDFGINSNAKEDSCGVCLGDGSTCETVKENFVEQDGFG

>Dr_LOC570801_ADAMTS13_ADAMTS

MHRPLTAPPAGGGPAGGWSHIAPSGISPTGFRGKKPGHQAHAYEPQPQSDLPPKTYLPCETLPGALASNNIALKDHSGTQTPPLRSEFTKLELFNAANCETCRMNLRFQSDEFACQGLIQSGEMRLYIRPVLEQHIDNLKDLTMDLPPGVPHLLITQTLPVRTRLKQPKSQFRLRRSPLRSEVTYLELAVVVGPDVYEVHRQDTERYILTNLNIASELLRDVTLGANIRVHLVRMIILTEPEPDIQISENITSSLKSVCEWGQKVNPDADSDPLHADLLLYITRFDLVLPNGNKLVRGVTQFGGVCSTQWNCVITEDTGFDLGITIAHEIGHSFGINHDGIDNTCSSSGFMMASDGGYNSVDLTWSQCSRAQLYSFFRAGKAECVQDVPVLGGSVQDWRPGLFYGVDDQCRIAFGSSATACSFTNEDMTTCRVLSCHINPQDHSSCTRLLVPLLDGTECGPSQWCLKGRCVSPSTSGSSMMVHGSWSAWSDFSPCSRTCGGGITHRRRQCNNPRSAFGGTICKGQNTEAELCNLQLCDSTQLEFMARQCSATDQQPLSVSTDSKSMYTWIPAISYSSGDSQCKLMCRSREQDFMVSRGSQFIDGTRWEF

>Dr_LOC565549_ADAMTS15a_ADAMTS

MDHVKNSLVDDEDSILPRKAVFKISAFEQDFVLELRSDSSFIAPGFSARTDSAAHNSAHGEDLSRCFYAGEVNSDPYSYAALSLCKGLQGAFGYDGWEYFIKPASNNTDRDNEAAHLIRRRSNGDLSGNSTSRCGVDSNATQTVAQSLAKFKHLKEQHMNNVTMSFLRNRSKRFVSIPRYVETLVVADESMAKFHGDDLKHYVLTLMSVAAKLYKHPTILNSINIVVVKFMVINEEEKGPKVSGNAAMTLRNFCTWQKKLNKNNDKHPEYWDTAILFTKSQLCVPIRMDHVKNSLVDDEDSILPRKAVFKISAFEQDFVLELRSDSSFIAPGFSARTDSAAHNSAHGEDLSRCFYAGEVNSDPYSYAALSLCKGLQGAFGYDGWEYFIKPASNNTDRDNEAAHLIRRRSNGDLSGNSTSRCGVDSNATQTVAQSLAKFKHLKEQHMNNVTMSFLRNRSKRFVSIPRYVETLVVADESMAKFHGDDLKHYVLTLMSVAAKLYKHPTILNSINIVVVKFMVINEEEKGPKVSGNAAMTLRNFCTWQKKLNKNNDKHPEYWDTAILFTKQDLCGSSTCDTLGMADVGTMCDPKRSCSVIEDDGLPSAFTTAHELGHVFNMPHDNVKACEEVFGKLQDNHMMSPTLIRINRTSPWSPCSAAIITDYLDSGHGDCLLDQPEKPLALPDVLPGASYGLERQCELAFGAGSKPCPFMQAPCQRLWCTGKTRGQLVCQTRHFPWADGTSCGDGQLCMRGTCIDKQELLKTKVDGKWGKWGPFGSCSRTCGGGVQLSKRECDNPVPVNGGKYCQGVRVKYRSCNLSPCPDTGKSYREEQCEAYNGFSLNTNRLTSSVVWVPKYSGVSAKDMCKLICRANGTGYFYVLATKVVDGTPCSPDSSSVCVQGKCIKAGCDGKLGSNKKSDKCGVCGGDNKNCKKVSGLFTKPMHGYNFVVMLPMGSSNIDIRQRGYKGITSDDNYLAVKNNQGKYLLNGNYVVSAMEKDILVKGSLLRYSGTVGSSETLQAVKPLGEALIIEVLSVGQMTPPRIRYSFYLPRESKDNKVQKKEEKARAENSILREEGGVNKDIASKPGKWVAAGWDVCSVTCGNGLQRRMVQCLGGDGGPGVDCEPSQKPSAIQACGDPCPMWDVGDWSPCSKTCGKGFKRRLLRCITTVGKLLPREKCVDKKKPQELDFCSLAPYPPLARARLLHKRMRSERAAGRSQMPSRSRRRLGRFGMSPHILRSFYICTVESILSGCITTWYGNSTSSNHNGLHRIVQTAGRVVGGELPSLQDIYTRRCMRKAKRIISNFSHPSHRLFSLLPSGNRFRSIRSRTS

>Dr_LOC568151_ADAMTS15b_ADAMTS

MTCSTVGKGKEAAMFYMQIIWQFMQNWPRTGFHGPVMSTFDKPSKELVVYRINAFNQEFYLNLLPDSSFLAPDGTFQDTSSPSALSGDELRRCFYSGDINADMNSYAALSLCGGVRGAFSYNGMEYIIERRTAPELISDDAGKTHVIRRKNLNAPNSSKCGVTSNQEVMESLDKYKHLKGHTKNLTETLLRSMSRSKRFASIPRYVEVLVVADESMAKFHGDDLKHYLLTLLSVTAKLYKHPSILNAISIVVVKLIVINEAEKGPKVSSNAALTLRNFCTWQKKLNKVNDKHPEYWDTAILFTKQDLCGATTCDTLGMADVGTMCDPKRSCSVIEDDGLPSAFTTAHELGHVFSMPHDNVKACEDVFGKLKDNHMMSPTLIQIDHHTPWSVCSAAIITDFLDSGHGDCLLDQPQKLMAIPEDPPGISYSLGRQCELAFGSGSKPCPYMQACSKLWCTGKAKGQLVCQTRHFPWADGTACGTNKLCYRGTCTEKQNTFKNKVDGRWGRWGLYGPCSRSCGGGVQLAKRDCNNPVPENGGKYCQGLRVKHRSCNLEPCKDSGKTFREEQCEMFNGFTLNTNRLSPSVVWVPKYSGVSVKDRCKLICRAIGTGYFYVLAPKVVDGTPCSPDTSAVCVQGKCIKAGCDGKLNSNMKFDKCGVCGGENKNCKKVSGMFTKPIHGYNFVIDLPIGAANVDVRQRGYRGLVNDDNYLAVKNHHGKYLLNGNFVVSAVEKDIIVKGSLLRYSGTGTSVETLQTSRPLKESLTVELLSVGKMTPPRVRYSYYQTVGNKESKIFKKEERIPAQNSVLEDSNKVELKKPVYQTPSYKWVTADWSKCSVSCGNGVQSRLIQCLGSDGKMATNCDGSQKPSSMKVCGDPCPTWSIGEWSSCSKTCGKGFKRRPLRCMTQTGQFLPRDHCSGKKKPQELDFCTVRSC

>Dr_LOC569571_ADAMTS15c_ADAMTS

MDHVKNSLVDDEDSILPRKAVFKISAFEQDFVLELRSDSSFIAPGFSARTDSAAHNSAHGEDLSRCFYAGEVNSDPYSYAALSLCKGLQGAFGYDGWEYFIKPASNNTDRDNEAAHLIRRRSNADLSGNSTSRCGVDSNATQTVAQSLAKFKHLKEQHMNNVTMSFLRNRSKRFVSIPRYVETLVVADESMAKFHGDDLKHYVLTLMSVAAKLYKHPTILNSINIVVVKFMVINEEEKGPKVSGNAAMTLRNFCTWQKKLNKNNDKHPEYWDTAILFTKQDLCGSSTCDTLGMADVGTMCDPKRSCSVIEDDGLPSAFTTAHELGHVFNMPHDNVKACEEVFGKLQDNHMMSPTLIRINRTSPWSPCSAAIITDYLDSGHGDCLLDQPEKPLALPDVLPGASYGLERQCELAFGAGSKPCPFMQAPCQRLWCTGKTRGQLVCQTRHFPWADGTSCGDGQLCMRGTCIDKQELLKTKVDGKWGKWGPFGSCSRTCGGGVQLSKRECDNPVPVNGGKYCQGVRVKYRSCNLSPCPDTGKSYREEQCEAYNGFSLNTNRLTSSVVWVPKYSGVSAKDMCKLICRANGTGYFYVLAPKVVDGTPCSPDSSSVCVQGKCIKAGCDGKLGSNKKSDKCGVCGGDNKNCKKVSGLFTKPMHGYNFVVMLPMGSSNIDIRQRGYKGITSDDNYLAVKNNQGKYLLNGNYVVSAMEKDILVKGSLLRYSGTVGSSETLQAVKPLGEALIIEVLSVGQMTPPRIRYSFYLPRESKDNKAQKKEEKARAENSILREEGGVNKDIASKPGKWVAAGWDVCSVTCGNGLQRRMVQCLGGDGGPGVDCEPSQKPSAIQACGDPCPMWDVGDWSPCSKTCGKGFKRRLLRCITTVGKLLPREKCVDKKKPQELDFCSLAP

>Dr_LOC559948_ADAMTS18_ADAMTS

MRTDAAKLGVACSAQLTINSSRRPTDFMPEWGSFNPLNHEMFQEWCHHTKDSSRNELPLGGDDYDFATPVEVDSYGGYISHDVSRHGRSRRSLSEAGSLVHYHVSAFGKELHLDLQPSHVLAEGFTVQTLGAEGINTATLDPSIHNCFYQGSIRNHSDSSVALSTCTGLSGLIRYSSEELFITPLPQHLALQHNYSAPSGHHPHVIYKRSAERRVHADKTDRTSRFKSSNPYEDHHHHHHHHRHHDYQHGKLQRQHFCGRRKQYTPKPPTEDHFVMPDEFEQQIRVKRSEITSSKEGGLNVETLVVADRKMLEKHGRDNVTTYVLTVMNMVSSLFKDGTIGNDINIVVVSLLLLEKDPLGLSINHHADQSLNSFCQWQSGLVGKNGKRHDHAILLTGLDICSWKNEPCDTLGFAPISGMCSKYRSCTINEDTGLGLAFTIAHESGHNFGMIHDGEGNPCRKTEGNIMSPTLAGNNGVFSWSACSRQYLNRFLGTAQASCLVDEPKQIGQYKYPEQLPGQLYDADIQCKWQFGSKAKLCGLDFVKVNITKAFTEIEFIQSLGYLNDICKSLWCHRTGHRCETKFMPAAEGTICGADMWCRKGQCVKFGDHGPKAVHGQWSGWSEWSDCSRTCGGGVMYRERSCNSPSPQHNGKFCQGPGRLHQLCNTKPCQPDGVDFRAQQCAEYNSKPFRGWYYKWKPYTKVEEEDICKLYCIAEDFDFFFAMSSKVKDGTSCSDYKGGVCIDGICEPVGCDQVLGSKAALDACGVCKGDNSTCKFYSGQYTLQHRANEYYPVVMVPAGARSIQVQEMEISNSYLAVRSLKRGTYYLTADWTVDWPGRFQFAGTTFNYQRSFNRPETLFATGPTNETLLFESKEIPHVAKPKNKGPLLVAGLFHSIRVILRLSSVALGTDLILLQGKNPGITWEYTLPHPEKKHNYTWSVVRSDCSAPCAGGRVSTKAICLQDQQTQVNSSLCNPQTRPVVGSHLCNTQPCPAYWTTGKWGVCSRTCGGGQQTRNIRCLRKVTYQREEVAAQSLCPCSKTCGRGLKKRSVFCRSTDPGARAVVVPDSMCKLHLKPKAQESCVLSRCPKNERLQWITSAWGECSASCGAGVRRRELHCGEKDSQGGYTEFPARRCRNLQKPEADLEQSCNNGPCPEPLAPQLLQPGGPSVTLGWYSSPWLQCTVSCGGGVQTRSVQCLRQGRPSVGCFPHQRPISSRACNTHFCPAPIPVPVPPPSPTLKEELAEHDKTSHALTSSAGVIWCLSMVFAITNSTVNNAMATMDKVDICEMWYRKRNSNSPCLGLIRDNESLVSEELKNTLD

>Dr_LOC560595_ADAMTSL2a_ADAMTS

MDKVDSLHSYLRRRRHVTGETLSEAKECPSTGRSFREEQCWSFNSQVYNGKNYHWKPLYPDDYVHISSNPCDLHCTTSDGQRQLMVPARDGTSCKYSNYRGVCVDGKCEPIGCDGVLFSPNTLDKCGVCQGDGSSCSRVTGNFRRGASSLGYTFITQIPEGSWDIQVIERKKSADILAVTDQAGNFFFNGAYKMDTPQNFHAAGTIFKYRRPTDVYETGIEYIVAKGPIDQPINVLVWNQNGQNPYITYEYTVMRDSMASVSQPPIYTGPQGGSSLVSVEVVSVLQHNQSMYDKNQEQTTGPRAVEGQKPAQETNEVYETANIDCEQDANTQVQYTEGNCSWPSAVGAPGTATVSRPAEDMVNSENLIWRVLLGERLGSDGVLANISTNQLLTHRDGLSSEAEPMELDYSSLEQAGLNGSLLEFTLGNRRNDTDLLLPNRNLTAILRNRGNRTRNNHRLLQKNKPSAADMYRWKLSTQEPCSVSKSLAMCVRYDGIEVDDVYCDALTRPEPVHDFCIGRECQPRWEASSWSECSRTCGEGFQFRLVRCWKMLAPGLDSSVYSDLCTEAQLERPPERRACKSPTCGPQWEVAEWSECPAKCGRKSLVTREVRCSDEAHACDEETRPPSTKNCTGPPCERQWTASEWGPCSGICGHGKTVRHVYCKTAEGRVVPESQCSPENKPLAIHPCGDNECAPHWLAQDWERCNTTCGRGVKRRTVLCVSITSGKVQINEDEECDASKRPADEDTCFERPCFKWYTTPWSECTKTCGVGVRMRDVKCYQGRELVRGCDPLTKPVNKQTCALQPCPTEPPDENCQDRPTTNCSLALKVNLCSHWYYSKACCHSCRNLRSS

>Dr_LOC561364_ADAMTSL2b_ADAMTS

MVHPGTRMSFYREEFQRRAVLVIQLSDDYVHISSNPCDLHCTTSDGQRQLMVPARDGTSCKYSNYRGVCVDGKCEPIGCDGVLFSPNTLDKCGVCQGDGSSCSRVTGNFRRGASSLGYTFITQIPEGSWDIQVIERKKSADILAVTDQAGNFFFNGAYKMDTPQNFHAAGTIFKYRRPTDVYETGIEYIVAKGPIDQPINVLVWNQNGQNPYITYEYTVMRDSMASVSQPPIYTGPQGGSSLVSVEVGSVLQHNQSMYDKNQEQTTGPRAVEGQKPAQETNEVYETANIDCEQDANTQVQYTEGNCSWPSAVGAPGTATVSRPAEDMVNSENLIWRVLLGERLGSDGVLANISTNQLLTHRDGLSSEAEPMELDYSSLEQAGLNGSLLEFTLGNRRNDTDLLLPNRNLTAILRNRGNRTRNNHRLLQKNKPSAADMYRWKLSTQEPCSVSKSLAMCVRYDGIEVDDVYCDALTRPEPVHDFCIGRECQPRWEASSWSECSRTCGEGFQFRLVRCWKMLAPGLDSSVYSDLCTEAQLERPPERRACKSPTCGPQWEVAEWSECPAKCGRRSLVTREVRCSDEAHACDEETRPPSTKNCTGPPCERQWTASEWGPCSGICGHGKTVRHVYCKTAEGRVVPESQCSPENKPLAIHPCGDNECAPHWLTQDWERCNTTCGRGVKRRTVLCVSITSGKVQINEDEECDASKRPADEDTCFERPCFKWYTTPWSECTKTCGVGVRMRDVKCYQGRELVRGCDPLTKPVNKQTCALQPCPTEPPDENCQDRPTTNCSLALKVNLCSHWYYSKACCHSCRNLRSS

>Dr_LOC567853_ADAMTSL2c_ADAMTS

MTAFIHKLHREDSSEGREDLQRHPDEVAQWWGEWSSWSTCSRTCGGGVRSQERHCLQQRLTSTQNINSSFCIGPSKQYQLCTNQPCPNSRISFKQHQCSQFNAKAFSRKHYEWVPLYPDDYINISNKPCDLQCTTTTGERQLLVPAHDGTFCRDGIYQGVCIEGQCQVVGCDGKLYSSKTIDKCGVCGGNGASCYRVSGTYRKGITQLGYVFITNIPVGATDIQIIERRKTENILALSDEAGHFFFNGNSVIDNPQNFHVAGTVFKYRRPANLFSDGFEYIMAQGPTQQALNVMYYNLNGKMPHITYEYTVPRAPAFPMTSTPEQPQISNLMSKTSRDSSVAEPKGNTSSTEHNEIGARKQHEKKSNQSSSLVPQDNVDVHEELLWGLQASVNFSELPALVLFRPASEVLQNELDDEIHHLEAQENFGPDSNLIDGDSSSSSDSNTSIPLLKSLLYDGASWSSRQPCKDSSALSGCSILKLNSSAPLDNSLTLEIYPGSLDLHSPDGEPRTNDSTNHQSNILQLHQMEPVQAPHSESNEFEVESLDHDVSMSDMYRWKVSAYAPCSSTCTTGISTSYALCVKYDGTEVDESYCDALTRPEPTHEFCTGKECLPRWETSRWSECSRTCGEGFQFRTVRCWKMMAPGFDSSVYDELCQAVELQKPMTRKACKSKSCGPQWEISDWSECSARCGGRGVRSRDVRCSMEARLCNESTKPVSQRECEGPPCDRRWTVSDWGPCSGPCGEGRMKRYVVCKNSNGKVISDGQCDPELKPLAVHPCGDKNCPAHWVEQEWEQCNSTCGRGVKTRQVVCAGLEAGVFKEFPERMCDRALRPADTAACFERPCSKWFTTSWSQCSKTCGIGVRVREVKCYQGEELGHSCDTTLRPEARQSCEVQPCTTEPPADDACQDKPTANCALVLRVKLCTHWYYRKACCLSCRNKSQ

>Dr_LOC557465_ADAMTSL4_ADAMTS

MVVLRACPTKRRTRGRPKACWRDYVSQLAWECLGMSLEELEEVSWEREVCTVCAIIFHRQNPGIDYEYYIPVDKRRDGETEVLRERERGRSALRGDPLESSCRHASLSSIESEGCDGVLGSGLVRDRCGVCGGEDGTCERVSGSFMNTSVPLGYHKILDIPPGATAINITERRASPNYLGMNSVSGVSMAVENPVAPPPVSSSFPSSSSSSSSSSDRWPPERPRPQGLGPNRNARIPPRTDLPLDNLPVFVWRRGALTECTATCGKGSQYREIVCVNRHTEQEVHERRCDSATKPTLEEEPCNVHPCPPFWDMGAWSECSVSCGWGVQQRQIQCRQSFGNRSTMVHPQRCAGLSRPNATQPCHPRVCSHWEISTNWSTCSRSCGEGLQTREVRCLTPDKQHSATCHPDARPAHQQPCNTIPCSPFEDENCKDRRHNCVMVVQARLCVYSYYKTACCASCTQSAQRAKRH

>Dr_LOC558887_ADAMTSL5

MEPGGGGGGAGRRDDQVRVRGRAVPEIRKEEEEGDWPAARSIVQSFSWTDGRSTATSGDANEKPCALYCTPVGSDSPVLVAERVLDGTPCGPYESDLCVNGKCQKIGCDGIIGSSAKEDRCGVCNGDGKSCRIVKGDFNHSKGMGYIEAAVIPVGARRIKVVEDKPSHSFLVWVVCFFGASPHRNSPGCGKDSEGGLIREQYMFGIVPNPRFSLLEPLKPMFGIGKSDQRFGYSSSVYCPCRASNEQFWWKQES

>Dr_LOC559025_PAPLNa_ADAMTS

MSKEKLDGLVKRMELTLAEMEQLKLHCGRQSEELSQLVVELRRENQELAEKYNQLAQDMKEAKEIIEQLQDTKYAFDAKERQAQKLTSEELKMLAVAVCLQLFLASVFMSSPQVAPPPPGLRSANVTLRAKRTSWLRRESPPQGASEELKMLAVAVCLQLFLASVFMQGIGDNWGEWSSYGECSRSCGGGVTMRTRRCVTHRTDGGHSCVGPDKSYRSCNIQDCPEGSRDFREEQCSQFDGTDFQGKRYKWLPYYGGENPCELNCIPRGENFYYRQKSSVVDGTPCRPGGKDICVDGLCKRLGCDHMLESPQEEDACLQCGGDGQSCSRLKNSFSMRNLPKGYNQMFIIPVGATTISIKETLPTRNYLAIKNLRGEYYLNGHWVIDLSRAASIAGTVLHYQRGREGEKSPEMITGRGPTTEPLVIEVRESSHCFTITQEVNEGVDYEYYLPNGGSTEGYYWSYGSWSACSKECGSGYQSRLVFCTFEDEAYSDYLCASLPRPLSNRTCNEQQCPQTRRMAYVYEAQLWRPTETPRVYTLAALEDLAALEDLAALDGLAALEDLAALEDLAALAALEVAVAALEVAVAALEVAVAALALEDWSRPSCELDDWSPPSCDLDDWSLPSRELDDWTPPSRELEDWTQPSWELGGVEFAILWTAGEWSPCPVTCGGGLQARRVECMSHDSTGSRLVEDSQCTAYTPRPLSQQNCNMQKCAQYSIYSWSQCSVTCGSGKQTRDVVCIGSDGASLEDYACGNQPKPPREQICEMPMCRSPIAWHIGDWGLVPSMQDPTGHDNTLHGFMPYSEDPLLALTPHCSQTHYGCCPDGRTPASGPQHQGCPQDQGQPSCTWSRYGCCHDGVTVAQGQNMEGCPDAWTHWSSSTSPTFYNRCMLSLGLLAQRSLSCDLPNTVGPCDQWTSRFYFDRSASRCLHFWYGGCHGNSNNFASEEECQKMCLRSELRQRDQAHVLSLQHGRRRFPAHVQLSSSGAHRAQRAHQPASNAMQQSNPAARKAKKGACRPCTETYIGMLDAYAFYMHKASDRFTNRVHVSVAISHDKGYVTAGRLCRTKRMPLMQKHEQQLDGSLLVGPITLQDSGWFLCVATRDDKRDHRYIYLSVSGQLYDVDEGVMV

>Dr_ZGC:110061_PAPLNb_ADAMTS

MMLHSLLGTLCLISAVFSLRPPSDDYYGEYGPYGPCSRTCGGGVAVRTRICNTMRTDGGHNCVGPSKSYKLCNTQECPAGSRDFREEQCSHFDQMTFQGKRYTWQPYYGASNPCELVCVPRGENFYYRHRPAVVDGTLCHVGRRDVCVEGVCRAVSHGEIVGFEDRDIPVTSRHGPGAAVHLDTYRYTYSAYSECSRLCGGGVQSRTVYCVHERTSAMVDESHCIAKGLRKPTAQVACNEHACAEYSAGPFGDCSVTCGEGLQTREVICVGGRGERLSEHHCSGLTRPQDTKACKRPACHQVFRYYTNDFSLCTRSCGTGTRERRVVCMDLDQHPYSDDRCASLSRPHAVENCNTQPCPGPQTVPSVQNPNGYESSLRGFLSYTHSPVSVYRPSDPYPTVTGPHCAQSHYGCCPDGHTTASGPRGEGCAHDDCQRSRYGCCLDGITAALGYRRAGCPDSSYGDHSPSASVCSLARDVGPCYEYKSRFYFDHSSGSCSQFWFGGCQGNGNNFVSKVACERTCKASVRGREPTSRRVIYDVRGYRVRSRA

>Dr_LOC556279_ADAMTSLa_ADAMTS

MACDSGHRWRLILHFLQMTLLLFFLCPPTEPAPWDSDSSSGPGEGCEGFQVDVCGVCAGDGSSCELFNGTIFLSVLSVRYHKILNIPSGALRIKIQETHKTRNYLALSMATGESVINGDWVIDRPGQFYAAGTQFTYKRPNEIRSRAGESITAPGPTNQELNLFVIYQQPDAAVYYEYILPKHTNTHSDAYSYSSVLPLVESHGIFPDSENSVGENSLSSFHPNQVPSDSVTLEPVPLYSWMAMATTPCSTTCGTGNRQVLFGCVERATQTTVLEDLCSSITHPGPQVEECQSQPCPAFWDVGEWSECSKTCGPGFQYRQVICRQTQGQHGSSTVVVANSMCDDTEMPDTTSVCQLKICSEWQIRSEWTECSVPCGVGQRSREVVCVDNLGDIVSDEECNMALRPQDLQNCDKGVCASSWFYSLWSERCSADCAEGSRSRSVVCLMSQSNSLPLDDCDDEDKPDELMQCDLGPCAQRLEWYTGPWGQCSSECGNGTQSRGVVCVVQNSGHLEVTSDDLCSHLPRPPTAQSCYLKSCAAQWFMTEWSSCSRSCEGGFRVREVRCLRDDLTSSHDCDPALEPAKQEKCNTHICTLQIDESCRDMYYNCVVVVQARLCVYSYYRTTCCASCSRVIQRDSLHTIR

>Dr_LOC556634_ADAMTSLb_ADAMTS

MGNTTAGPIRKSADPKACQKRAYCYNCSRKGHFGHQCSQRRMYNWSYPSLPVITYYDTVNDIKCRDFRLKKKAREMQDAGLISPDGGVVTFTPQPPRKKQKVSHSPHPGHRWRLILHFLQMTLLLFFLCPPTEPAPWDSDSSSGPGEGCEGFQVDVCGVCAGDGSSCELFNGTIFLSVLSVRYHKILDIPSGALRIKIQETHKTRNYLALSMATGESVINGDWVIDRPGQFYAAGTQFTYKRPNEIRSRAGESITAPGPTNQELNLFVIYQQPDAAVYYEYILPKHTNTHSDAYSYSSVLPLVESHGIFPDSENSVGENSLSSFHPNQVPSDSVTLEPVPLYSWMAMATTPCSTTCGTGNRQVLFGCVERATQTTVLEDLCSSITHPGPQVEECQSQPCPAFWDVGEWSECSKTCGPGFQYRQVICRQTQGQHGSSTVVVANSMCDDTEMPDTTSVCQLKICSEWQIRSEWTECSVPCGVGQRSREVVCVDNLGDIVSDEECNMALRPQDLQNCDKGVCASSWFYSLWSERCSADCAEGSRSRSVVCLMSQSNSLPLDDCDDEDKPDELMQCDLGPCTQRLEWYTGPWGQCSSECGNGTQSRGVVCVVQNGGHLEVTSDDLCSHLPRPPTAQSCYLKSCAAQWFMTEWSSCSRSCEGGFRVREVRCLRDDLTSSHDCDPALEPAKQEKCNTHICTLQIDESCRDMYYNCVVVVQARLCVYSYYRTTCCASCSRVIQRDSLHTIR

>Dr_LOC563685_ADAMTSLc_ADAMTS

VLLNDKYVVSGNGKPGLSNTYPSPLEKSLITYKLYLTPDHLPQTEELYISGPVTDKIHLQVYRKYGQEYGDLTNPNISYQYYSDDVIVEIASKGKWSTTISACSVTCGRGIQKIYQHCVDLVTLEHLKDELCSSLPRSSDLLQPCFLPDCPPRWQVSEPGECSTVCGPGEAQRKVFCVRFHNKSYLEVDQNLCSQPKPPEYVPCVVDVCPLKWDTENETQLIHRASHGMVRSKIVPVYVWSPRTGECSKTCGNGSQQVQFSCVDHKSRLEVPETLCDPTTKPAQDTQPCRISLCLPIWRYIQGPCSVSCGGGIAKRVLFCSQRMEENDGNNDLVVGETACDSVPKPPEVVECNTEKCKARPVHRAQTLTGAGAQRDQKVKSKCLLREALHLSQCGQRGSGSSHRATSLCMALPCDLPVIVTTVLDCLHTSVRTRVDLWLPGCLKKPAFGS

>Dr_LOC568792_ADAMTSLd_ADAMTS

MTSVSGAESRKPVRDICKALWCHRFGRKCETKFMPAAEGSACGPEMVSLRPNPNNTCHSHQVEPAHGGRFCEGSSRSYKLCNTDDCPANAVDFRAAQCAEFNSKPFRGWYYKWRPYTRVDEYTRVVTIPAGARSIRVSELNSSSSYLALRNLQRKYYLNGAWTVDWPGRHSIAGAIFDYKRPYNRPESLSSSGPTNQTLVIEVLLQGWNPGVQWEYTLNREQKHNYSWAVVRSQCSSSCAGGQMVSKSICYRDLRFQVNSSYCSARSRPSSGVMSCNTQPCPASWSVQDWSSCSRSCGSGQQVRRVVCVQKTGPDQLETVADAQCPQPAPAHTHSCNTHSCPPAWSAGAWTQCASHRVYCGVVVQCSVSCGEGLQQRVVKCAEKDYSGKYRELSQKKCQHVSRPSVDLQRVCVQAECPAAGTHWYSSPWSQCTVSCGGGVQIRSVQCLSLGRPSSGCVLQMKPLMSQACNTAFCPQPEHNDVVCKDHFSWCYLVPQHSVCNHKFYGKQCCKSCRQSKP

>Dr_LOC570564_ADAMTSLe_ADAMTS

PRSGGRFCVGRRMKFRSCNTDACPRGQRDFREEQCSQFNNRHFNINGLPASVRWIPKYSGSESAVISPHSPHVLLLFLISFSCIKGYNVVVRIPAGATNIDIKQVSYSGLPEDDNYLALSDSQSNFLLNGNFVVSMFKREISFKGSEIEYSGSNTTVERINCTQRIEEELVLQVLCVGNLYNPDVRYSFSIPIEEQREQFIWDPSGPWQECSRICQGENLNIKLDSKFLFDCVACSKTCGRGVRTRQAICMNNLGRRLVERECETQQKVLSEPCSDVPCPDWTASTWSECLVTCGKGVRHRQVQCILGDERLSEQRCDPNTKPSAVGSCTLPECAAWQTGAWSPCTVSCGAGYQMRAVRCVSGAYGETLEDRECNAAARPRDTQ

>Dr_LOC572470_ADAMTSLf_ADAMTS

SAPCAVTQLSKTSPWSPCSALYVTEFFDNGHGDCLLDAPETTVALPTELPGLTYSLDRQCQQIFGEEFSHCPNTSSSEVCERLWCQQEGQSMCTTRNGSLPWADGTSCGTNRTCLNSVCMSSEDVLRPQPAVDGGWGEWGSWQPCSRSCGGGVMFSYRECNRPSPQNGGKYCVGQRVNYQSCNKQACENNRVAGKSFREEQCEKYNNPNHFDIHGNVKQWIPKYAGVSLRDRCKLFCRARGSSEFRVFAPKVIDGTPCGPDTTSFCVQGQCIKAGCDLEIDSSKKLDKCAVCGGNGQSCRMISGSFNKVVHGYRDIVTIPSGATNINIKQQSHGSIPHDGHYLAVRRENGNYILNGNFSVSTVEQHIPVLGAVLKYSGSSTTLERLQSFRQLQEPITIQLLSTAAESIPPKVKYTFYIPKSMAFSKPKDKKIAGKLIHPFGVPQWISSEWSECSKTCGSGWSRRNVECKDNAGFYSNHCNKDLRPSDIRPCADLPCPIWQIGPWSSCSQTCGHGERQRKILCIDYTGKTVEPENCDPAKMPESVSEKCFYQEC

>Dr_BMP1A_BMP1a_BMP1/TLL

MELAARCALLLSSFSLLAALDLDAIEPGYYVETSSPSDLIDYKDPCKAVAYLGDIALDEEDMRMFTVDRIVNLAERTVTILNHTNTGSLSNETSVNTTASSRGSHRRRRAATSRPERVWPEGVIPYVISGNFSGSQRAIFRQAMRHWEKHTCVTFIERTTEESYIVFTYRPCGCCSYVGRRGGGPQAISIGKNCDKFGIVVHELGHVIGFWHEHTRPDRDEHVSIIRDNIQPGQEYNFLKMEPGEVDSLGEVYDFDSIMHYARNTFSRGIFLDTILPRYDVNGVRPPIGQRTRLSKGDIAQARKLYKCPRCGDSLQESSGNFSSPGYPNGYSAYMHCIWRISVTPGEKIILNFTSMDLYRSHLCWYDHVEIRDGYWRKAPLKGRFCGDKLPDPIISTDSRLWIEFRSSSNWVGKGFSAVYEAICGGEVKKDNGQIQSPNYPDDYRPNKVCVWKITVAQGYHVGLTFQSFEIERHDNCAYDYLEVRDGNSESSPLLGRFCGYDKPDDIKSSSNQLWMKFVSDGSVNKAGFAANFFKEMDECSRPDNGRCEQRCVNTLGSYKCACDPGYELAADKRSCEAACGGFITKLNGSITSPGWPKEYPPNKNCIWQLVAPTQYRITLLFDVFETEGNDVSTRFLPLRGVCKYDFVEVRSGLSADSRLHGKFCGAEKPEAITSQYNNMRIEFKSDNTVSKKGFKAQFFSDKDECSKENGGCQHECVNTFGSYSCQCRSGFVLHENKHDCKEAGCDHVVNSVSGTITSPNWPDKYPSKKACTWALSTTPGHRIKIAFNEIDMEPHLECAYDHIEIYDGRDSKAQSLGRYCGTKKPQPIISTGNKMFIRFFSDNSVQKKGFEASHTAECGGRLKAEVKTKDLYSHAQFGDNNYPGASDCQWVITAEKGYGVELIFQTFEIEEEADCGYDYMELFDGADTKSPRLGRYCGSGPPEEIYSAGDSIVIKFHSDDTINKKGFHVRYTSTKFQDTLHSRKK

>Dr_BMP1b_BMP1b_BMP1/TLL

MNSPWTRVLVLIFCLLTVDTIYQTGPGESDEVDYKDPCKAVAFLGDIALDEEDLRFLKAHYMHATENESSGSSKPDSVNRSSANRNAKDEPVLANQSILRRRRAATARPERVWPNGIIPYIISGNFSGSQRAIFKQAMRHWEKHTCVTFVERSVEESYIVFTLRPCGCCSFVGRRGGGPQAISIGKNCDKFGIVVHELGHVIGFWHEHTRPDRDEHVDIFRENIQPGQEYNFIKMEPDDVDSLGEVYDFDSIMHYARNTFSRGIYLDTMLPKYDVDGVRPPIGQRTRLSKGDIAQARKLYRCPRCGESLQDSAGNFSSPGFPNGYAAYLHCIWRISVTPGEKIVLNFTSMDLYRSNLCWYDYVEVRDGYGRSAPLKGRICGDQLPEAIVSTDSELWIEFRSTSNWVGKGFSAVYEAICGGEVRRDSGQIESPNYPDDYRPNKACIWKIVVPEGFHVGLVFQSFEIEKHDNCAYDYLEVRDGDSESSPLLGRFCGYEKPDDFKSSSNQLWMKFVSDGSVNKAGFAANFFKEIDECSRPDNAHCEQRCINTLGSFHCACDPGFELAPDRRSCAAACGGFISKLNGSFTSPGWPQEYPPNKNCVWQLIAPVQYRITLLFDAFEMEGNDVCKYDYVEVHSGLSADGKLHGKFCGTEKPEAITSQLNSMSVEFKSDNTVSKRGFKAQFFSDMDECSRENGGCQHECVNTFGSYSCQCRSGFVLHSNKHDCKEVGCDQAITSVSGLITSPNWPDKYPSKKACTWTLSTTPGHRIKLAFEEIDMEAHQECAYDHLEIYDGQNGRATSVGRFCGSKKPSPVISSGNTMFLRFFSDNSVQKRGFKASHSAECGGSLKAEIKTKDLYSHAQFGDNNYPGASDCQWVISAEKGYGVELIFHTFEIEEEADCGYDYVELFDGGDVKAPRLGRYCGSGPPEEIYSAGDAIVIKFHSDDTINKKGFHVRYTSTKFQDTLHTSK

>Dr_LOC558505_BMP1c_BMP1/TLL

MQQSFLQARCEETLVTSDWGIPLLVALLLLLLVYAFLYLPAISYRALLQQALDNHTEAMTVDNDKINIEDVVNYPGLPPRRQLTYQVKQLIARETELERMRRVERAQQSRNPQKMETVNKNQEEKKTEVKKQTSRNHQQRLENIVKQTVVETRPELDFFGRAIVPKEKPVVTATSEDGKVSGVLNIGKAVGNSDVWFRFNEAAACGGFISKLNGSFTSPGWPQEYPPNKNCVWQLIAPVQYRITLLFDAFEMEGNDVCKYDYVEVHSGLSADGKLHGKFCGTEKPEAITSQLNSMSVEFKSDNTVSKRGFKAQFFSDMDECSRENGGCQHECVNTFGSYSCQCRSGFVLHSNKHDCKEVGCDQAITSVSGLITSPNWPDKYPSKKACTWTLSTTPGHRIKLAFEEIDMEAHQECAYDHLEIYDGQNGRATSVGRFCGSKKPSPVISSSNTMFLRFFSDNSVQKRGFKASHSAECGGSLKAEIKTKDLYSHAQFGDNNYPGASDCQWVISAEKGYGVELIFHTFEIEEEADCGYDYVELFDGGDVKAPRLGRYCGSGPPEEIYSAGDAIVIKFHSDDTINKKGFHVRYTSTKFQDTLHTSK

>Dr_TLL1_BMP1/TLL

MDYLYSALTSKMNWIALLLAGLTFCCKVSVHSCLDYDDSYDYYEEEKTETIDYKDPCKAAVFWGDIALDDEDLKMFHIDGTIDLKQQTHGRQGHTSGGLGEHVPTKKRGSLYLLLDRIRRLGFESWPVNSSKDVSSIKTGIRRVNSARNVKSRVPRAATSRAEKIWPGGVIPYVIGGNFTGSQRAMLKQAMRHWEKQTCVTFIEKTDEESYIVFTYRPCGCCSYVGRRGNGPQAISIGKNCDKFGIVVHELGHVIGFWHEHTRPDRDDHVTIIRDNIQPGQEYNFIKMEPGDVNSLGEPYDFDSIMHYARNTFSRGMFLDTILPSRDENGVRPAIGQRTRLSKGDISQAKKLYRCPACGETLQDSVGNFSSPGYPNGYPSYTHCVWRISVTPGEKIVLNFTTMDLYKSSLCWYDYIEVRDGYWRKAPLLGRFCGDKIPEVLVSTDSRMWIEFRSSSNWVGKGFAAVYEAICGGEISKDSGQIQSPNYPDDYRPSKECVWRITVSEGYSVGLSFQVFEIERHDSCAYDYLEVRDGLSENSPLIGRFCGYDKPEDIRSTSNNLWMKFVSDGTVNKAGFAANFFKEEDECLKPDNGGCEQRCVNTLGSFKCACDPGYELAPDKKSCEAACGGLLTKLNGTITTPGWPKEYPPNKNCVWQVVAPTQYRISMQFEAFELEGNEVCKYDYVEVRSGLSSDSKLHGKYCGTEVPEVITSQYNNMRIEFKSDNTVSKKGFKAHFFSDKDECSKDNGGCQHECINTIGSYVCQCRNGFILHENKHDCKEAECEHKIHSTTGTISSPNWPDKYPSRKECTWDITATPGHRVKISFNEFEIEQHQECAYDHLEAFDGDSDKTPILSRLCGNKIPEPLISTGNKMYLRFISDASVQRKGFQATHSTECGGRLKAEARQKNLYSHAQFGDNNYPGHTDCEWLIVAESGYGIELTFTTFEVEEEADCGYDYIELYDGYDTGAHKIGRFCGSGPREELYSAGDAVLIHFHSDDTISKKGFHIRYTSTKFQEALHTRK

>Dr_MEP1A_MEP1Aa_Meprin

MLLQRLLIFAVLAAVLHAVPLSSRVHEVEDEPNFNPFINLGAKTRLIEGDIALPPGRIGLINTTYRWKFPIPYILSDSLDLNAKGAIYQAFEVYRLKSCVDFKPYEGEKTYIKFEKGDGCWSFVGDQQNGQVLSLGPGCDHKAVIEHELLHALGFYHMQSRQDRDDYVKIWLDQVIEGLEHNFNKYDDSFVTDLNTPYDYESVMHYRPFAFNKDPSIPTITTNIPEFYKIIGQYLDFSEMDIVRLNRMYNCSSSLTLLDQCAFEKINICGMVQSSTDDGDWVHLKSSEDHTLSGQCRDLGYTMHFDTSSGQAERSALIESRILYPKRKLQCLQFFYKMTGSAKDRLVIWARMDDGTGEVRKLKKLQTIWADEDKTWKIAHVPMQVGAKFRYAFQAVKGDSSSSGGGIFIDDISLTETHCPAAVWRIQNFSSILEKADYSTVLNSPRFYSPEGYGFGIQVIPLSGYSDYAGNYTGLYFHLISGDNDIVMQWPAVNRQATIVVMDQDPDIRLRMSSARSLTTDLSKGNGEQLLWDNPKKVGTLDPSDGFWRGPSKGWNTFIKHYDLHRRNYLKNDDLIIFVDFEDLTSLIKSEVPTAPKV

>Dr_LOC567040_MEP1Ab_Meprin

MLLQRLLIFAVLAAVLHAVPLSSRVHEVEDEPNFNPFINLGAKTRLIEGDIALPPGRIGLINTTYRWKFPIPYILSDSLDLNAKGAIYQAFEVYRLKSCVDFKPYEGEKTYIKFEKGDGCWSFVGDQQNGQVLSLGPGCDHKAVIEHELLHALGFYHMQSRQDRDDYVKIWLDQVIEGLEHNFNKYDDSFVTDLNTPYDYESVMHYRPFAFNKDPSIPTITTNIPEFYKIIGQYLDFSEMDIVRLNRMYNCSSSLTLLDQCAFEKINICGMVQSSTDDGDWVHLKSSEDHTLSGQCRDLGYTMHFDTSSGQAERSALIESRILYPKRKLQCLQFFYKMTGSAKDRLVVWARMDDGTGEVRKLKKLQTIWADEDKTWKIAHVPMQVGAKFRYAFQAVKGDSSSSGGGIFIDDISLTETHCPAAVWRIQNFSSILEKADYSTVLNSPRFYSPEGYGFGIQVIPLSGYSDYAGNYTGLYFHLISGDNDIVMQWPAVNRQATIVVMDQDPDIRLRMSSARSLTTDLSKASSALNADMRWNKPSTFEQWDDSCLCFRGPEFGWGTFISHDQLRRRDFLKNDDLIITINFDACRKNIEKPKIRQPRAISDLCQPNPCQNGGACVTHQGKATCRCASGQADVYTGDTCEKQHIDGGIMGVLIGGAVGTVALTVAIIAVIYRQK

>Dr_DKEY30J22.6_MEP1Ac_Meprin

MLGEDADADSQRELFEGDIAGDPRRNAIIDEKARWQFPIPYILTDTLDLNAKGVILQALEMYRLKSCVDFKPYEGESTYISFTKLDGCWSFVGDLKTGQNVSIGERCDTKAIVEHELLHALGFYHEQSRSDRDDYVKIWWDQIIEGKEHNFNKYEDDFITDLNTPYDYESIMHYRPLSFNKDPDIPTITTTIPAFNNIIGQRLDFSALDLERLNRMYECTATHTLLDQCAFEQINICGMIQNDEDDADWVQTLSSTDLKDHTLGGQCRDSGYFMKFDTDNKTEGHSALLESRILYPKRNQQCLEFFYRMSGEPGDKLIIWVRTDDGTGNVNKVRKVHTITGHGDNSWNIAQVTLNVKEKFRYFFQGIVGPNKTSGIFIDDIILTETSCPNTVWRIQNFTNLLNTLPHDAKVQSERFYNSEGYAYGINVYPNGRVNSSKEFVGITFNLFGGENDAVLEWPAVNRQVTVTAKDQNPDATLQMSNSRSFTTDADMRWNKPSTFEQWDDSCLCFRGPEFGWGTFISHDQLRRRDFLKNDDLIITINFDDLAHLVKSEVPTKVSNNPQPAEKNIEKPKIRQPRAISDLCQPNPCQNGGACVTHQGKATCRCASGQAVVYTGDTCEKQHIDGGIMGVLIGGAVGTVALTVAIIAVIYRQ

>Dr_CH211191A24.6_MEP1B_Meprin

MASACSYLFLSVCATVLCLPTSSVTGDTEIDIDHGTDLDIFEINEVAGLDLVEGDILIEEGESRNTILGEQYRWPTTVPYFLDNSLEINAKGVILKAFEQYRLKTCIDFKPWNGESNYIFVFKGSGCYSKVGNRQMGKQELSIGSNCDSLGTVEHEFLHALGLWHEQSRSDRDDYVIIVWDQIQDGKEHNFNLYDETQSSSLGVPYDYGSVMHYSKTSFNKGSEPTIVTKIPEFLNVIGQRMEFSDNDLLKLNRLYNCTTSSTFLDSCHFEEPNICGMIQGDGGNAKWTRVQTVEGGPNTDYTNLGQCQGVGFFMHFSTATEAQGDKAHLESRLFYPNRRSQCLQFYHYNSGGTDDQLNIWVREYTAENPKGDLRLIQQISGGLKDSWELYHVTLDVSSKFRVVFEGVKVRDTSKGGLSLDDINLSETQCPQHSWRIRDFTKLLATTAPGSKIYSPRLLSPDGYSFQIGLYINGLKDSPDKMAIYLHLTSGPHDDNLQWPCPWRQASMEMMDQNPDIQRRMNNIRMITTDPDKTSTDSSGNVEYFWDDPRKVGSRVTDTDGSTFYRGSGYGTSSFITHDRLKSRSFIKGDDVIFLLSLEDVTGLLEQQSREFQSPEFVDLRLEMQESIGSSTATVAISVFVAAAMFLGIVVSAMIYVQRRRRRQERDTEMEPERTDGYLILKCVIH

>Dr_mmp2_MMP2_MMP

MLSVKFFRCRHIVLKVFLVQFLASLQTFAAPSPIIKFPGDDTAHTDKEVALHYLNKFYGCPKDRCNLMVLKDTLKKMQKFFALPETGEIDQKTVEIMKKPRCGVPDVANYNFFHRKPKWGQKNVTYRILGHTPDLDEDTIDDAFYRAFKVWSDVTPLKFTRIMDGEADIMINFGRNEHGDGYPFDGKDGLLAHAFAPGPGIGGDSHFDDDEQWTLGEGQVVKVKYGNAEGEFCKFPFLFMGKEYNSCTSQGRDDGFLWCSTTYNFDDDGKYGFCPHELLFTLGGNADGAPCKFPFTFQGDKYDSCTTSGRDDGYRWCATTEDYDKDKTYGFCPETAMSTSGGNSDGAPCVFPFKFLGDSYDSCTTSGRNDGKMWCAVTKSFDDDRKWGFCPDQGYSLFLVAAHEFGHALGLEHSDDPGALMAPIYTFTKTLRLSDDDVKGIQELYGEPTDKPLATHTPPVTPMDVCNENIIFDAVAQIRGEIFFFKDRFLFRTADVRKKPTGPMLVATFWSELPEKIDAAYENPLEERTVFFAGDEMWVYSASTLEREYPKKISSMGLPSDLHGIDAAYSFHKTKKTYIFAGNKFWRYNEAKKKMDPGFPKIIADSWTAVPDDLDGALSLNGDGHSYFFKDSHYLKMDDSTLKIIKVGEVKKDWLRC

>Dr_LOC570150_MMP7_MMP

NGGLVFLYHPCVSAEARRSLAVLAHSCLSHYILTPHPWLSQHRPLAVVSWGRSLEMSQITLRVCDWLLSIFPNITLFSTSHGVKYNMYLTKPALHKPVNTSQEMSRVERLKSLKHCCMRILSLEHRTRKTRMALKQSQEDAELKENVKLSSTDSAKLNHTDTLPQNNTIQTAIQSHGSAKEPERTATNHTKTTEQQLNIVTERQKYQTNRKSHKKTDSRKHRIKTDTKAQTSESECREFAQCGVPDSNHIEGSIRGERISIPRTDEAVWAAGAVGFILVLLTLSVLHTRLYRHCRPSTSLYWHDNQQDYDSVGAKTFNVSLDWPFPEASVVCREEMQVLLCLVCAFDLFGQHYSLPLPLGDDGMVHPTKPAQNDLSLATKYLQQFYSFQADSAGRKRRSRPSFSSKLKDMQSFFGLNGTGTLNADTLAVMRTPRCGVSDVEDYSHRRGNRWKKNIITYGVGRYTNDLPVNTVDDLISSALDVWAKASPLTFLRSYSHQADIMVEFVGKEHGDFFPFDGPDGTLAHAFGPGEGIGGDVHFDEAEVWTAGYNGFNLYVVAAHEFGHALGLKHSQLPESVMYPTYKRRKTHNLLSSEDITNINTLYGPRDKRPYPSSRFSWSNPSTPWYSGSYFPVSFKDTCNPHLKFDAVTTVGDAIFFFRDK

>Dr_mmp9_MMP9_MMP

MRLGVLAFLVLGTCSLRAWCLPLKSVFVTFPGDVIKNMTNTQLADEYLKRYGYVDVLQRSGLQAVISNAKALKKLQRQLGLEETGLLDQPTVDAMKQPRCGVPDIRNYKTFDGDLKWDHTDVTYRILNYSPDMEASLIDDAFARAFKVWSDVTPLTFTRLFDGIADIMISFGKLDHGDPYPFDGKDGLLAHAYPPGEGTQGDAHFDDDEYWTLGSGPAIQTRYGNAEGAMCHFPFLFEGTSYSTCTTEGRTDGLPWCSTTADYDKDKKFGFCPSELLFTFDGNSNEAPCVFPFVFDGKKYDSCTTEGRNDGYRWCSTTANFDTDKKYGFCPNRDTAVIGGNSEGEPCHFPFTFLGNTYSSCTSEGRNDGKLWCGTTSNYDTDKKWGFCPDRGYSLFLVAAHEFGHALGLDHSNIKDALMYPMYKYVEGFPLHRDDIDGIQYLYGPRTGPEPTAPQPRTTTSSPVVPTKPSPSDKTTTASTTTQVVPSDDACQIKEFDAITEIQKELHFFKDGRYWKISGNGERKGPFMISAKWPALPAVINSAFEDHLTKKIYFFSERQFWVYSGNDVLGPRKIEKLGLPSDLDKVEGSMQRGKGKVLLFNGENFWRLDVKAQLIDRGYPRFTDAAFGGVPIDSHDVFLYKGFFYFCRESFYWRMNAKRQVDRVGYVKYDLLKCSDIHSL

>Dr_LOC563753_MMP11a_MMP

MEPAEYEAIMELAKYETMVKLAEQECSVEPAEPESRTGAGQRTKKSLHINKCSLCSSSSTAGITHASTSHFTFSGGARGYRRRMRVSGLLACAFALHVLLTARCLPLHGGQGLAKHKESPGLSYIPHLHSEKRRGRVPHPQDTFKPPAWPKEEPHINTPLRNSSRATGPKRCGVPDYPEQRDVHLRQKRYVLFGGRWPKTDLTYKIQRFPWQMREDKVRRIFQEALKVWSDVTPLTFTEVVNQEADIVIDFTRYWHGDNLPFDGPGGILAHAFFPRTHREGDIHFDYDESWTVGNELGTDLLQVAAHEFGHVLGLQHSLVPGAIMSPFYSFSYPLKLSEDDKKGIQYLYGPPVRAQPQIPAETNDIPSAFPPDACHTDFDAVSIIRGELFFFKASYAWRIRDGRLQAGYPALASRHWSGIPQKIDAAYEDKKGNIWFFEGSNYWVFDAEHRIKGPDSLLSLGLRVSNIQAALRVKEHHSQHTYFFKSGNYWRLDPQENRVDTSAPRRIQQDWWGVPEEIDAAFQEASGFVVFISRRQYWKFDPVQRKVLEGYPRYIGADFFGSRRPLSPRQGEHNLRGPSGKHGRPVVGPRCEGYARRPLSPRQGEHNLRGPSGKHGRPVVGPRCEGYGGWE

>Dr_LOC565793_MMP11b_MMP

MSSSVAGVFVNKRKSSSRSAIVGMHRELLSAREITNHEAFCNVLEDEGVMAGLENLELLNWIYTSIICNFKTFQKYFISSLGTDLLQVATHEIGHVLGLQHSKVPGAVMAPFYTFSYPVRLSEDDKRGIQALYGSKRSDETVKAAERRPTFTERNEIDATFFPPVHPNPSHPDACQTNFDAVSMIRGELFFFKSGYVWRIRDGKLQAGYPALASRHWRGIPDNIDAAFEDMSGNIWFFQGQNYWVFDAERQITGPDSVQRLGLLVNDIQAALMWGDTKAQKIYFFKKGSYWRFNLKENRVESMHPRSMSDWRGIPSDIDAAFQDRFGFAHFLRGKQYWKFDPVEVRVLEGYPRYIGVDFFGCSAALYR

>Dr_MMP14A_MMP14a_MMP

MLPKLQTLPRLLPLALASVFLVQSGTSDKEVRPEAWLQQYGYLPPGDVRAQAIRSPKSINSAISAMQKFYGLTVTGTMDPATLSAMQRPRCGVPDKFGSELKSNLRKKRYVAQGSKWDKREVTFSIQNYTPKVGERATHEAIKKAFRVWEAVTPLKFREIPYSQINGKVEKFADIMLFFAEGFHGDSTPFDGEGGFLAHAYFPGHGIGGDTHFDEAEPWTTGNVDKGGNDVFLVAVHELGHALGLEHSGDPSAIMAPFYQWMDTENFVLPEDDRRGIQQIYGAGSEDKPQPPAPRPPTRTPDRPSFGPDICEGHFDTIAFLRGEMFVFKEKWFWRVRDGKPQQGYPMPIGHFWKGLPPSINAAYERNDGKFVFFKGDKYWVFNEAKMEEGYPKTFKELGTGLPRDKLDAAIFYTPTGNTYFFRGTKYYRFNEESRSVDSDYPKDIGVWQGVPDNVKGAFMSEDGANAYFYKANKYWKFNNQQLKVEPGFPKSVLTNWMGCEAEEPKRRAGTDEEVLIIEVDGSEGGAMGGAAAIVIPLFLLACVLVTLGALLFFRRYGTPRRLLYCHRSLLDKV

>Dr_MMP14B_MMP4b_MMP

MIWSGFTRLLLLIFVCAHRSSSKQDMKPEAWLQQYGYLPPGDLRTHTARSPQSVPSAIAAMQRFYGLTVTGNLDANTLEAMKRPRCGVPDKFGSELKSNLRKKRYAIQGLKWDKNEITFCIQNYTPKVGEYETFEAIRKAFKVWESVTPLRFREISYSDIRDKVVDFADIMLFFADGFHGDASPFDGEGGFLAHAYFPGNGIGGDTHFDAAEPWTIGNKDLLGNDVFLVAVHELGHALGMEHSNDPSAIMAPFYQWMETDHFVLPDDDRKGIQKLYGPGSGGHPRPPVSPETPHHTPYPTPYRPGGPSYGPNICEGHFDTIGIFRGEMFVFKGKWFWRVRNNQVMENYPMPIGHFWRGLPTDINAAYEREDGKFVFFKGDRHWVFTESNLEPGYPKVLGELGTGVPKDKLDAALLYTPTGYTYFFRGNKYYRYNEDTHSVDPDYPKPISKWQGVPDNIKAAFMSRDQGYTYFYKANKYWKFNNQLLKVEPGYPKSALKDWMGCPNEDSNTGGGGSDRDRERERERERERERERERAREREREQDRTNEVDKTEEEGKKEETEVLIIEVEDAPSSRGGAAAVVVPLMLLVCVIFTLGALLFFRRYGTPRRLLYCQRSLLDKV

>Dr_LOC566945_MMP14c_MMP

MIWSGFTRLLLLIFVCAHRSSSKQDMKPEAWLQQYGYLPPGDLRTHTARSPQSVPSAIAAMQRFYGLTVTGNLDANTLEAMKRPRCGVPDKFGSELKSNLRKKRYAIQGLKWDKNEITFCIQNYTPKVGEYETFEAIRKAFKVWESVTPLRFREISYSDIRDKVVDFADIMLFFADGFHGDASPFDGEGGFLAHAYFPGNGIGGDTHFDAAEPWTIGNKDLLGNDVFLVAVHELGHALGMEHSNDPSAIMAPFYQWMETDHFVLPDDDRKGIQKLYGPGSGGHPRTPVTPETPHHTPYPTPYRPGGPSYGPNICEGHFDTIGIFRGEMFVFKGKWFWRVRNNQVMENYPMPIGHFWRGLPTDINAAYEREDGKFVFFKGDRHWVFTESNLEPGYPKVLGELGSGVPKDKLDAALLYTPTGYTYFFRGNKYYRYNEDTHSVDPDYPKPISKWQGVPDNIKAAFMSRDQGYTYFYKANKYWKFNNQLLKVEPGYPKSALKDWMGCPNEDSNTGGGGSDRDRERERERERERERERERAREREREQDRTNEVDKTEEEGKKEETEVLIIEVEDAPSSRGGAAAVVVPLMLLVCVIFTLGALLFFRRYGTPRRLLYCQRSLLDKV

>Dr_LOC561041_MMP15a_MMP

MSVCRSQSLRAVSVIFILLFVNIAETSAEDEDFNAESWLRTYGYLSQASRQMSTMQSSQILSSAIRDMQRFYGLQETGHMDSETLRAMKRPRCGVADHFEESSEGAARRKRFALTGHKWNQNNLTYSIQNHSPKVGQQQTYEAIRKAFRVWEKVTPLQFEEVPYHKIKNGSEGPDIILLFASGYHGDMSLFDGEGGSLAHAFFPGPGMGGDTHFDTDEPWTLNQQEGSGVDLFLVAVHELGHALGLEHSNNPSAIMAPFYQWMDTESFALADDDINGIHQIYGSPETVTTQAAPTTTLFTTTAKPEPTTTEAQPKTTRPSLQPTRPWVPPVNPTRRTYRPQPTSRSNQDAPDICEGNFDTVTMLRGEMFVFKGRWFWRVRRNRVLDNYPMPISFFWMGLPEDIDAAYERHDGKFVFFKGSKYWLFREADVEPGYPQDLFRYGQGMPERVDTAVWWEPSGYTYFFRGDRYWRFSEESRAMDKDYPKPVSVWGSIPVSPKGAFLSDDGAYTYFYKGTKYWRFDNKRMKVDSGYPRSILNDFMGCRVHFDVEPEIYPDHQSPDMTDNNPDPEDYKNGVDEEDPEDDDVDEKKEVDVILRVNDNDKHIMTLILVIVPLVLVLCILGVIYVIITTLQRKETPKVLVHCKRSLQQWV

>Dr_LOC562086_MMP15b_MMP

MVLAEDVRLPVSGQQTDVHYAVFTDPLKCHQDMQRFYGLQETGHMDSETLRAMKRPRCGVADHFEESSEGAARRKRFALTGHKWNQNNLTYSIQNHSPKVGQQQTYEAIRKAFRVWEKVTPLQFEEVPYHKIKNGSEGPDIILLFASGYHGDMSLFDGEGGSLAHAFFPGPGMGGDTHFDTDEPWTLNQQEGSGVDLFLVAVHELGHALGLEHSNNPSAIMAPFYQWMDTESFALADDDINGIHQIYGKLPKTTRPSLQPTRPWMPPVNPTRRTYRPQPTSRSNQDAPDICEGNFDTVTMLRGEMFVFKGRWFWRVRRNRVLDNYPMPISFFWMGLPEDIDAAYERHDGKFVFFK

>Dr_LOC562281_MMP15c_MMP

MATLLAPDGMYFPYEEDNHRLVREIGNVTEAVEITFQFAVRPERVETVCQADDGGQSAGGTETTDSPARPSEGHQSWLRMYGYLPQASRQMSTMRSAQILSNAISDMQRFYGLEITGEMDPGTIEAMKRPRCGVPDKFGAQIKTNVRRKRYALTGHKWDKTHITFSIQNYTPKIGEYNSYEAIRRAFRVWEKVTPLTFDEIPFHEVKYGRRKEPDIMIFFASGFHGDSSPFDGEGGFLAHAYFPGPGMGGDTHFDSDEPWTIGSQNLQGNDLFLVAVHELGHALGLEHSNNPLAIMAPFYQWMDTENFELPEDDLRGVQQIYGPPSSSPTQALPTVTPRRPAHPDPRAPNPPKSPPGAPPRRPDKPRTTDRPDHYGPNICEGNFDTVTMLRGEMFVFKGRWFWRVRRNRVLDNYPMPIGHFWRGLPGDIDAAYERHDGRFVFFKGSQYWLFREANLESGYPQELMDYGRDIPYDKIDTAIWWEPSGFTYFFKGDWYWRFNEQDRAADQDYPKPISVWGTSVPSSPKGAFLSDDGAYTYFYKGAKYWKFDNHRMTSEPGYPKSILRDFMGCNVDLDPDRDTDVDQGRKWPDRPPFNPDAGRDKDKDKDKDKERDNTNDADSKEETEEKTNEVDVVLKIDETERTMNIIMVTVPLVLVLCILGLIYAIINTLQRKGAPKYLVYCRRSFQDWV

>Dr_LOC571926_MMP16a_MMP

MLNPQMGKQNLLEAFWLLCEGSRRSIFKSAPKSSFLNDGADAELTASQMISRRESYMERQKKRGIATSSVSLRRSRDAEEEKQAQMAAKMCCTETLSLKGDRGEQLAFKTSCSSLKVRELSYPLQENRKDALLWTRTDWLQTYGYLPPTDPRMSILRSEQTMQSAIAAMQRLYGLKVTGELDKNTIDWMKKPRCGVPDQFDRASKFSVRKRRYALTGQKWLHRHITYSIKNFTPKVGAEETHNAIRRAFDVWQNVTPLAFRGSAVQRTGAQQERCGYYYYFPLKVFTGDRLPLSTEKGGFSCHMPYFPGPGIGGDTHFDSDEPWTLGNPNHDGNDLFLVAVHELGHALGLEHSNDPTAIMAPFYQYMDTENFRLPHDDLQGIQKIY

>Dr_LOC572034_MMP16b_MMP

WMKKPRCGVPDQLKAGSASRRKKRYALTGQKWQREHISYSIKNVTPKVGNQETHDAIRRAFDVWQGVTPLRFEAVPFRDLDSGKRDVDITIIFASGFHGDSSPFDGEGGFLAHAYFPGPGIGGDTHFDSDEPWTLGNPNHD

>Dr_LOC570076_MMP17_MMP

MGNKNGDEQTQSELPGMSGGERARIRVRCSDLGRGQAARIRVLITGVVRGQAERVRVCITSVGRGQAEGCLICEKVHQAPPDVLCKPDDSVELTCSHNIDNYDTILWYQKSKGDSSLKLIGYARYSSAKDIEKSYMGHFNVTGNGEEEATLRILKARLDQDTCVLCKDVHQSPPAVVCDTDSPSTVNLTCSHGIKSYDTILWYQSNVGDTEMKLVAYMYYKTPKTEDPFVNGFDVSGVVFSDKVHQSPNAILWSPEGRAEINCRHDISTYDTLQWYQQTDNTLKLIGYVYYEQRNIENAFEKRFNVSGHGKTHSTLYLLQLRSPEDSAVYYCAAYYHMNSSIIQTPSDLLANNTESVSLHCSHSSSAFNVILWYKQLLNGEMQLMGHLYRTNNNTETLFTDKIELLGNGASSSILSMAVNVIQTPTELFLDTGATLNITCSHNDRSLDKIFWYQQFNGRNLELIGFLSFKQRLVDKKEFTITGDAEKEATLVLSSVKAEHSAVYFCAQSPKELLTVEVKDTNLSCHHGESSYPYMLWYQQKSNGGSLELIGLLTFGSFSPEDKFKSRFSTFGDSTKEAFLLISKDWLSKFGYLPPSDPVNGQLQTKEALTKAIKAMQRFGGLEETGVLDQATLGLMKTPRCSLPDMSEPDLSAGRRKRALAPQNKWNKRHLSWRVRTFPKESTSLGRDTVRALMYYALKVWSDIAPLNFHEVAGNEADIQIDFTKADHNDGYPFDGPGGTVAHAFFPGERFTAGDTHFDDDEAWTFRAPDSHGMDLFAVAVHEFGHAIGLAHTSAIESIMRPYYQGPVGDPLKYNLAYEDKVRVWQLYGVRDSVSHTLKPDDPSQTAEPPVLLDLPENRSTIPPARDAPDRCTSHFDAVAQIRGEAFFFKGKYFWRLTREKHLVSLRPAQIHRFWRGLPANLDSVDAVYERPGDHKIVFFKGGKYWVFKDNNVEEGYPRSVSDFGLPVEGVDAVFVWQHNEKTYFFKDNRYWRYDDHLRRMDLGYPKDMALWKGIPAQLDDAMRWSDSASYFFKGKEYWRVAGSDMEVEVGYPRPIGKDWLVCTEMQSDSPEMQNNSNTRLHGQHHADHAENGYEVCSCTSDSASPLGTRLTLSPAWVFAPLLTLALALSSAPL

>Dr_ZGC110623_MMP23a_MMP

MFWVCSCALVLMVLDSIAVAVPAWTEHTVGSFQLRKPCAQKLSSVDSKTHGGAARSKRYAINPLGYRWEHFNVSYKITKFPNTLNKDDTRKAISIAFTKWSDVSPLTFTEITNTSKSADITIGFYTYNHTDCWRSPLHPCFDGLNGELAHAFLPPRGEIHFDNHEFWILGKSRFSWKQGVWLNDLVQVAAHEIGHALGLWHSQDPNALMHPNATYTGQRNIAQDDIWGIQRLYGCMDKKRVCDPWARLGFCERRRSFMKKNCPQRCDLCYEPLDAVSTPTPPPENVKIKIVPRGKVVGFRCGTKSTRVPPKVSWYKDGEQLLTSIPGYIVIKDRDLRLVANEFNEGTYTCRIHRRGTIVSANSWAIRPKPEQSSNNS

>Dr_LOC568293_MMP23b_MMP

MASSGYTAEDLQPSQTQGGSITNATSSSQQKNTSQILSDIQNPQGSASSIIRAQATPPATQKKAVLATPSPGVQYVTGEIQSSASQTGNGSQQYIVVTVTEGSLNSNDSESNSPPAVQTGVPTQVVQQVQTAQYIKAFYNQTWMSRYNEPIGEGTPMEQSSLKNSDVLILTGLTQIPTANPDGMLGEFCSNLAMTVRAGGNVLVPCYSSGVIYDLLECLYQFMDSANLGTTPFYFISPVANSSLEFSQIFAEWLCQNKQSKVYLPEPPFPHAEEEALMSGVLMIGIHKDARSHVLHLSRNKRYTLTPEKLKWDNFKMTYKLLSFPRNLLNATDTRRGISKAFSMWSDVSPFSFREVPSDQEADIKIGVWLTDLVHVAAHEIGHVLGLMHSQNPKALMHLNATLTGRKLITQDEVWGLHRLYGCLDRFFICPSWARKGYCDSKRKLMQKHCPSSCDFCYGETPRIHY

>Dr_LOC571143_MMP24a_MMP

METWLKNYGYLLPHDIRTSDLRSEKAMQSAVAAMQRFYGIPVTGILDQTTIEWMRKPRCGVPDHPHISRRRRNKRYALTGQKWRDKKITYSIHNFTPKVGEKDTQRAIRQAFDVWQTVTPLSFQEVAYSEIKNEGKEADIMIFFASGFHGDSSPFDGEGGFLAHAYFPGPGIGGDTHFDSDEPWTLGNANHDGNDLFLVAVHELGHALGLEHSSDPSAIMAPFYQYMETHNFKLPLDDLQGIQKIYGIPTAMLEPTRLFPTFPARRPHSTSERKHERQSRPGRPSAGDRPSSPGHGKPNICDGNFNTVAFFRREMFVFKDRWFWRLRNNKVQEGYPMLIDHFWKGLPHRIDAAYERSDGKFVFFKGDKYWIFKEVTAEPGYPHSLVELGSYLPSDGIDTALRWESVGKTYFFKGDQYWRYNEEKRTTDPGYPKPIGIWKGIPDAPQGAFVSREGFYTYFYKGKDYWKFDNQKLTVEPGYPKSILKDWMGCDQSEVEKNKDRHLPHDDVDIMVAINDVPSTVNAIAVVIPCILSLCILVLVYTIFQFKNKNVQQHVTYYKHPVQEWV

>Dr_ZGC239j9.5_MMP24b_MMP

MESAGKEMNEKGRVGQESVIDESEGVNKRQTVRTNESPGNDLFLVAVHELGHALGLEHSSDPSAIMAPFYQYMETHNFKLPLDDLQGIQKIYGIPTAMLEPTRPLPTFPARRPHSTSERKHERQSRPGRPSAGDRPSSPGHGKPNICDGNFNTVAFFRREMFVFKDRWFWRLRNNKVQEGYPMLIDHFWKGLPHRIDAAYERSDGKFVFFKGDKYWIFKEVTAEPGYPHSLVELGSYLPSDGIDTALRWESVGKTYFFKGDQYWRYNEEKRTTDPGYPKPIGIWKGIPDAPQGAFVSREGFYTYFYKGKDYWKFDNQKLTVEPGYPKSILKDWMGCDQSEVEKNKDRHLPHDDVDIMVAINDVPSTVNAIAVVIPCILSLCILVLVYTIFQFKNKNVQQHVTYYKHPVQEWV

>Dr_LOC556862_MMPLa_MMP

MPDQYARGVDWLIRYGYLQSPDTLIGGLQTKESIEEAVRKMQRFAGIEETGNLDQKTLEMMGRPRCSLPDTISFEELLKRNRRGKNKVMMKKKRYTLPKMRWDKTDITWSMQDFPPPSVSPALNPGLVRLILGNALRVWSENTPLRFHYSPDEPSPQTDITVTFTSGYHEDGYPFDGKGGALAHAFFPGKGDLAGDTHFDAEESWTYGDWSSDSDLFTVAVHEFGHALGLFHSSSSDSIMKPYYFSPVGEMNSYSLTAVDRLGIQALYDSADRCQGGYDAVANIRAEIFFFRGQHFWRVDHSGSLLSSTPALIHSFWIGLPPDTARVDAAYERRDGHIVFFIGNQYWVFRNTMALPGYPRQLSDWGLHTAAGQLAESVEAVFVWPHNGKTYVFSSGLYWRFDEAGGERKMEEGYPKPAAIWGMPSHPDDIIAFLDGETYFFKDSNYWILQRGGLDLESASPKSIATDWMKCDRHVSAPTPEIPRDRNCSCVQNVAAVMRSLSGFYKQPLSSTSVQH

>Dr_LOC563014_MMPLb_MMP

MSFSGYLGLVYLTCPMLVFFSAVHSAPVKDQYSRGVDWLSRYGYLPPLDPRTGQLQSKDGIERAIREMQRFAGLKETGKLDSDTLTLMNTPRCSLPDIIGIEDKLKKRRRKRYATTGLRWTKSDITWSVQNYPSVHKRLTPTQVDPIISYALKAWSDVTNLNFYGASSSEKDRADIRISFARSLHDDGYPFDGKGGTLAHAFFPGESDVAGDTHFDDDEIWTYLDESGTDLFAVAVHEFGHALGLSHSSSSPSIMKPYYQGSVGDVGSYILPDDDREAIQSLYGRKISSPTPSPNKPTSHLPKPPATYPKGPVKPDPSIQNRCEGGFDAVANLRGEVFFFKGPYFWRIQRTGSLVSFQPAHIKNFWMGLPPTTNKVDAVYERKVDNAIIFFIGSQYWVFKNTEVLPGYPRPLSDWGMITQDGRKVMRVDAAFIWAHNGKTYIFSGGEFWRFSEGRETELRGPDTGYPRNTNLWKGAPSNPDDVITWGQETPISSRTIHTGC

>Dr_LOC557327_MMPLc_MMP

MLEEQKGGGKDWLTKYGYLPPPDPSTGQLQAWTAVTQAVKKMQSFAGLDETGILDEETLQLMQTPRCSLPDDDDDDQTIHSAQHADPQNQRMKRAVSTWTRRNINWRLRSYPVSSKLSRETIRSLVFYALRVWADPTLLEFHEVRGPEGADLQIDFLHGPHGDGYPFDGAGGSVGHAFFPSDPNRAGGVHLDAEEDWAFRQPATEGTDLFTVLVHELGHALGLTHSSARRSVDRCNTSFDAVAKIRGEIFFFKGQSMWRISRGGLVSVRVVPIQRLWSALPPSLPPLRAVLERHTDHAIIFISGSTYIFKGNSYWKFTHPGSAPVEGYPRLSASDWLDCPQDSSYSSADISLISQFGQQELHEQKKQTIINQIRHSEINKPQHWDCPCQNTAGTQRGHVLLLAFLLLLTVTC

>Dr_LOC564525_MMPLd_MMP

MMLMFWTAVVFCLWGSAVDVFGEANEDALPTSLMNMVTSTILPTAIPTEDESGKLVDWLTKYGYLPPPDPSTGQLQAWTAVTQAVKKMQSFAGLDETGILDEETLQLMQTPRCSLPDDDDDDQTIHSAQHADPQNQRMKRAVSTWTRRNINWRLRSYPVSSKLSRETIRSLVFYALRVWADPTLLEFHEVRGPEGADLQIDFLHGPHGDGYPFDGAGGSVGHAFFPSDPNRAGGVHLDAEEDWAFRQPATEGTDLFTVLVHELGHALGLTHSSARRSVMRPYYQGPLGDPLHFSLGYQDLQHITALYGQRGNDIPTETPLPVTETQLRHRHHRLTHMYSSVDRCNTSFDAVAKIRGEIFFFKGQSMWRISRGGLVSVRVVPIQRLWSALPPSLPPLRAVLERHTDHAIIFISGSTYIFKGNSYWKFTHPGSAPVEGYPRLSASDWLDCPQDSSYSSADISLISQFGQQELHEQKKQTIINQIRHSEINKPQHWDCPCQNTAGTQRGCCIIFVHHISAIMSAFVVLAALGLLGTLPDCDELTKPQSLEDDYKSIMGKWIFHKGIADHQLFTQILKTVNSSWIEFGPSPLENTVTLSQGNMLNGKCEFSTTNAAIKDNTIYSDHNGTLSEGKFLLSCPDCLTISFVSQFKNETIKTLYFF

>Dr_LOC565762_MMPLe_MMP

MTGFKSSNEWFQKANHNDGSPFDGEGGILAHAFSPGPGIGGDVHFDDEETWTTNGSGYNLLPVAVHEFGHALGLSHSSDPGAIMFPAYNFGLHSVLQLSYQDVKDIKEMYGERSTSPDLLPPKTPDRCDPDLSFDAVTGMQQELVFFKDRFIWRVHPSFDEIRITLITSLWTEIPADIDAAYENTNKNSILVFK

>Dr_LOC553390_MMPLf_MMP

MKNSSAVRRQDPQSPTSPHQQREPPRLYGGDIHYTPASPEKRPFPSAEAYLSQFYRDSKAAKTLGRMFVSNLENELKAMQSFFGLEVTGQLDSNTLTTMKLPRCGVTDVARFGHFEGKPRWKQSVVTYRITEYSTQLSQREVDSTIAKAFQLYSDVIPVDFKQIFSGTADIMILFKGGHHGDFYPFDGPNGVLAHANSPGPEQGGDTHFDDDEKWSLSSHNINLLLVAAHEFGHALGLDHSRDSSALMYPTYRYVSTNAYTLPRDDRLGVQALYGVRASTNKPEPKPEPKPNPQPAPSPPEPCKRDLIFDAATRIRGELYFFKDR

>Dr_LOC557654_MMPLg_MMP

HGDGYPFDGPNGFLAHAYPPYEGVGGDAHFDDDETFSYRSPQYYTLFSVAAHEFGHSLGLGHSRDPGALMYPTYVYRDMDRFILPRDDVNGIQSLYGPNTDVNTDDSKPTPPVTPNTCDPNLVLDAVTMLRGEIMFFKNSFFWRSYPQSPDVELQLIQSFWPEIPDNIDAAFESVIQDKVFLIKGEKVWALYGYDIVQGYPKSLSMFRLRKNVKKIDAVLYKEDSNTILFFANNQVYSYNERMKKMDKGFPKPVQKVFPGMTGKVTAAFQYRGFNYLFSGSKMLEFGSNNKLLRVLNNNYFLPCK

>Dr_mmp13_MMPLh_MMP

MKTCFRLCVFITLLFSGHSSPIAPPAGGDQDTLAENYLTRLYGLPKPAQNPSPSGGEKRSSDVSLRLKEMQQFFKLKVSGKLDQETLEVMKKPRCGVPDIKAYSTFAGDYKWKKHQLTYRIENYTPDMSVAEVDDSISRALKVWADVTPLRFTRIYSGTADIMISSI

>DrTIMP2_TIMP2a_TIMP

MKSVRSCIGTLVVLVLFRVGEIAEACSCSPVHPQQAFCNADVVIRAKVVGKKEVDSGNDIYGNPIKRIQYEIKQIKMFKGPDRHIDVVFTAPSSAVCGVTNLDTNGKKEYLISGKAEANGKMHVTLCDLIMPWESMSATQKKSLSQRYQMGCDCKITRCATFPCEISAPEECLWTDWVTEKIIHGRQSDHYACIKRGDGSCAWYRGVAPPKKEFLEVEDP

>DrTIMP2L_TIMP2b_TIMP

MSMSRSVPLLLLVLCGATDFAGACSCSPEHPQQAYCNADVVIRAKVVGRKEVVTGNDAYGYPIKMIRYDVKQLKMFKGPNGEIDTIFTGPSSALCGVNLESNGKQEYLITGNLNSNGTLRINLCDYIESWESLSLTQKKSLGPRYQMGCDCKIVQCPIIPCSISEPVECLWTDWVLEGTVQGTQAQHYTCVKRSDSSCAWYRGSTPPKKEFMDIEEP

>DrLOC559979_TIMP2c_TIMP

MCQLLCRMLADLTKKAKEKSDSCKRSTCRKWDLVIRAKVVGKKEVDSGNDIYGNPIKRIQYEIKQIKMFKGPDRHIDVVFTAPSSAVCGVTNLDTNGKKEYLISGKAEANGKMHVTLCDLIMPWESMSATQKKSLSQRYQMGCDCKITRCATFPCEISAPEECLWTDWVTEKIIHGRQSDHYACIKRGDGSCAWYRGVAPPKKEFLDVEDP

>DrLOC568824_TIMP2d_TIMP

MSYLASMKGSVGGVCLSDGHNARNVLEITVIVNLHLQGTSTLTVLRAEITGEKIRRREDINPMYGLGKIQYEVQVIKVFKGSDRIKDLQHVYTHEMSSMCGIRLNRGQYLLSGSMMSEGFFVTLCDFVEHWDRLSLTQKKNLKYRYQMGCNCTISICTEQPCHPKVKNECILTDWSSLWPFEDGEPVHEYACIRHSDGSCSWHEGGTSAMVLDEEPIPGQDPEEIKYKINVLQVFKGAEHAGIEYLHTASDGAMCGIRLRPGIYLLAEKGDAQTEADLQRRGGEESEVKMMKMKMCVTAGLLFLMCVCLKEHMLEACSCGPAHPQELFCHADVVFKARVLSMKLIKSMYPGEGYFKFNINITKMYKGFEHAGIKHFFTPEDEGMCGISLTRSVYLFSGKLVSRHEGHAKKSLTVDLCDIVKHWSRLSKTEKKILSVHRKACNCQVSHCYIEPCDENKCYLRDIYDPDEGSQSICMANSTGSCRWINA
