# Supplementary material for: The evolution of the vertebrate metzincins; insights from Ciona intestinalis and Danio rerio
Source: BMC Evol Biol. 2007 Apr 17;7:63. doi: 10.1186/1471-2148-7-63 (PMC1867822; doi:10.1186/1471-2148-7-63)
Supplement: Additional file 4 — Supplementary figures S1-S6. Initial guide-tree phylogenetic analyses of the ADAM and MMP gene families, full sub-group analyses of the ADAM gene family and diagrammatic representation of a tandem duplication located on the Danio rerio chromosome 2 [file 1471-2148-7-63-S4.doc]

**Additional file 4**

**Supplementary figures S1-S5:**

The phylogenetic trees were produced using the Neighbor Joining method. The values on the tree nodes are neighbor joining percentage bootstrap values. The trees are mid-point rooted. The scale bars correspond to the number of amino acid replacements per site (horizontal axis). Gene names are coloured as vertebrate – green; invertebrate chordate – blue; invertebrate – red. Gene names can be linked to the accessions shown in additional file 1.

**Figure S1** Phylogenetic relationship guide tree of genes forming the ADAM gene family


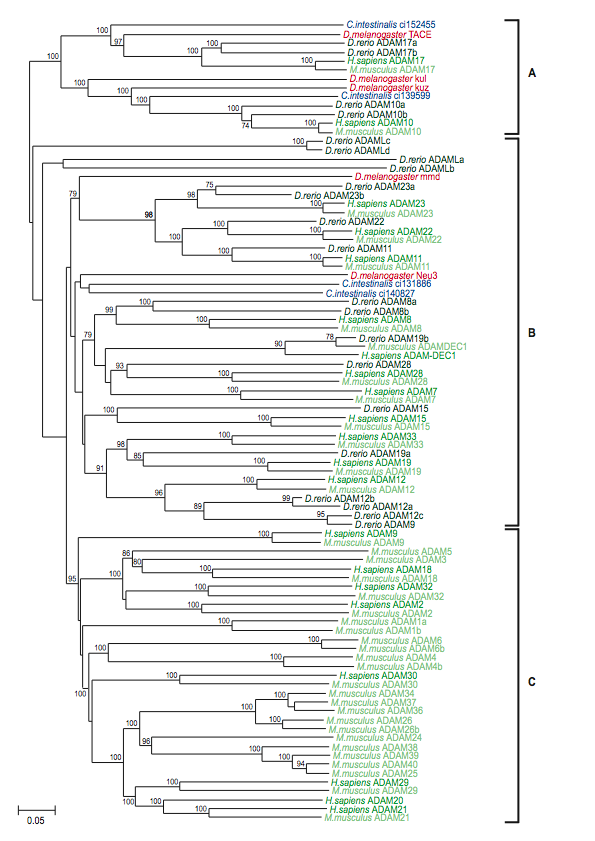


**Figure S2** Phylogenetic relationships of genes forming the A subgroup of the ADAM gene family


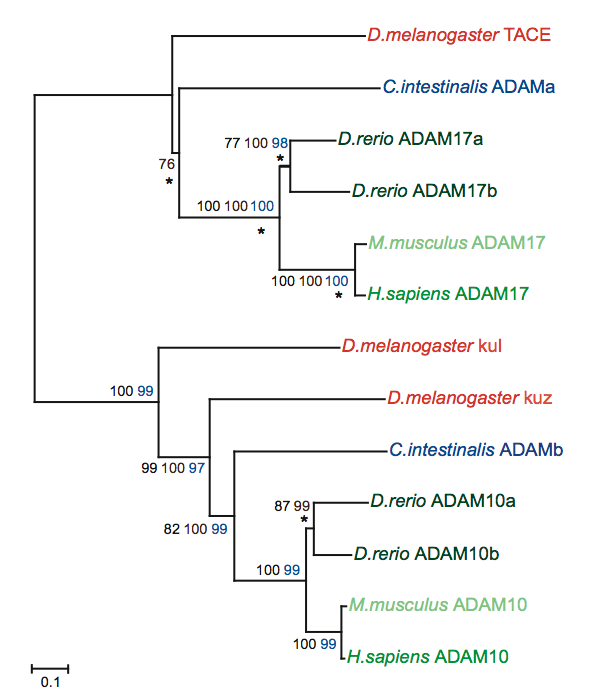


**Figure S3** Phylogenetic relationships of genes forming the B subgroup of the ADAM gene family.

A. Phylogenetic analysis of the ADAM B subgroup. B. Cladogram of the ADAM B sub-group.

Further analysis on individual sub-fragments of the B subgroup found zebrafish ADAM19b (LOC571252) to group with *H. sapiens* ADAM19 at α; zebrafish ADAM12b (LOC558872) and ADAM12c (LOC561244) to groups with *D. rerio* ADAM12Aat β and zebrafish ADAM9 (zgc101824) at χ.


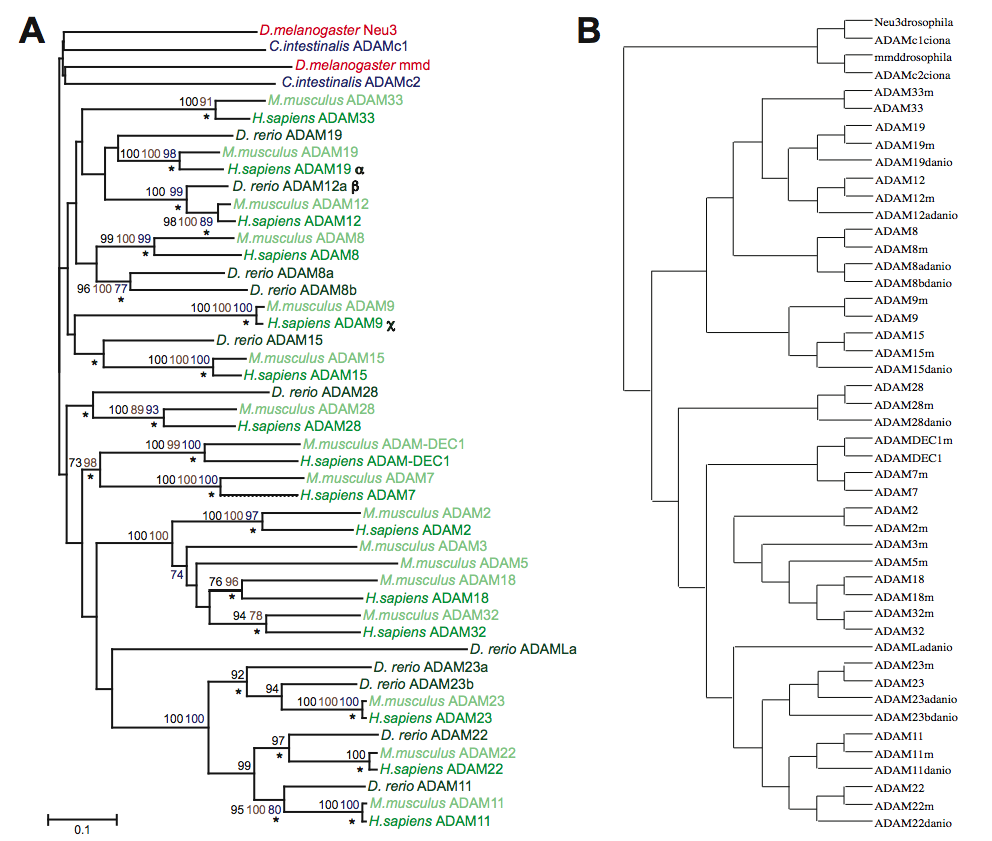


**Figure S4** Phylogenetic relationships of genes forming the C subgroup of the ADAM gene family


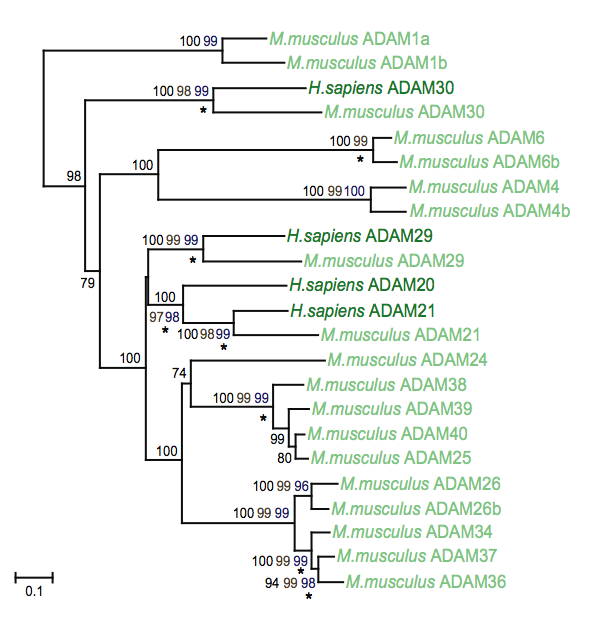


**Figure S5** Phylogenetic relationships of genes forming the MMP gene family


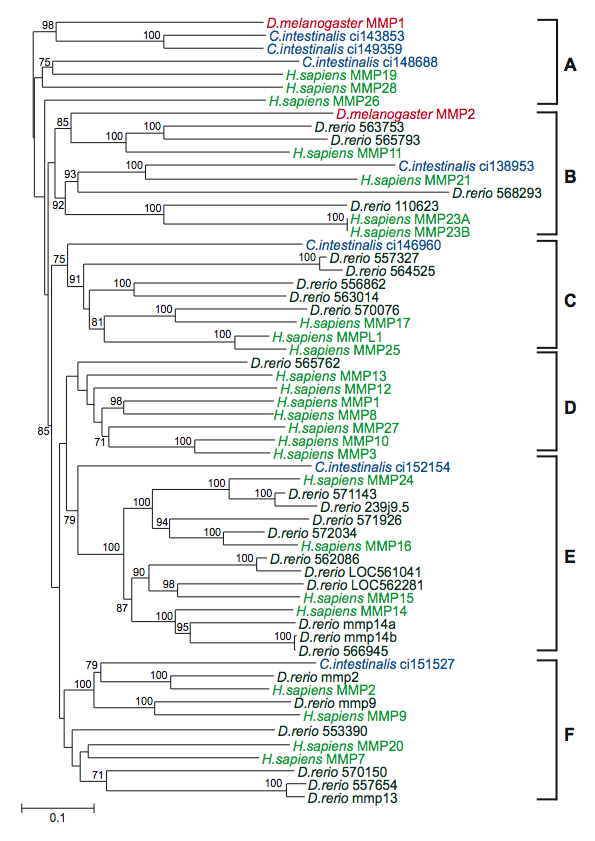


**Figure S6** Tandem duplication of a part of the *D. rerio* chromosome 2


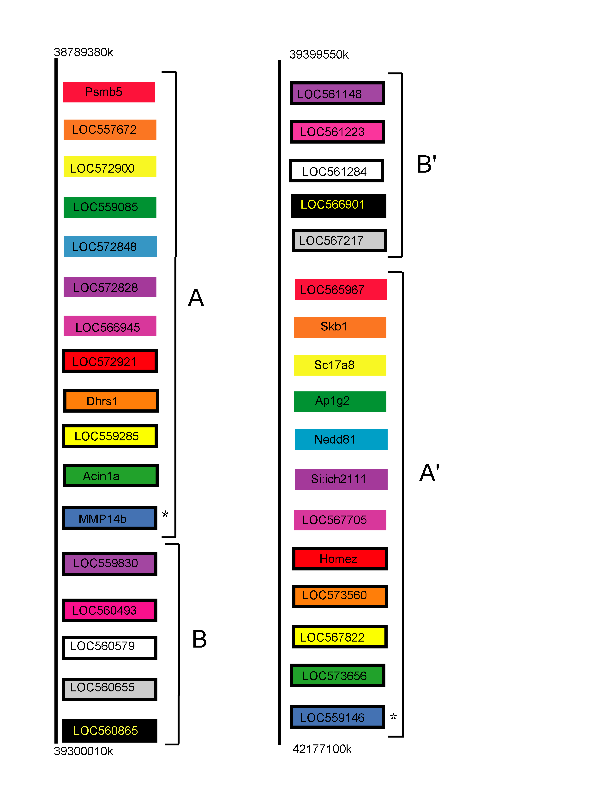


Boxes of the same colour and outline are duplicate genes. Gene order is conserved in group A/A’ and group B/B’. Genes part of the MMP supergene family are highlighted (*).
